# Conditionally Activatable Pyroptosis‐Inducing Agents for Cancer Therapy

**DOI:** 10.1002/smsc.202300135

**Published:** 2023-12-15

**Authors:** Yongkang Zhang, Yaming Wang, Hao Zhang, Fan Qi, Eryan Li, Changhua Li

**Affiliations:** ^1^ State Key Laboratory of Medicinal Chemical Biology College of Pharmacy Key Laboratory of Functional Polymer Materials of Ministry of Education College of Chemistry Nankai University Tianjin 300071 China

**Keywords:** cell pyroptosis, immunogenic cell death, photosensitizers, stimuli-responsiveness, tumor immunotherapy

## Abstract

Pyroptosis is a recently defined form of immunogenic cell death that shows great promise in cancer immunotherapy. However, almost all small‐molecule pyroptosis‐inducing agents (PyAs) reported to date indiscriminately induce pyroptosis in multiple cell types, leading to off‐target pyroptotic death of normal and immune cells. One promising approach to addressing this biosafety issue is the design of conditionally activatable PyAs that specifically respond to disease biomarkers. Herein, a general solution for facilely tailoring and synthesizing activatable PyAs based on a newly developed class of photoactive PyAs, termed PyPSs, is reported. The unique structurally encoded properties of PyPSs, including endo reticulum targeting, hypoxia tolerance, and sensing properties, excitingly meet a demanding set of performance requirements for constructing conditionally activatable PyAs for cancer immunotherapy. Based on PyPS‐1 scaffold, hypoxia‐activatable PyPS‐NF as a proof‐of‐concept example is prepared, demonstrating specific hypoxia‐activated pyroptotic cell death and favorable immunotherapeutic efficacy of solid tumors. Herein, a general design strategy for tailoring activatable PyAs to precisely control GSDME‐mediated cell pyroptosis is established, with great potential to advance cancer immunotherapy.

## Graphical abstract

A unified design to prepare hypoxia‐tolerant and stimuli‐activatable pyroptosis‐inducing agents (PyAs) is established, opening an avenue to tailor and synthesize PyAs capable of inducing pyroptosis in a controlled manner, which is essential for advancing pyroptosis‐inducing cancer immunotherapy.

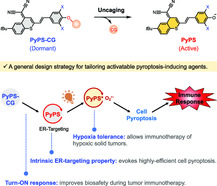

## 1 Introduction

Pyroptosis triggered by the inflammatory caspase‐mediated cleavage of gasdermin (GSDM) proteins is a recently defined immunogenic form of programmed cell death.^[^ ^1^, ^2^, ^3^, ^4^, ^5^ ^]^ It is characterized by plasma membrane pore formation, cell swelling with large bubbles, and massive leakage of cellular proinflammatory cytokines. Compared with traditional apoptosis and necrosis, pyroptosis is more liable to trigger cellular inflammation, showing great potential in cancer immunotherapy.^[^ ^6^, ^7^, ^8^, ^9^, ^10^ ^]^ Key to this effort was the development of multiple pyroptosis‐inducing agents (PyAs) that have demonstrated the efficacy of inflammatory pyroptosis in cancer therapy.^[^ ^11^, ^12^, ^13^, ^14^, ^15^ ^]^ Compared with conventional chemotherapeutic drug‐based PyAs, photoactive PyA possesses multiple significant advantages such as spatiotemporally controllable activation, no cross‐resistance with chemotherapeutic drugs, and minimal invasiveness. Canonical photoactive PyA requires oxygen to produce reactive oxygen species (ROS) to induce cell pyroptosis. However, severe O_2_ deficiency prevalent in fast‐growing solid tumors, i.e., tumor hypoxia,^[^ ^16^ ^]^ substantially reduces the treatment outcome. Therefore, the development of photoactive PyAs with hypoxia tolerance is an urgent need.

In addition, since the GSDM proteins are expressed in various normal tissues and immune cells in addition to cancer cells, the vast majority of photoactive PyAs reported so far induce pyroptosis indiscriminately, leading to off‐target pyroptotic death in normal tissues and cells.^[^ ^3^, ^10^ ^]^ Therefore, the development of PyAs that can specifically induce pyroptosis in a controlled manner is expected to improve biosafety and is crucial for advancing pyroptosis‐inducing cancer immunotherapy.^[^ ^17^, ^18^, ^19^ ^]^ One promising approach is to engineer conditionally activatable PyAs that can specifically respond to disease biomarkers. Considering the complex and diverse tumor microenvironment, establishing a general platform to tailor oxygen‐tolerant and stimuli‐activatable PyAs in response to variable tumor biomarkers is a timely exercise.

In this work, we have identified a unique class of photoactive PyAs, termed PyPSs, with fascinating structurally encoded properties that meet a demanding set of performance requirements for constructing conditionally activatable PyAs for cancer immunotherapy. First, the intrinsic endo reticulum (ER)‐targeting property of PyPSs enables significant GSDME‐mediated pyroptosis. Second, PyPSs‐induced pyroptosis occurs under both normoxic and hypoxic conditions, and this remarkable hypoxia tolerance allows immunotherapy of solid tumors characterized by hypoxia. Finally, and most strikingly, flexible phenol caging/uncaging chemistry allows one to tailor multiple conditionally activatable PyAs via installing PyPSs with various caging groups (CG) for precise cancer immunotherapy. Based on PyPS‐1 scaffold, we prepared a hypoxia‐activatable paradigm, PyPS‐NF, showing specific hypoxia‐activated pyroptotic cell death and antitumor efficacy. This study establishes a universal strategy, for the first time, to construct a variety of conditionally activatable PyAs, thereby providing a rich molecular toolbox for the precise control of cell pyroptosis.

## 2 Results and Discussion

### 2.1 Synthesis and Photophysical/Chemical Properties of PyPS Molecules

**Figure 1:**
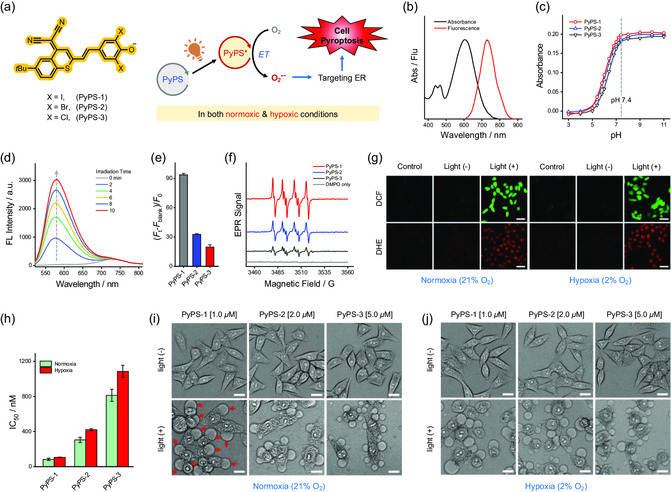
Photophysical/chemical properties of PyPS molecules and their ability to generate superoxide anion radical (O_2_^•−^) and induce pyroptosis in living cells. a) Schematic illustration of chemical structures of PyPS molecules and their photosensitivity under both normoxic (21% O_2_) and hypoxic (2% O_2_) conditions. b) UV–vis absorption and fluorescence emission of PyPS‐1 in aqueous medium at pH 7.4. c) pH‐dependent changes in the absorbance of the ICT‐bands of PyPS molecules. d) Time‐dependent fluorescence emission spectra of DHE within 2.5 μm aqueous solution (pH 7.4, 40 μg mL^−1^ DNA‐ct) of PyPS‐1 upon light irradiation. e) Fluorescence enhancement of DHE induced by the photogenerated O_2_^•−^ of PyPS molecules (*n* = 3). f) EPR spectra of PyPS molecules in the presence of DMPO upon light irradiation. g) Confocal fluorescence imaging of photogenerated ROSs by PyPS‐1 in MCF‐7 cells under (left) normoxic and (right) hypoxic conditions by using DCF‐DA and DHE as the general ROS and O_2_^•−^ fluorescence indicators, respectively. Scale bar: 50 μm. h) IC_50_ values of different PyPS molecules under normoxia and hypoxia (*n* = 4). i,j) Representative phase contrast images of MCF‐7 cells incubated with different PyPS molecules in dark or upon light irradiation under normoxic (i) and hypoxic (j) conditions. The red arrows indicate the characteristic large bubbles accompanying pyroptosis. Scale bar: 20 μm. The light source was a Xe lamp (490–700 nm) with the power fixed at 5 and 10 mW cm^−2^ for solution and cell experiments, respectively.

Three putative PyAs (PyPS‐1, PyPS‐2, and PyPS‐3) with D‐*π*‐A structure (**Figure** **1**a) were synthesized via Knoevenagel condensation (see the Supporting Information). Sulfur‐substituted dicyanomethylene‐4H‐chromene was chosen as a typical electron‐accepting group, in which the sulfur atom is expected to promote the intersystem crossing (ISC) process and thus enhance the generation of T_1_ state.^[^ ^20^, ^21^, ^22^, ^23^ ^]^ Phenolate groups substituted by halogen atoms at the ortho‐positions were adopted as electron‐donating moieties. As depicted in Scheme S1, Supporting Information, the phenolic hydroxyl group has to be ionized into an anionic phenate state to promote the intramolecular charge transfer (ICT) process,^[^ ^24^, ^25^ ^]^ which is the prerequisite for achieving photoactivity. Gratifyingly, as shown in Figure 1b and Figure S2, Supporting Information, all three PyPSs exhibited intense visible ICT bands and fluorescence emission at physiological pH 7.4. This is due to the pronounced electron‐withdrawing −I halogen effect of the iodine substituents, which significantly reduced the *pK*_a_ value of the phenolic hydroxyl group (Figure 1c, Figure S3, Supporting Information, and **Table** **1**).^[^ ^26^, ^27^ ^]^

**Table 1:** Properties of three PyPS molecules

Samplea)	*λ*_abs_ [nm]	*ε* [M^−1^ cm^−1^]	*λ*_em_ [nm]	*Φ*_fl_ [%]	*pK*_a_
PyPS‐1	603	38 070	730	2.03	5.23
PyPS‐2	585	35 440	724	2.19	5.76
PyPS‐3	573	44 180	685	14.1	5.78

a) The experiments for determining fluorescence quantum yields and *pK*_a_ values were performed in citric acid/Na_2_HPO_4_ buffer solutions; the other data were collected in 10 mM PBS (pH 7.4). The results represent the average of three independent experiments.

The photochemical properties of PyPS molecules were then studied. QDPBF (a water‐soluble 1,3‐diphenylisobenzofuran derivative) was used to assess the photosensitivity of PyPSs in generating ROS (Scheme S2, Supporting Information),^[^ ^28^ ^]^ and the obvious reduction of QDPBF absorption indicated their high efficiency in generating ROS (Figure S4, Supporting Information). Notably, the photosensitivity of PyPS‐1 was estimated to be approximately 2.4 times greater than that of methylene blue (MB), a widely used commercial photosensitizer, by recording the absorbance decrease of QDPBF at 415 nm (Figure S4f, Supporting Information). By further using ABDA as the specific ^1^O_2_ indicator (Scheme S3, Supporting Information), and only a small amount of ^1^O_2_ was detected during photosensitizing process of PyPSs (Figure S5, Supporting Information). In sharp contrast, as shown in Figure 1d and Figure S6, Supporting Information, PyPSs induced a drastic increase in the fluorescence emission of dihydroethidium (DHE), a fluorogenic probe of O_2_^•−^ (Scheme S4, Supporting Information), and the fluorescence increased in the order of PyPS‐3 < PyPS‐2 < PyPS‐1 (Figure 1e). EPR (electron paramagnetic resonance) spectroscopy analysis further identified the photogenerated O_2_^•−^ (Scheme S5, Supporting Information), as evidenced by the characteristic 1:1:1:1 quartet signal attributed to the DMPO‐O_2_^•−^ adduct, i.e., DMPO‐OOH (Figure 1f).^[^ ^29^ ^]^ Similar to the DHE assay, the EPR signals of DMPO‐OOH increased in the order of PyPS‐3 < PyPS‐2 < PyPS‐1. The earlier results indicated that PyPSs mainly underwent type‐I pathway, which is less oxygen‐dependent compared to conversional type‐II pathway.^[^ ^30^, ^31^ ^]^

The difference in photosensitivity between the three PyPSs should be attributed to the diverse heavy atom effects of their substituted halogens (where I > Br > Cl).^[^ ^32^ ^]^ Time‐dependent density functional theory (TD‐DFT) calculations was then performed to elucidate the type‐I mechanism of PyPS‐1. As shown in Figure S7a, Supporting Information, the lowest energy gap (Δ*E*_ST_) between the singlet excited state (S) and triplet excited state (T) of PyPS‐1 was calculated to be 0.09 eV, which is much less than 0.3 eV, facilitating the singlet‐triplet ISC process to form ^3^PyPS‐1* upon photoexcitation.^[^ ^33^ ^]^ On the other hand, the electron‐rich nature of anionic PyPS‐1 promotes the formation of PyPS‐1^•−^ from ^3^PyPS‐1*.^[^ ^34^, ^35^, ^36^ ^]^ The formed PyPS‐1^•−^ then transfers the electron to oxygen to generate O_2_^•−^.^[^ ^30^, ^31^ ^]^ The Gibbs free energy (Δ*G*_1_) of the electron transfer process was calculated to be −13.88 kcal mol^−1^, indicating an energy favorable reaction (Figure S7b, Supporting Information). Given the remarkable type‐I photosensitivity, we then assessed the photodynamic therapy (PDT) performance of PyPS‐1 in living cells.

The weak but visible fluorescence of PyPSs enabled the fluorescence monitoring of its internalization by MCF‐7 cells. Taken PyPS‐1 as example, as the molecule was uptaken by MCF‐7 cells, its fluorescence increased with time and reached a plateau at about 2 h (Figure S8, Supporting Information). Therefore, in subsequent cell experiments, the incubation time of PyPSs was fixed at 2 h. After confirming the low dark cytotoxicity of PyPS‐1 (Figure S9a, Supporting Information), we then assessed its photosensitivity in MCF‐7 cells. Dichlorofluorescein diacetate (DCF‐DA) and DHE were employed as universal ROS and specific O_2_^•−^ fluorescent indicator, respectively.^[^ ^20^, ^34^ ^]^ As expected, cells treated with light alone or PyPS‐1 alone showed negligible DCF or DHE fluorescence (Figure 1g). In contrast, in the group treated with PyPS‐1/light, intense intracellular DCF and DHE fluorescence were observed, indicating significant photoactivity of PyPS‐1 in generation of O_2_^•−^. Encouragingly, PyPS‐1 remained strong photoactivity in cells incubated under hypoxic condition (2% O_2_). These observations of hypoxia‐tolerant photoactivity of PyPS‐1 were in good agreement with the results of ROS studies in solution.

Emboldened by the earlier findings, we subsequently assessed the potential of PyPS‐1 to ablate cancer cells. Live/dead cell staining assay with Calcein AM and propidium iodide (PI) was carried out. As shown in Figure S10, Supporting Information, under both normoxic and hypoxic conditions, MCF‐7 cells treated with 1 μm PyPS‐1 were stained by PI after light irradiation, demonstrating the remarkable PDT efficiency of PyPS‐1. MTT assay of PyPS‐1 further demonstrated its effective PDT outcome under both normoxic and hypoxic conditions (Figure S9a, Supporting Information), with IC_50_ values as low as 0.083 and 0.106 μm, respectively (Table S1, Supporting Information). Although type‐I photosensitizers have a certain tolerance to hypoxia, their ability to generate ROS under hypoxic conditions is still slightly weaker than under normoxic conditions.^[^ ^20^, ^30^ ^]^ Remarkably, under hypoxic condition, the IC_50_ value of PyPS‐1 was 80‐fold lower than that of MB (Figure S9 and Table S1, Supporting Information), owing to its superb hypoxia‐tolerant type‐I photoactivity. Notably, MTT assay of PyPS‐2 and PyPS‐3 also demonstrated their good biocompatibility and effective PDT outcome (Figure S9, Table S1, Supporting Information). Not surprisingly, PyPS‐1 displayed the strongest photocytotoxicity among all PyPSs no matter in normoxia or hypoxia (Figure 1h, Table S1, Supporting Information), which is consistent with the photosensitivity results in solution.

Significantly, the dying MCF‐7 cells evoked by PDT exhibited typical features of pyroptosis with distinct cell swelling and large bubbles from the plasma membrane (Figure 1i). It was observed that the size and number of bubbles generated on the plasma membrane of cells treated with different PyPS molecules were positively correlated with their photosensitivity. When the concentrations of PyPS‐2 and PyPS‐3 were reduced to the same 1 μM as that of PyPS‐1, the cells treated with them produced significantly fewer and smaller bubbles on the plasma membrane than the PyPS‐1 group (Figure S11, Supporting Information). Annexin V/PI staining for MCF‐7 cells was then performed. As shown in Figure S12, Supporting Information, MCF‐7 cells after PyPS‐1/light treatment proceeded directly to the annexin V and PI double‐positive stage, in line with the previously reported results of cell pyroptosis.^[^ ^8^, ^11^ ^]^ PyPS‐1‐triggered cell pyroptosis remained prominent under hypoxic conditions owing to its type‐I photoactivity (Figure 1j). Notably, light irradiation alone had no obvious effect on cells under both normoxic and hypoxic conditions (Figure S13, Supporting Information). Moreover, this PDT‐induced pyroptotic cell death is prevalent in other cancer cell lines, such as A549, HeLa, A375, and HepG2 cells (Figure S14, Supporting Information).

### 2.2 The Mechanism Underlying Pyroptosis Activation

**Figure 2:**
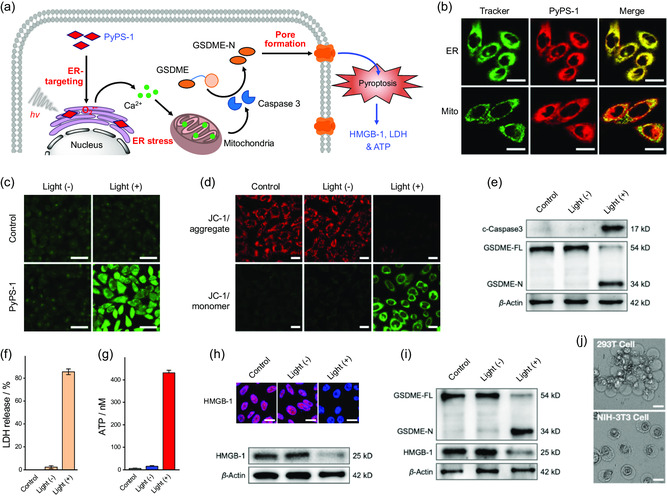
The mechanism underlying pyroptosis activation. a) Schematic illustration of the mechanism of the cell pyroptosis triggered by PyPS‐1. b) Colocalization studies of PyPS‐1 toward ER in MCF‐7 cells; cells were incubated with PyPS‐1 (2.0 μm) for 1 h. Pearson's correlation coefficients (PCCs) for ER and Mito are 0.95 and 0.69, respectively. c) Fluorescence imaging of Ca^2+^ ions in MCF‐7 cells after different treatments using Fluo‐3 AM as a fluorescent Ca^2+^ indicator. d) Evaluation of the mitochondrial damage of MCF‐7 cells treated with PyPS‐1 (1 μm) under light irradiation in normoxic conditions by using JC‐1 as the fluorescent indicator. e) Western blotting of the cleavage of GSDME by caspase‐3 in MCF‐7 cells after treatment of PyPS‐1/light under normoxic conditions. c‐Caspase‐3, active Caspase‐3 after cleavage of pro‐Caspase‐3; GSDME‐FL, full‐length GSDME; GSDME‐N, the N‐terminal cleavage product of GSDME. f–h) Investigation of LDH (*n* = 4) (f), ATP (*n* = 4) (g), and HMGB‐1 (h) release in MCF‐7 cells after PyPS‐1/light treatment. i) Western blotting of the cleavage of GSDME in MCF‐7 cells after treatment of PyPS‐1 and light under hypoxic conditions. j) Representative phase contrast images of healthy 293 T or NIH‐3T3 cells after treatment of PyPS‐1/light. Scale bar in (c): 50 μm. Scale bars in other cell images: 20 μm. Light source: Xe lamp (490–700 nm, 10 mW cm^−2^).

In view of the unusually drastic cell pyroptosis, we moved to explore the mechanism underlying pyroptosis activation (**Figure** **2**a). Growing evidence suggests that organelle stress can induce pyroptosis,^[^ ^18^, ^37^ ^]^ so we speculated that our PyPSs might specifically target and attack certain organelles. Through confocal laser scanning microscope, we were surprised to find that the three PyPSs were mainly localized in ER of MCF‐7 cells with Pearson's correlation coefficients above 0.85 (Figure 2b and S15, Supporting Information). Colocalization imaging of PyPS‐1 in different z‐axis sections was then performed, and the result showed that the spatial distribution of PyPS‐1 is highly consistent with the specific ER probe (ERTG; Figure S16, Supporting Information), further demonstrating that PyPS‐1 is specifically localized to the ER. This specific ER‐targeting property of PyPSs was also observed in other cancer cell lines, such as HeLa and HepG2 cells (Figure S17, Supporting Information). Notably, photosensitizers that can achieve specific ER targeting without chemically attaching additional targeting groups have been rare reported, and this structurally encoded ER targeting property can effectively reduce tedious chemical synthesis and molecular size.

Cellular uptake pathway of PyPS‐1 was then investigated. As shown in Figure S18, Supporting Information, the cellular uptake of PyPS‐1 significantly decreased by 85.5% when incubating at 4 °C, suggesting an energy‐dependent pathway of the cellular uptake. In addition, endocytosis inhibitor assays were employed, including genistein (GEN, an inhibitor of caveolae‐mediated endocytosis), methyl‐*β*‐cyclodextrin (M*β‐*CD, an inhibitor of lipid raft‐mediated endocytosis), chlorpromazine (an inhibitor of clathrin‐mediated endocytosis), 5‐(*N*‐ethyl‐*N*‐isopropyl)‐amiloride (an inhibitor of macropinocytosis), and cytochalasin B (CytB, an inhibitor of phagocytosis and/or macropinocytosis). Among these inhibitors, GEN and M*β‐*CD resulted in a 69.9% and 66.6% decrease in cellular uptake, respectively, indicating that PyPS‐1 was uptaken mainly via the caveolae‐mediated endocytosis pathway (Figure S18, Supporting Information). The earlier finding echoed previous reports that caveolae‐mediated endocytic pathways are capable of delivering cargos to the ER.^[^ ^38^, ^39^ ^]^

The ER‐targeting property of PyPS‐1 should enable concentrated ROS attacks, leading to severe ER stress, as demonstrated by the observation of a transfer of free Ca^2+^ ions from the ER to the cytoplasm using a fluorescent Ca^2+^ indicator Fluo‐3 AM (Figure 2c and S19, Supporting Information).^[^ ^40^ ^]^ It has been reported that Ca^2+^ release caused by severe ER stress can promote ER‐mitochondria Ca^2+^ flux and thus mitochondrial Ca^2+^ overload, leading to decreased mitochondrial membrane potential and caspase‐3 activation.^[^ ^40^, ^41^, ^42^ ^]^ JC‐1 staining demonstrated that PyPS‐1 lowered mitochondrial membrane potential upon light irradiation (Figure 2d),^[^ ^43^ ^]^ and Western blotting showed that caspase‐3 was activated in these cells and triggered GSDME‐mediated pyroptosis (Figure 2e). In contrast, a caspase‐3 inhibitor Z‐DEVD‐FMK attenuated caspase‐3‐triggered GSDME cleavage, thereby inhibiting cell pyroptosis (Figure S20a,b, Supporting Information).^[^ ^8^, ^44^ ^]^ It echoed the previous reports that GSDME can convert caspase‐3‐mediated apoptosis to pyroptosis.^[^ ^7^, ^8^ ^]^ Importantly, leakage of cellular contents including lactate dehydrogenase (LDH), ATP, and nuclear high‐mobility group box 1 protein (HMGB‐1) was also demonstrated (Figure 2f–h).^[^ ^45^, ^46^ ^]^ GSDMD is another well‐studied executor of pyroptosis, and it has been reported that ER‐targeted PDT could induce GSDMD‐mediated cell pyroptosis.^[^ ^47^ ^]^ However, no GSDMD cleavage was observed after PyPS‐1/light treatment via Western blotting (Figure S20c, Supporting Information). Collectively, this work demonstrates, to our knowledge, the first example of significant GSDME‐mediated pyroptosis initiated by ER‐targeted PDT.

In addition, under hypoxic conditions, PyPS‐1‐triggered cell pyroptosis via the same mechanism as under normoxic conditions (Figure 2i, S21 and S22, Supporting Information). It is nevertheless noteworthy that PyPS‐1‐induced pyroptosis was indiscriminate, as the GSDM proteins are expressed in various cell types.^[^ ^3^, ^10^ ^]^ Distinct pyroptosis occurred in healthy 293 T and NIH 3T3 cell lines following PDT treatment with PyPS‐1 (Figure 2j). Likewise, PyPS‐2 and PyPS‐3 also induced significant pyroptosis in these healthy cells (Figure S23, Supporting Information). Therefore, from the perspective of cancer treatment, adequate derivatizing PyPS‐1 with function of pyroptotic killing specific to cancers rather than normal tissues is pivotal.

### 2.3 Chemical Design of Activatable PyAs

**Figure 3:**
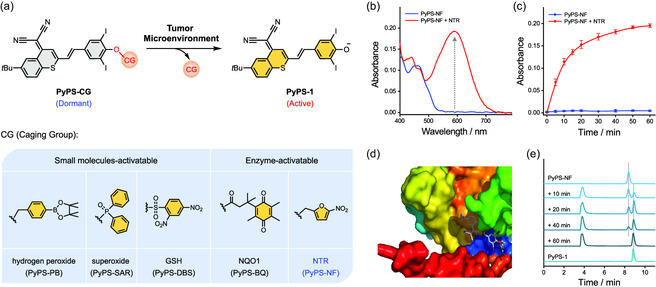
Chemical design of activatable PyAs. a) Schematic illustration of the design and activation mechanism of a panel of activatable PyPS molecules. b) UV–vis absorption spectra of PyPS‐NF (5.0 μm) before and after NTR/NADH treatment in aqueous media. c) Time‐dependent changes in absorbance at 603 nm recorded for PyPS‐NF (5.0 μm) upon incubation without (blue) or with (red) NTR/NADH at 37 °C (*n* = 3). d) Binding conformation of PyPS‐NF at the hydrophobic interspace of NTR. e) HPLC chromatograms of PyPS‐NF without treatment (top); with NTR/NADH treatment for different time intervals (middle); and PyPS‐1 only (bottom).

In light of the flexible phenol caging/uncaging chemistry,^[^ ^26^, ^48^, ^49^ ^]^ PyPS‐1 allows one to tailor multiple conditionally activatable PyAs by facilely installing PyPSs with various CG that sensitive to biospecies of interest (**Figure** **3**a). Based on PyPS‐1 scaffold, we prepared a series of activatable PyPSs (PyPS‐CG) in response to different small molecules and enzymes, including hydrogen peroxide‐activatable PyPS‐PB, superoxide‐activatable PyPS‐SAR, GSH‐activatable PyPS‐DBS, and NQO1‐activatable PyPS‐BQ (Figure S24a, Supporting Information). These synthesized PyPS‐CG molecules all exhibited excellent stimuli‐triggered turn‐on response in photoactivity, including ICT process (Figure S24b–e, Supporting Information) and photosensitivity (Figure S25, Supporting Information). Considering the ubiquitous hypoxic feature of solid tumors, hypoxia‐activatable PyPS‐NF was selected as a proof‐of‐concept example for further in vitro and in vivo antitumor evaluation.

Since tumor hypoxia is always accompanied by elevated level of nitroreductase (NTR),^[^ ^50^, ^51^, ^52^ ^]^ an NTR‐responsive 5‐nitrofuran (NF) group was installed to provide hypoxia‐activatable PyPS‐NF. As expected, the ICT process of PyPS‐NF was inhibited after etherification, as demonstrated by the vanishment of the ICT band (Figure 3b) and fluorescence quenching (Figure S26b,c, Supporting Information). Meanwhile, negligible change was observed no matter in QDPBF absorption (Figure S27a,b, Supporting Information) or DHE emission (Figure S28a,b, Supporting Information) of PyPS‐NF following light irradiation, suggesting the successful photoactivity suppression of the caged PyPS.

Then, we set out to investigate its NTR‐induced activation under hypoxic conditions. As sketched in Figure S26a, Supporting Information, NTR induces the reduction of the nitro group of the NF moiety to an amine group,^[^ ^50^, ^52^ ^]^ resulting in self‐immolation^[^ ^53^ ^]^ and release of the phenolic group. Of note, NTR‐induced reduction, and self‐immolation occurred only under hypoxic conditions,^[^ ^54^, ^55^ ^]^ which could further ensure that PyPS‐NF was specifically activated in hypoxic tumor cells. Upon adding NTR (5 μg mL^−1^) to the aqueous solution of PyPS‐NF (containing 0.5 mm NADH) at 37 °C, the characteristic absorption band at 603 nm of PyPS‐1 emerged and was restored within 40 min (Figure 3b,c). Simultaneously, a fluorogenic response of PyPS‐NF toward NTR was observed (Figures S26b,c and S29a, Supporting Information), allowing fluorescence monitoring of the activation process and imaging‐guided PDT. The response of PyPS‐NF to NTR was shown to be highly selective over other potentially interfering biomolecules (Figure S29b,c, Supporting Information). Docking calculations revealed a high affinity between PyPS‐NF and NTR with a docking energy of −9.6 kcal mol^−1^ and also indicated that PyPS‐NF tends to approach the cleft of NTR via hydrogen bonding and hydrophobic interactions (Figure 3d and S30a,b, Supporting Information). The binding affinity between PyPS‐NF and NTR was further assessed by microscale thermophoresis, which gave a dissociation constant (*K*_d_) of ≈2 μm for PyPS‐NF/NTR (Figure S30c,d, Supporting Information). High‐performance liquid chromatography (HPLC) analysis and high‐resolution mass spectrometry (HRMS) further demonstrated the NTR‐triggered full conversion from PyPS‐NF into PyPS‐1 (Figure 3e). Significantly, after exposing the NTR‐treated PyPS‐NF solution to light irradiation, remarkable ROS‐induced QDPBF consumption (Figure S27c,d, Supporting Information) and drastic O_2_^•−^‐induced DHE fluorescence enhancement (Figure S28c,d, Supporting Information) were observed, indicating restored type‐I photoactivity.

### 2.4 In Vitro Studies of Hypoxia‐Activatable PyPS‐NF

**Figure 4:**
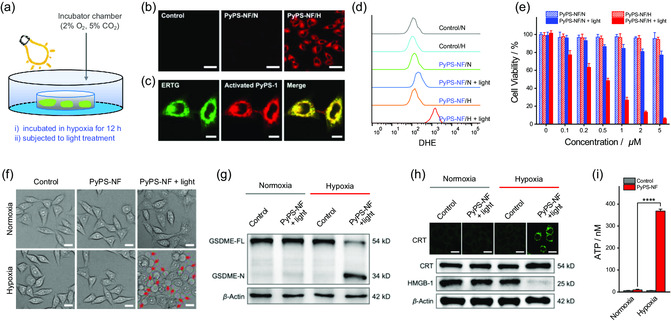
In vitro studies of hypoxia‐activatable PyPS‐NF. a) Mimic of a hypoxic environment (2% O_2_) with a hypoxia incubator chamber. b) Confocal fluorescence imaging of PyPS‐NF in response to hypoxia. Scale bar: 50 μm. c) Colocalization studies of PyPS‐NF toward ER in MCF‐7 cells. Scale bar: 10 μm. d) Flow cytometry analysis of the ROS levels in MCF‐7 cells after different treatments using DCF‐DA as ROS fluorescence probe. N and H represent normoxia and hypoxia, respectively. e) Cell viability of MCF‐7 cells incubated with PyPS‐NF in dark or upon light irradiation determined by MTT assay (*n* = 4). f) Representative phase contrast images of MCF‐7 cells incubated with PyPS‐NF in dark or upon light irradiation under normoxic or hypoxic conditions; the red arrows indicate the characteristic large bubbles accompanying pyroptosis. Scale bar: 20 μm. g) Western blotting of the cleavage of GSDME in MCF‐7 cells after treatment of PyPS‐NF and light under hypoxic conditions. h,i) Investigation of cell surface exposure of CRT and release of HMGB‐1 (h), and extracellular secretion of ATP (*n* = 4) (i). N and H in (b), (d), and (e) represent normoxia and hypoxia, respectively. Light source: Xe lamp (490–700 nm, 10 mW cm^−2^). ***p < 0.001, ****p < 0.0001 determined by Student's *t*‐test. Scale bar: 75 μm.

Encouraged by the earlier findings, we proceeded to assess the performance of PyPS‐NF in specifically activating and inducing pyroptosis in hypoxic cells (**Figure** **4**a). We quantified the NTR levels in both normoxic and hypoxic MCF‐7 cells via ELISA assay, indicating that the NTR level in hypoxic cells is approximately twofold to that in normoxic cells (Figure S31c, Supporting Information). MCF‐7 cells were incubated with PyPS‐NF under normoxia or hypoxia for 6 h and then washed. Upon imaging, the fluorescence in hypoxic cells was intense, in contrast to the slight fluorescence observed in normoxic cells (Figure 4b, S32, Supporting Information). Moreover, hypoxic cells pretreated with a known NTR inhibitor, dicumarin (DIC),^[^ ^50^ ^]^ showed a negligible fluorescence (Figure S31a,b, Supporting Information). The earlier results confirmed the specific hypoxia activation of PyPS‐NF in living cells. As expected, the converted PyPS‐1 was well anchored on ER (Figure 4c). Activatable photosensitivity of PyPS‐NF in generating ROS within MCF‐7 cells was demonstrated via flow cytometry (Figure 4d, S33 and S34, Supporting Information). MTT assay showed negligible dark cytotoxicity of PyPS‐NF in both normoxia and hypoxia (Figure 4e), indicating its good biocompatibility.

We further assessed the effect of PyPS‐NF on the precise induction of cell pyroptosis under hypoxic conditions. MCF‐7 cells were incubated with PyPS‐NF under normoxia or hypoxia, followed by exposure to light irradiation (10 mW cm^−2^) for 10 min. Both MTT and live/dead staining assays indicated the selective phototoxicity of PyPS‐NF (Figure 4e, S35a, Supporting Information). Remarkably, the MTT assay gave PyPS‐NF an IC_50_ value of only 0.38 μM under hypoxia, indicating a superb hypoxia‐specific anticancer performance of PyPS‐NF in vitro. Not surprisingly, dying MCF‐7 cells evoked by precise PDT with PyPS‐NF exhibited typical features of pyroptosis (Figure 4f), which was demonstrated to be GSDME mediated by Western blotting (Figure 4g). Collectively, the earlier results demonstrated that applying flexible hydroxyl protection/deprotection chemistry to PyPS‐1 scaffold is a simple yet robust strategy for preparing activatable PyAs.

**Figure 5:**
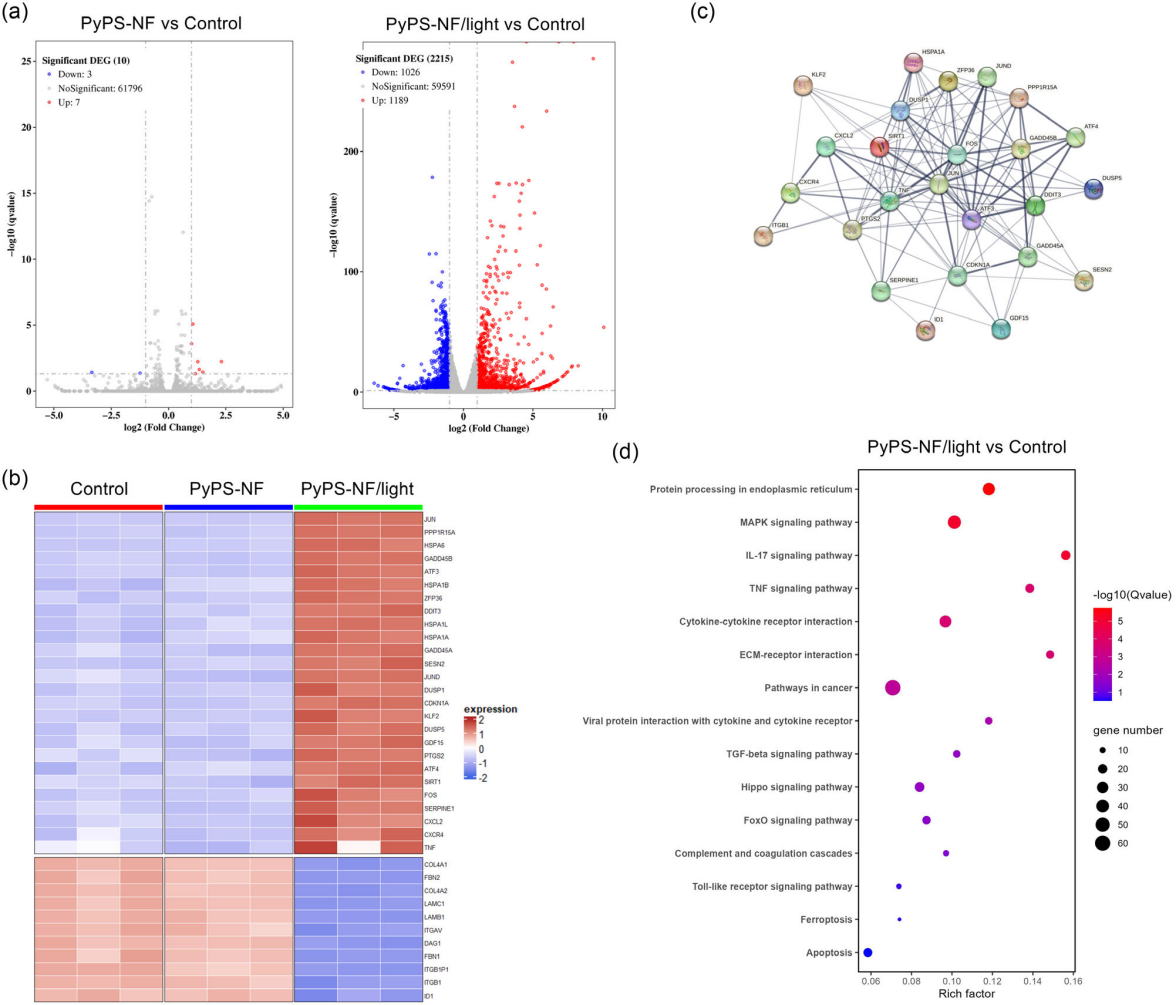
RNA sequencing analysis. a) Volcano plots displayed the differentially expressed genes (DEGs). b) Expression profiles of representative DEGs in different groups. c) Interaction network of proteins encoded by representative DEGs. d) Kyoto Encyclopedia of Genes and Genomes (KEGG) pathway enrichment analysis based on the DEGs.

Notably, accompanying cell pyroptosis, release of damage‐associated molecular patterns,^[^ ^56^, ^57^, ^58^ ^]^ including cell surface exposure of calreticulin (ecto‐CRT), release of HMGB‐1, and extracellular secretion of ATP were observed (Figure 4h,i, S35b, Supporting Information). These findings suggested that PyPS‐NF‐induced pyroptosis is highly immunogenic. To further study the immune responses induced by PyPS‐NF/light treatment, we evaluated gene expression in MCF‐7 cells after treatments via RNA‐seq. Compared with the control group, 2215 differential expressed genes (DEGs) were detected in the PyPS‐NF/light‐treated group, while only 10 DEGs were detected in the group of PyPS‐NF without light (**Figure** **5**a). In the PyPS‐NF/light‐treated group, genes associated with ER‐stress and immune system were strikingly upregulated (Figure 5b), and these genes were closely associated with each other (Figure 5c). Kyoto Encyclopedia of Genes and Genomes (KEGG) pathway enrichment analysis showed that after PyPS‐NF/light treatment, numerous DEGs were enriched in inflammatory related as well as immune response‐associated signaling pathways, such as tumor necrosis factor (TNF) signaling pathway, IL‐17 signaling pathway, MAPK signaling pathway, and cytokine‐cytokine receptor interaction signaling pathway (Figure 5d). These results suggested that PyPS‐NF‐induced pyroptosis can enhance tumor immunogenicity, and thus has great potential for antitumor immunotherapy.

### 2.5 In Vivo Studies of Hypoxia‐Activated Antitumor Efficacy of PyPS‐NF

**Figure 6:**
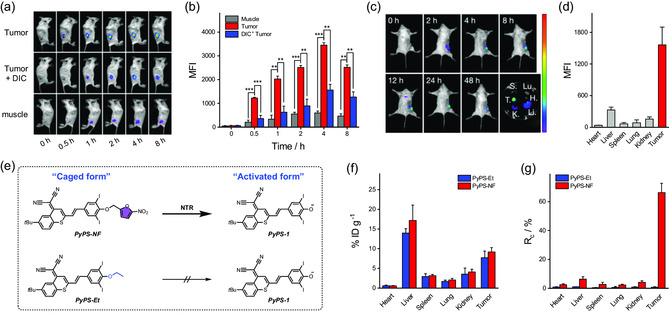
In vivo studies of hypoxia activatability of PyPS‐NF. a) Timelapse in vivo fluorescence imaging of BALB/c mice after injection of PyPS‐NF subcutaneously into tumors (top), DIC pretreated tumors (middle), or healthy hindlimb muscles (bottom). b) MFI of the tumor or muscle regions quantified from (a); *n* = 3. c) Time‐lapse in vivo fluorescence imaging of BALB/c mice after intravenous injection of PyPS‐NF and ex vivo imaging of major organs and tumors collected from mice after 48 h of injection. d) MFI of the ex vivo major organs and tumors (*n* = 3). e) Schematic illustration of the caged and activated forms of the two PyPS‐CG molecules (inert PyPS‐Et and activatable PyPS‐NF). f) In vivo biodistribution of PyPS‐CG in both caged and activated forms (*n* = 5). g) Conversion rate (*R*_c_) of PyPS‐CG from caged to activated form in different main organs and tumor tissues (*n* = 5).

Motivated by the fascinating in vitro performance, we subsequently evaluated the specific immunotherapy of hypoxic solid tumors with PyPS‐NF in vivo. As murine breast cancer cells, after PyPS‐1 mediated PDT, 4T1 cells underwent marked pyroptosis similar to MCF‐7 cells (a human breast cancer cell line) under both normoxic and hypoxic conditions (Figure S36, Supporting Information). We then assessed the hypoxia‐triggered activation of PyPS‐NF using 4T1 tumor‐bearing BALB/c mice as the model. After intratumoral injection of PyPS‐NF, a significant fluorescence increase at the tumor site was observed within 0.5 h, reaching maximum fluorescence at 4 h after injection (**Figure** **6**a,b). In contrast, tumor regions in mice pretreated with DIC showed weaker fluorescence, suggesting that PyPS‐NF was catalyzed by tumor‐overexpressed NTR to PyPS‐1. In addition, the weak fluorescence of the control group injected directly into healthy hindlimb muscles further demonstrated the specific hypoxic tumor activation of PyPS‐NF.

Encouraged by the earlier results, we continued to evaluate the performance of PyPS‐NF through systemic administration. As shown in Figure 6c and S37, Supporting Information, after intravenous injection of PyPS‐NF, the fluorescence of the converted PyPS‐1 in the xenograft tumors gradually increased over 24 h and remained at a high level for at least 48 h. Of note, the activated PyPS‐1 was mainly distributed in the solid tumor, as evidenced by the ex vivo fluorescence imaging data (Figure 6c,d). This remarkable tumor‐specific targeting of PyPS‐1 is mainly due to hypoxia‐responsiveness of PyPS‐NF, bringing great promise for overcoming the stubborn dilemmas of off‐target damage. In addition, the turn‐on fluorescence concomitant with PyPS‐NF activation allows for imaging‐guided tumor treatment, further improving therapeutic accuracy.

To further determine the on‐target activation of PyPS‐NF in tumor site, we synthesized an ethyl‐caged inert PyPS‐CG molecule (PyPS‐Et) as a control (Figure 6e). The biodistribution of PyPS‐CG (PyPS‐Et or PyPS‐NF) in main organs and tumor tissues of 4T1 tumor‐bearing BALB/c mice (*n* = 5) was then studied. Tissue homogenates after 24 h of intravenous injection of PyPS‐CG were collected and analyzed by HPLC to determine the levels of PyPS‐CG in both caged and activated forms in different tissues (Figure 6e). As shown in Figure 6f, the levels of PyPS‐Et and PyPS‐NF in tumor tissues were comparable. The difference is that PyPS‐Et exists almost exclusively in the caged form (Figure S38a, Supporting Information), whereas PyPS‐NF exists mainly in the activated form (Figure S38b, Supporting Information). Furthermore, the conversion rate (*R*_c_) of the two molecules from caged PyPS‐CG to activated PyPS‐1 in tumor tissues was calculated. PyPS‐Et was barely activated with an *R*_c_ value of 0.7%, while about 66.2% of PyPS‐NF was successfully activated (Figure 6g). The above results combined with the in vivo fluorescence data (Figure 6a–d) demonstrated the on‐target activation of PyPS‐NF in solid tumors.

**Figure 7:**
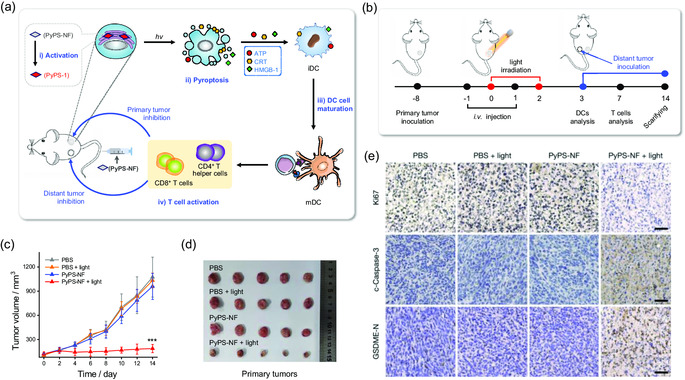
In vivo studies of antitumor efficacy of PyPS‐NF. a,b) Schematic illustrations of the specific photoimmunotherapy of hypoxic solid tumors with PyPS‐NF in vivo (a) and the schedule for tumor implantation and PyPS‐NF‐mediated photoimmunotherapy (b). c) Size of the primary tumors in different treatment groups (*n* = 5). d) Photographs recorded for the primary tumors collected after 2 weeks of observation. e) Representative IHC images of Ki67, cleaved caspases‐3 (c‐Caspase‐3), and GSDME‐N in the primary tumors 2 weeks after different treatments. Scale bar: 50 μm.

Subsequently, PyPS‐NF‐mediated photoimmunotherapy was investigated in mice inoculated with 4T1 cancer cells in the right flank as the primary tumors (**Figure** **7**a).

Compared to the bulky xenon lamp used, a portable 633 nm laser that can better penetrate animal tissue was used during in vivo experiments.^[^ ^59^, ^60^ ^]^ We first evaluated cell pyroptosis upon PyPS‐NF/laser treatment. Gratifyingly, as shown in Figure S39, Supporting Information, both hypoxic 4T1 and MCF‐7 cells after PyPS‐NF/laser treatment underwent marked pyroptosis, similar to PyPS‐NF/Xe lamp treatment. Notably, due to the serious energy loss of laser when penetrating skin and tumor tissue, the laser intensity was increased to 0.1 W cm^−2^ during in vivo experiments to obtain favorable antitumor efficacy.^[^ ^61^, ^62^ ^]^

These tumor‐bearing mice were then randomized into four groups (*n* = 5 mice): i) PBS, ii) PBS + light (633 nm, 0.1 W cm^−2^, 10 min), iii) PyPS‐NF (5 mg kg^−1^), and iv) PyPS‐NF + light, and tumor volumes were monitored every other day to assess the treatment outcome (Figure 7b). As shown in Figure 7c,d, compared with the rapid growth of the primary tumors in the three control groups (i–iii), the tumor growth in the treatment group (group iv) was significantly inhibited during the treatment. Hematoxylin and eosin (H&E) staining of the collected tumors and main organs (heart, liver, spleen, lung, and kidney) was also performed for histological analysis. Obvious extermination of cells was observed in the treatment group, showing cell shrinkage and separation (Figure S40a, Supporting Information). However, in the other control groups, no appreciable cell damage was observed. On the other hand, we compared the antitumor efficacy of PyPS‐Et/light and PyPS‐NF/light treatments (Figure S41, Supporting Information). Gratifyingly, for the group of PyPS‐NF/light treatment, a clear regression of the tumors was observed. In contrast, inert PyPS‐Et/light failed to inhibit tumor growth in view of the continuous tumor growth similar to the untreated group. These findings demonstrated the intriguing on‐target antitumor activity of PyPS‐NF in solid tumors.

Immunohistochemical (IHC) staining of the tumors showed a reduced expression of Ki67 in the PyPS‐NF/light‐treated group, indicating that the proliferation of tumor cells was significantly inhibited after treatment (Figure 7e). A significant increase of c‐caspase‐3 and GSDME‐N was observed in the tumors of the treatment group (Figure 7e), indicating pyroptosis of tumor cells after treatment. Notably, negligible cell necrosis or inflammation lesions were observed in the main organs of all mice (Figure S40a, Supporting Information), and their body weight increased steadily (Figure S40b, Supporting Information), suggesting the biocompatibility of our PyPS‐NF.

### 2.6 Systematic Immune Response after PyPS‐NF/Light Treatment

**Figure 8:**
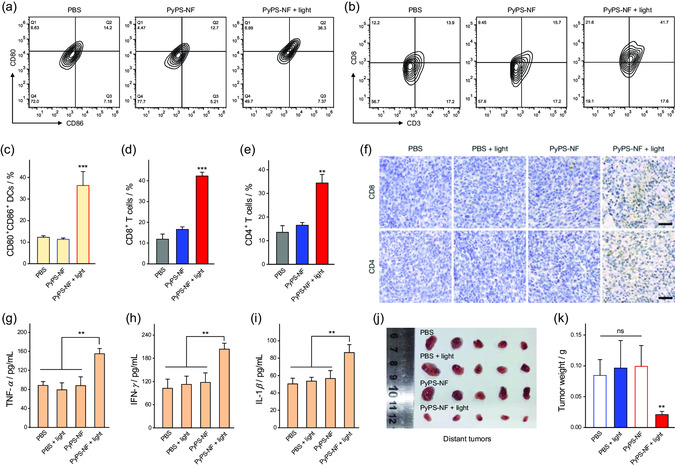
Systematic immune response. a,b) Flow cytometric analysis of maturated DCs (CD80^+^CD86^+^CD11c^+^) (a) and cytotoxic T cells (CD3 + CD8+) (b) in the primary tumors after different treatments. c–e) Populations of maturated DCs (c), cytotoxic T cells (d), and helper T cells (e) (CD3^+^CD4^+^) in the primary tumors after different treatments (*n* = 3). f) Representative images of immunostaining of CD8 and CD4 in the primary tumors 2 weeks after different treatments. Scale bar: 50 μm. g–i) Secretion of TNF‐*α* (g), IFN‐*γ* (h), and IL‐1*β* (i) in serum measured by ELISA assay (*n* = 4). **p < 0.01 (one way ANOVA). j) Photographs and k) weights of the distant tumors (*n* = 5). Data is mean ± SD (*n* = 3 or 5). **p < 0.01, ***p < 0.001 determined by Student's *t*‐test.

Infiltrating immune cells were then analyzed to assess the immune response in vivo. The maturation of dendritic cells (DCs) was pivotal for initiating antitumor immunotherapy,^[^ ^63^ ^]^ which we investigated via flow cytometry. As shown in **Figure** **8**a,c, and Figure S42a and S43a, Supporting Information, CD80^+^CD86^+^ DCs in both tumors and draining lymph nodes of the treatment group were significantly upregulated compared with other control groups (group i and iii), suggesting that PyPS‐NF‐mediated photoimmunotherapy successfully promoted DC maturation. Furthermore, the DC‐mediated immune response was further studied by profiling intratumoral infiltration of cytotoxic T cells (CD8^+^) and helper T cells (CD4^+^), which play a key role in cancer immunotherapy.^[^ ^64^, ^65^ ^]^ Compared with the control groups, the populations of CD3^+^CD8^+^ and CD3^+^CD4^+^ T cells in the tumors of the treatment group were obviously higher (Figure 8b,d,e, S42b and S43b, Supporting Information). The results of immunostaining further indicated that the infiltration of cytotoxic and helper T cells in the tumor tissues of the treatment group was increased remarkably (Figure 8f). These findings suggested that PyPS‐NF‐mediated PDT successfully recruited cytotoxic and helper T cells to activate the immune response. In addition, cytokine profiling in serum was investigated via ELISA. As shown in Figure 8g–i, TNF‐*α*, interferon *γ* (IFN‐*γ*), and interleukin 1*β* (IL‐1*β*) were significantly increased after PyPS‐NF/light treatment, suggesting a pronounced immune activation. Significant immunotherapeutic outcome induced by this pyroptosis‐mediated immune activation was further demonstrated by the efficient suppression of distant tumors (Figure 8j,k). Taken together, the above results adequately demonstrated the promise of PyPS‐NF in precise and efficient immunotherapy against solid tumors.

## 3 Conclusion

We have designed and synthesized three photosensitizers (PyPS‐1, PyPS‐2, and PyPS‐3), which showed excellent photosensitivity in producing ROS under both normoxic and hypoxic conditions. In addition, they all demonstrated unexpected ER‐targeting capabilities without chemical conjugation of any ER‐targeting moieties. This specific photodamage of ER thereby initiated rapid and strong cell pyroptosis. We successfully revealed the mechanism of GSDME‐mediated pyroptosis by studying the best‐performing PyPS‐1. Finally, we established a general strategy to prepare activatable PyAs based on the caging/uncaging chemistry of the phenolic hydroxyl group of PyPS‐1, and thus successfully obtained a set of activatable PyPSs with specific responsiveness to biological species. As a proof‐of‐concept example, NTR‐activatable PyPS‐NF targeting tumor hypoxia was selected and demonstrated significant performances in precisely inducing immunogenic pyroptosis of hypoxic tumor cells in vitro and in vivo. This work establishes a unified design to prepare activatable PyAs, opening an avenue to tailor and synthesize activatable PyAs capable of inducing pyroptosis in a controlled manner, which is essential for advancing pyroptosis‐inducing cancer immunotherapy.

## 4 Experimental Section

4.1

4.1.1

##### Cell Culture

Cell lines including MCF‐7, A549, HepG2, A375, HeLa, NIH‐3T3, 293 T, and 4T1 cells were purchased from American Type Culture Collection (ATCC).

##### Tumor Model

All animal experiments were approved by the Animal Ethics Committee of Nankai University (2021‐SYDWLL‐000234). BALB/c female (6–8 weeks) were purchased from Beijing Vital River Laboratory Animal Technology Co., Ltd. (Beijing, China). For the subcutaneous tumor model, 4T1 cells (≈1 × 10^6^) suspended in 100 μL of PBS were subcutaneously injected into the right flank of each mouse. After about 10 days, the tumor‐bearing mice were then used subsequently.

##### Statistical Analysis

The data herein were given as means ± standard deviation. Statistical significance was determined using Student's *t*‐test or analysis of variance (ANOVA) analysis, and *p* < 0.05 was considered to be statistically significant (*, **, and *** indicate *p* < 0.05, *p* < 0.01, and *p* < 0.001, respectively).

## Conflict of Interest

The authors declare no conflict of interest.

## Supporting information

Supplementary Material

## Data Availability

The data that support the findings of this study are available from the corresponding author upon reasonable request.

## References

[1] J. Shi , Y. Zhao , K. Wang , X. Shi , Y. Wang , H. Huang , Y. Zhuang , T. Cai , F. Wang , F. Shao , Nature 2015, 526, 660. 26375003 10.1038/nature15514

[2] L. Galluzzi , A. Buque , O. Kepp , L. Zitvogel , G. Kroemer , Nat. Rev. Immunol. 2017, 17, 97. 27748397 10.1038/nri.2016.107

[3] X. Liu , S. Xia , Z. Zhang , H. Wu , J. Lieberman , Nat. Rev. Drug Discovery 2021, 20, 384. 33692549 10.1038/s41573-021-00154-z PMC7944254

[4] C. Rogers , D. A. Erkes , A. Nardone , A. E. Aplin , T. Fernandes-Alnemri , E. S. Alnemri , Nat. Commun. 2019, 10, 1689. 30976076 10.1038/s41467-019-09397-2 PMC6459836

[5] C. Rogers , T. Fernandes-Alnemri , L. Mayes , D. Alnemri , G. Cingolani , E. S. Alnemri , Nat. Commun. 2017, 8, 14128. 28045099 10.1038/ncomms14128 PMC5216131

[6] X. Wei , F. Xie , X. Zhou , Y. Wu , H. Yan , T. Liu , J. Huang , F. Wang , F. Zhou , L. Zhang , Cell Mol. Immunol. 2022, 19, 971. 35970871 10.1038/s41423-022-00905-x PMC9376585

[7] Z. Zhang , Y. Zhang , S. Xia , Q. Kong , S. Li , X. Liu , C. Junqueira , K. F. Meza-Sosa , T. M. Y. Mok , J. Ansara , S. Sengupta , Y. Yao , H. Wu , J. Lieberman , Nature 2020, 579, 415. 32188940 10.1038/s41586-020-2071-9 PMC7123794

[8] Y. Wang , W. Gao , X. Shi , J. Ding , W. Liu , H. He , K. Wang , F. Shao , Nature 2017, 547, 99. 28459430 10.1038/nature22393

[9] Z. Zhang , Y. Zhou , S. Zhao , L. Ding , B. Chen , Y. Chen , Adv. Sci. 2022, 9, 2203583. 10.1002/advs.202203583 PMC9762308 36266982

[10] P. Broz , P. Pelegrín , F. Shao , Nat. Rev. Immunol. 2020, 20, 143. 31690840 10.1038/s41577-019-0228-2

[11] M. Wu , X. Liu , H. Chen , Y. Duan , J. Liu , Y. Pan , B. Liu , Angew. Chem., Int. Ed. 2021, 60, 9093. 10.1002/anie.202016399 33543534

[12] P. Ji , S. Zhang , P. Liu , X. Li , W. Bao , X. Cui , M. Huo , J. Shi , Nano Today 2022, 44, 101511.

[13] L. Yu , Y. Xu , Z. Pu , H. Kang , M. Li , J. L. Sessler , J. S. Kim , J. Am. Chem. Soc. 2022, 144, 11326. 35708298 10.1021/jacs.2c03256

[14] W. Jiang , W. Dong , M. Li , Z. Guo , Q. Wang , Y. Liu , Y. Bi , H. Zhou , Y. Wang , ACS Nano 2022, 16, 3881. 35238549 10.1021/acsnano.1c09048

[15] M. Li , J. Kim , H. Rha , S. Son , M. S. Levine , Y. Xu , J. L. Sessler , J. S. Kim , J. Am. Chem. Soc. 2023, 145, 6007. 36881923 10.1021/jacs.3c01231 PMC12962054

[16] W. Piao , K. Hanaoka , T. Fujisawa , S. Takeuchi , T. Komatsu , T. Ueno , T. Terai , T. Tahara , T. Nagano , Y. Urano , J. Am. Chem. Soc. 2017, 139, 13713. 28872304 10.1021/jacs.7b05019

[17] M.-Y. Liang , M.-J. Zhang , W. Qiu , Y. Xiao , M.-J. Ye , P. Xue , Y.-J. Kang , Z.-J. Sun , Z. Xu , Adv. Sci. 2022, 9, 2203353. 10.1002/advs.202203353 PMC9475545 35869614

[18] B. Chen , Y. Yan , Y. Yang , G. Cao , X. Wang , Y. Wang , F. Wan , Q. Yin , Z. Wang , Y. Li , L. Wang , B. Xu , F. You , Q. Zhang , Y. Wang , Nat. Nanotechnol. 2022, 17, 788. 35606443 10.1038/s41565-022-01125-0

[19] P. Lu , X. Liu , X. Chu , F. Wang , J.-H. Jiang , Chem. Sci. 2023, 14, 2562. 36908949 10.1039/d2sc07044h PMC9993848

[20] V. N. Nguyen , S. Qi , S. Kim , N. Kwon , G. Kim , Y. Yim , S. Park , J. Yoon , J. Am. Chem. Soc. 2019, 141, 16243. 31577431 10.1021/jacs.9b09220

[21] K. M. Farrell , M. M. Brister , M. Pittelkow , T. I. Sølling , C. E. Crespo-Hernandez , J. Am. Chem. Soc. 2018, 140, 11214. 30145892 10.1021/jacs.8b07665

[22] S. Mai , M. Pollum , L. Martinez-Fernandez , N. Dunn , P. Marquetand , I. Corral , C. E. Crespo-Hernandez , L. Gonzalez , Nat. Commun. 2016, 7, 13077. 27703148 10.1038/ncomms13077 PMC5059480

[23] L. A. Ortiz-Rodriguez , S. J. Hoehn , A. Loredo , L. Wang , H. Xiao , C. E. Crespo-Hernandez , J. Am. Chem. Soc. 2021, 143, 2676. 33587618 10.1021/jacs.0c13203 PMC7985834

[24] K. Gu , Y. Xu , H. Li , Z. Guo , S. Zhu , S. Zhu , P. Shi , T. D. James , H. Tian , W. Zhu , J. Am. Chem. Soc. 2016, 138, 5334. 27054782 10.1021/jacs.6b01705

[25] X. Wang , P. Li , Q. Ding , C. Wu , W. Zhang , B. Tang , J. Am. Chem. Soc. 2019, 141, 2061. 30638380 10.1021/jacs.8b11414

[26] W. Zhai , Y. Zhang , M. Liu , H. Zhang , J. Zhang , C. Li , Angew. Chem., Int. Ed. 2019, 58, 16601. 10.1002/anie.201907510 31469219

[27] M. Liu , W. Zhai , H. Chen , H. Zhang , C. Li , Anal. Chem. 2020, 92, 10792. 32648733 10.1021/acs.analchem.0c02247

[28] M. Su , S. Li , H. Zhang , J. Zhang , H. Chen , C. Li , J. Am. Chem. Soc. 2019, 141, 402. 30547576 10.1021/jacs.8b10396

[29] Y. Yamakoshi , N. Umezawa , A. Ryu , K. Arakane , N. Miyata , Y. Goda , T. Masumizu , T. Nagano , J. Am. Chem. Soc. 2003, 125, 12803. 14558828 10.1021/ja0355574

[30] M. Li , Y. Shao , J. H. Kim , Z. Pu , X. Zhao , H. Huang , T. Xiong , Y. Kang , G. Li , K. Shao , J. Fan , J. W. Foley , J. S. Kim , X. Peng , J. Am. Chem. Soc. 2020, 142, 5380. 32105455 10.1021/jacs.0c00734

[31] X. Li , D. Lee , J. D. Huang , J. Yoon , Angew. Chem., Int. Ed. 2018, 57, 9885. 10.1002/anie.201806551 29927036

[32] R. J. Abraham , M. A. Warne , L. Griffiths , J. Chem. Soc., Perkin Trans. 1997, 2, 2151.

[33] H. Uoyama , K. Goushi , K. Shizu , H. Nomura , C. Adachi , Nature 2012, 492, 234. 23235877 10.1038/nature11687

[34] Z. Zhuang , J. Dai , M. Yu , J. Li , P. Shen , R. Hu , X. Lou , X. Zhao , B. Z. Tang , Chem. Sci. 2020, 11, 3405. 34745515 10.1039/d0sc00785d PMC8515424

[35] Q. Li , C. Huang , L. Liu , R. Hu , J. Qu , Cytometry, Part A 2018, 93, 997. 10.1002/cyto.a.23596 30230226

[36] K. Chen , P. He , Z. Wang , B. Z. Tang , ACS Nano 2021, 15, 7735. 33856778 10.1021/acsnano.1c01577

[37] P. Zheng , B. Ding , G. Zhu , C. Li , J. Lin , Angew. Chem., Int. Ed. 2022, 61, e202204904. 10.1002/anie.202204904 35687022

[38] L. Pelkmans , T. Bürli , M. Zerial , A. Helenius , Cell 2004, 118, 767. 15369675 10.1016/j.cell.2004.09.003

[39] L. Pelkmans , J. Kartenbeck , A. Helenius , Nat. Cell Biol. 2001, 3, 473. 11331875 10.1038/35074539

[40] H. Wang , X. Rong , G. Zhao , Y. Zhou , Y. Xiao , D. Ma , X. Jin , Y. Wu , Y. Yan , H. Yang , Y. Zhou , M. Qian , C. Niu , X. Hu , D.-Q. Li , Q. Liu , Y. Wen , Y.-Z. Jiang , C. Zhao , Z.-M. Shao , Cell Metab. 2022, 34, 581. 35278352 10.1016/j.cmet.2022.02.010

[41] L. Wang , R. Guan , L. Xie , X. Liao , K. Xiong , T. W. Rees , Y. Chen , L. Ji , H. Chao , Angew. Chem., Int. Ed. 2021, 60, 4657. 10.1002/anie.202013987 33217194

[42] H. Ma , Y. Lu , Z. Huang , S. Long , J. Cao , Z. Zhang , X. Zhou , C. Shi , W. Sun , J. Du , J. Fan , X. Peng , J. Am. Chem. Soc. 2022, 144, 3477. 35076232 10.1021/jacs.1c11886

[43] P. Luo , Y. Zhang , J. Zhang , H. Zhang , C. Li , Adv. Healthcare Mater. 2022, 11, 2201611. 10.1002/adhm.202201611 36066089

[44] P. Yu , X. Zhang , N. Liu , L. Tang , C. Peng , X. Chen , Signal Transduction Targeted Ther. 2021, 6, 128. 10.1038/s41392-021-00507-5 PMC8005494 33776057

[45] C. Chen , X. Ni , S. Jia , Y. Liang , X. Wu , D. Kong , D. Ding , Adv. Mater. 2019, 31, 1904914. 10.1002/adma.201904914 31696981

[46] G. Gao , Y.-W. Jiang , W. Zhan , X. Liu , R. Tang , X. Sun , Y. Deng , L. Xu , G. Liang , J. Am. Chem. Soc. 2022, 144, 11897. 35731698 10.1021/jacs.2c05743

[47] S. Zeng , C. Chen , L. Zhang , X. Liu , M. Qian , H. Cui , J. Wang , Q. Chen , X. Peng , Bioact. Mater. 2023, 25, 580. 37056275 10.1016/j.bioactmat.2022.07.016 PMC10087757

[48] M. Chiba , M. Kamiya , K. Tsuda-Sakurai , Y. Fujisawa , H. Kosakamoto , R. Kojima , M. Miura , Y. Urano , ACS Cent. Sci. 2019, 5, 1676. 31660435 10.1021/acscentsci.9b00678 PMC6813548

[49] J. F. Lovell , T. W. Liu , J. Chen , G. Zheng , Chem. Rev. 2010, 110, 2839. 20104890 10.1021/cr900236h

[50] Y. Li , Y. Sun , J. Li , Q. Su , W. Yuan , Y. Dai , C. Han , Q. Wang , W. Feng , F. Li , J. Am. Chem. Soc. 2015, 137, 6407. 25923361 10.1021/jacs.5b04097

[51] Z. Li , X. Li , X. Gao , Y. Zhang , W. Shi , H. Ma , Anal. Chem. 2013, 85, 3926. 23506563 10.1021/ac400750r

[52] A. Chevalier , Y. Zhang , O. M. Khdour , J. B. Kaye , S. M. Hecht , J. Am. Chem. Soc. 2016, 138, 12009. 27571326 10.1021/jacs.6b06229

[53] H. Chen , X. He , M. Su , W. Zhai , H. Zhang , C. Li , J. Am. Chem. Soc. 2017, 139, 10157. 28654288 10.1021/jacs.7b05920

[54] J. Ouyang , L. Sun , Z. Zeng , C. Zeng , F. Zeng , S. Wu , Angew. Chem., Int. Ed. 2020, 59, 10111. 10.1002/anie.201913149 31733015

[55] S. H. Gardner , C. J. Brady , C. Keeton , A. K. Yadav , S. C. Mallojjala , M. Y. Lucero , S. Su , Z. Yu , J. S. Hirschi , L. M. Mirica , J. Chan , Angew. Chem., Int. Ed. 2021, 60, 18860. 10.1002/anie.202105905 PMC8550804 34089556

[56] J. Fan , R.-H. Deng , H. Wang , X.-H. Liu , X.-N. Wang , R. Qin , X. Jin , T.-R. Lei , D. Zheng , P.-H. Zhou , Y. Sun , X.-Z. Zhang , Nano Lett. 2019, 19, 8049. 31558023 10.1021/acs.nanolett.9b03245

[57] J. Liu , S. He , Y. Luo , Y. Zhang , X. Du , C. Xu , K. Pu , J. Wang , Adv. Mater. 2022, 34, 2106654. 10.1002/adma.202106654 34854147

[58] A. Volchuk , A. Ye , L. Chi , B. E. Steinberg , N. M. Goldenberg , Nat. Commun. 2020, 11, 4561. 32917873 10.1038/s41467-020-18443-3 PMC7486936

[59] P. Agostinis , K. Berg , K. A. Cengel , T. H. Foster , A. W. Girotti , S. O. Gollnick , S. M. Hahn , M. R. Hamblin , A. Juzeniene , D. Kessel , M. Korbelik , J. Moan , P. Mroz , D. Nowis , J. Piette , B. C. Wilson , J. Golab , CA-Cancer J. Clin. 2011, 61, 250. 21617154 10.3322/caac.20114 PMC3209659

[60] L. Brancaleon , H. Moseley , Lasers Med. Sci. 2002, 17, 173. 12181632 10.1007/s101030200027

[61] L. Liu , C. Li , J. Gong , Y. Zhang , W. Ji , L. Feng , G. Jiang , J. Wang , B. Z. Tang , Angew. Chem., Int. Ed. 2023, 135, e202307776. 10.1002/anie.202307776 37358791

[62] X. Liu , W. Zhan , G. Gao , Q. Jiang , X. Zhang , H. Zhang , X. Sun , W. Han , F.-G. Wu , G. Liang , J. Am. Chem. Soc. 2023, 145, 7918. 36987560 10.1021/jacs.2c13189

[63] X. Su , W.-J. Wang , Q. Cao , H. Zhang , B. Liu , Y. Ling , X. Zhou , Z.-W. Mao , Angew. Chem., Int. Ed. 2022, 61, e202115800. 10.1002/anie.202115800 34842317

[64] T. Wang , D. Wang , H. Yu , B. Feng , F. Zhou , H. Zhang , L. Zhou , S. Jiao , Y. Li , Nat. Commun. 2018, 9, 1532. 29670088 10.1038/s41467-018-03915-4 PMC5906566

[65] H. Deng , W. Yang , Z. Zhou , R. Tian , L. Lin , Y. Ma , J. Song , X. Chen , Nat. Commun. 2020, 11, 4951. 33009382 10.1038/s41467-020-18745-6 PMC7532538

