# Supplementary material for: Conditionally Activatable Pyroptosis‐Inducing Agents for Cancer Therapy
Source: Small Sci. 2023 Dec 15;4(2):2300135. doi: 10.1002/smsc.202300135 (PMC11935172; doi:10.1002/smsc.202300135)
Supplement: Supplementary file 1 — Supplementary Material [file SMSC-4-2300135-s001.pdf]

Supporting Information

Conditionally Activatable Pyroptosis-Inducing Agents for Cancer Therapy

*Yongkang Zhang, Yaming Wang, Hao Zhang, Fan Qi, Eryan Li, and Changhua Li**
E-mail: chli@nankai.edu.cn (C.L.)

1. Materials and instruments

Materials: Unless otherwise noted, reagents were used as received from commercial sources. Solvent preparations were carried according to described procedures[1]. Trypsin-EDTA (0.25%) and 3-(4,5-dimethylthiazol-2-yl)-2,5-diphenyl-tetrazolium bromide (MTT) were purchased from Invitrogen. Dulbecco's Modified Essential Medium (DMEM), 1:1 mixture of Dulbecco's Modified Eagle's Medium and Ham's F-12 Nutrient Mixture (DME/F-12 1:1), fetal bovine serum (FBS), and 1 × phosphate buffer saline (PBS; pH 7.4) were purchased from Gibco (Life Technologies, AG, Switzerland). DNA from calf thymus (DNA-*ct*), 2′,7′-dichlorfluorescein-diacetate (DCF-DA), 1,3-diphenylisobenzofuran (DPBF), methylene blue (MB), 9,10-anthracenediyl-bis(methylene)dimalonic acid (ABDA), dihydroethidium (DHE), 5,5-dimethyl-1-pyrroline N-oxide (DMPO), JC-1, Fluo-3 AM, reduced nicotinamide adenine dinucleotide salt (NADH), and nitroreductase (NTR) were purchased from Sigma-Aldrich. Details of all antibodies used are as follows: GSDME (Abcam ab215191 and ab222407), CRT (Abclonal A18013), HMGB-1 (Abclonal A0719), caspase-3 (Abclonal A19654), *β*-Actin (Abclonal AC006), PE anti-mouse CD11c (Biolegend 117307), APC anti-mouse CD80 (Biolegend 104713), FITC anti-mouse CD86 (Biolegend 105109), PE anti-mouse CD3 (Biolegend 100205), APC anti-mouse CD8a (Biolegend 100711), FITC anti-mouse CD4 (Biolegend 100509). Water was deionized with a Milli-Q SP reagent water system (Millipore) to a specific resistivity of 18.2 MΩcm. Compound **3**,[2] **4**,[2] **5**[3], and QDPBF[4] were synthesized according to literature procedures.

Instruments: ^{1}H and ^{13}C NMR spectra were recorded at 25 °C on a Bruker AV400 NMR spectrometer, operating at 400 and 100 MHz, respectively, where chemical shifts (δ in ppm) were determined using partially or non-deuterated solvent residues as internal references. DMSO-d_6 and $CDCl_3$ were used as the solvents. High-resolution mass spectra (HRMS) were obtained on Varian 7.0T FTMS. HPLC analysis were performed with a Shimadzu HPLC system, equipped with a LC-20AT binary pump, an SPD-20A UV-vis detector, and a Symmetry C18 column. UV-vis absorption spectra were recorded on a UH5300 double-beam UV-vis spectrophotometer (Hitachi). Fluorescence spectra were recorded on an F-4600 (Hitachi) spectrofluorometer. MTT assay were monitored by the microplate reader (SpectraMax i3x, MD). Confocal laser scanning microscopy (CLSM) images were acquired using Leica TCS SP8 microscope. Electron paramagnetic resonance (EPR) spectra were recorded on a Bruker EMXplus-6/1 EPR spectrometer. Microscale thermophoresis (MST) was conducted by a Monolith NT.115 instrument. Cell phase contrast and fluorescence images were obtained by an inverted fluorescence microscope (ZOE Fluorescent Cell Imager, BIO-RAD). Flow cytometry analysis was conducted on

BD LSRFortessa. *In vivo* fluorescence imaging of tumor-bearing mice was conducted on NightOWL II LB983 In Vivo Imaging System (Berthold Technologies).

2. Chemical Synthesis

2.1 Synthesis of Compound 2

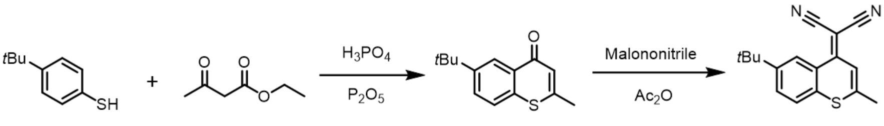

1 2

Compound 1: A mixture of phosphoric acid (20.7 g,180 mmol, 85%) and P_2O_5 (31.95 g, 225 mmol) was stirred at 90 °C for 1 h under nitrogen, followed by the addition of the mixture of 4-*tert*-butylthiophenol (4.3 g, 26.1 mmol) and ethyl acetoacetate (3.4 g, 26.1 mmol) over 15 min. After stirring for 4 h, the mixture was poured into ice/water (500 mL), then extracted three times with ethyl acetate (EtOAc; 200 mL). The combined organic layer was washed by 2% NaOH aqueous solution, dried over sodium sulfate, and evaporated to dryness. The residue was chromatographed on silica gel with PE/EtOAc (5/1 v/v) as an eluent to give compound **1** as yellow oil (3.09 g, 51.1%). ^{1}H NMR (400 MHz, $CDCl_3$, Figure S44) δ (ppm) 8.52 (d, J = 2.1 Hz, 1H), 7.65 (dd, J = 8.5, 2.1 Hz, 1H), 7.50 (d, J = 8.5 Hz, 1H), 6.84 (s, 1H), 2.45 (s, 3H), 1.38 (s, 9H). ^{13}C NMR (100 MHz, $CDCl_3$, Figure S45) δ (ppm) 180.84, 151.04, 150.92, 134.73, 130.38, 129.34, 125.78, 124.84, 124.65, 35.03, 31.21, 23.30.

Compound 2: Compound **1** (2.32 g, 10 mmol) and malononitrile (0.79 g, 12 mmol) were dissolved in 15 mL acetic anhydride, and the resulting mixture was refluxed for 2 h. The solvent was evaporated to dryness, and the residue was chromatographed on silica gel with PE/CH_2Cl_2 (1/1 v/v) as an eluent to give compound **2** as a yellow solid (1.21 g, 43.2%). ^{1}H NMR (400 MHz, $CDCl_3$, Figure S46) δ (ppm) 8.96 (d, J = 2.0 Hz, 1H), 7.69 (dd, J = 8.5, 2.0 Hz, 1H), 7.58 (d, J = 8.5 Hz, 1H), 7.41 (d, J = 1.2 Hz, 1H), 2.52 (d, J = 1.2 Hz, 3H), 1.41 (s, 9H). ^{13}C NMR (100 MHz, $CDCl_3$, Figure S47) δ (ppm) 156.53, 152.26, 148.81, 132.94, 129.98, 126.68, 125.21, 124.62, 120.58, 117.38, 116.08, 77.38, 77.06, 76.74, 67.35, 35.70, 31.09, 23.51. HRMS (ESI, Figure S66): m/z $[M+H]^+$ calcd. for $C_{17}H_{16}N_2S^+$ 281.1107; found 281.1109.

2.2 Synthesis of PyPS-1, PyPS-2, and PyPS-3

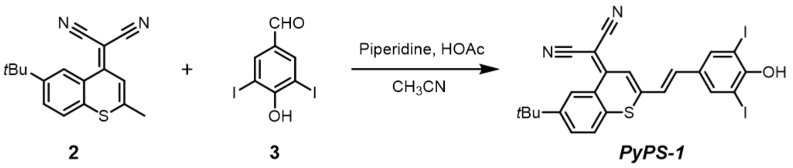

2 3 *PyPS-1*

PyPS-1: To a solution of compound **3** (1.12 g, 3.0 mmol) in dry acetonitrile (CH_3CN, 15 mL) was added piperidine (1.4 mL) and acetic acid (HOAc, 0.7 mL). The mixture was stirred for 10 min at room temperature before the addition of compound **2** (0.7 g, 2.5 mmol). The resulting mixture was stirred at reflux under nitrogen overnight, and dry molecular sieves were used to remove the produced water. After filtration and removing the solvent, the residue was dissolved in EtOAc, washed with HCl (1 M) and saturated brine, dried over sodium sulfate, and evaporated to dryness. The residue was chromatographed on silica gel with CH_2Cl_2/PE (1/1 v/v) as an eluent to afford **PyPS-1** as a red solid (1.02 g, 64.1%).

^{1}H NMR (400 MHz, DMSO-d_6, Figure S48) δ (ppm) 10.01 (s, 1H), 8.80 (s, 1H), 8.22 (s, 2H), 8.00–7.83 (m, 2H), 7.71 (d, J = 16.2 Hz, 1H), 7.64 (s, 1H), 7.29 (d, J = 16.2 Hz, 1H), 1.36 (s, 9H). ^{13}C NMR (100 MHz, DMSO-d_6, Figure S49) δ (ppm) 157.22, 156.14, 151.73, 148.43, 139.10, 134.11, 131.95, 131.77, 130.95, 128.08, 125.81, 124.60, 124.55, 121.58, 118.12, 116.43, 87.52, 66.59, 35.77, 31.13. HRMS (ESI, Figure S67): m/z $[M+H]^+$ calcd. for $C_{24}H_{18}I_2N_2OS^+$ 636.9302; found 636.9291.

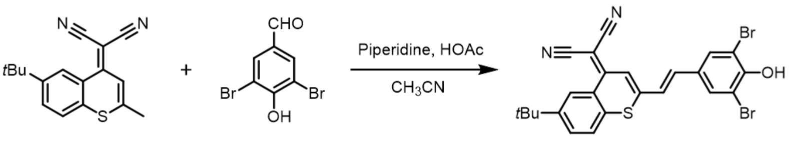

2 4 *PyPS-2*

PyPS-2: PyPS-2 was synthesized according to the same procedure as that used for **PyPS-1**. ^{1}H NMR (400 MHz, DMSO-d_6, Figure S50) δ (ppm) 10.49 (s, 1H), 8.80 (s, 1H), 8.05 (s, 2H), 7.91 (m, 2H), 7.76 (d, J = 16.2 Hz, 1H), 7.63 (d, J = 2.2 Hz, 1H), 7.30 (d, J = 16.2 Hz, 1H), 1.36 (s, 9H). ^{13}C NMR (100 MHz, DMSO-d_6, Figure S51) δ (ppm) 152.45, 151.80, 148.31, 134.37, 132.23, 131.95, 131.09, 130.24, 128.15, 126.27, 124.63, 124.56, 121.78, 118.11, 116.43, 112.66, 35.80, 31.11. HRMS (ESI, Figure S68): m/z $[M+H]^+$ calcd. for $C_{24}H_{18}Br_2N_2OS^+$ 542.9559; found 542.9547.

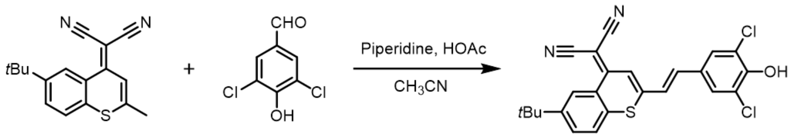

2 5 *PyPS-3*

PyPS-3: PyPS-3 was synthesized according to the same procedure as that used for **PyPS-1**.^{1}H NMR (400 MHz, DMSO-d_6, Figure S52) δ 10.67 (s, 1H), 8.80 (s, 1H), 7.94 – 7.83 (m, 4H), 7.72 (d, J = 16.3, 5.8 Hz, 1H), 7.61 (d, J = 5.8 Hz, 1H), 7.27 (d, J = 16.3, 5.8 Hz, 1H), 1.37 (s, 9H). ^{13}C NMR (100 MHz, DMSO-d_6, Figure S53) δ (ppm) 155.94, 151.78, 150.81, 148.15, 134.48, 131.90, 130.94, 128.66, 128.46, 128.04, 126.15, 124.52, 123.05, 121.73, 118.04, 116.38, 66.62, 35.76, 31.10. HRMS (ESI, Figure S69): m/z $[M+H]^+$ calcd for $C_{24}H_{18}Cl_2N_2OS^+$ 453.0590, found 453.0586

2.4 Synthesis of ^{1}PS-NTR

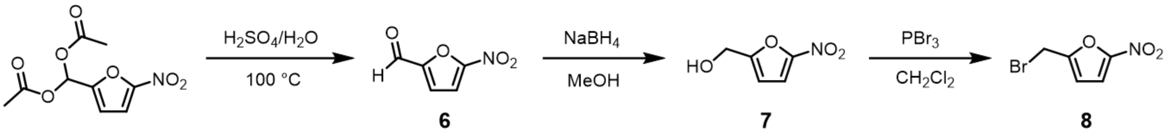

6 7 8

Compound 6: Compound **6** was synthesized according to a reported procedure.[5] 5-Nitrofurfural diacetate (15.00 g, 61.68 mmol) was dissolved in 120 mL sulphuric acid (50% v/v) and was stirred at 100 °C for 30 min. The resulting mixture was cooled to room temperature and extracted with EtOAc (3 × 100 mL). The combined organic layer was washed with water and saturated brine, dried over sodium sulfate, and evaporated to dryness. The residue was chromatographed on silica gel with PE/EtOAc (2/1 v/v) as an eluent to afford compound **6** as yellow oil (7.10 g, 81.6%).

Compound 7: Compound **7** was synthesized according to a reported procedure.[6] Compound **6** (5.00 g, 35.44 mmol) was dissolved in methanol (50 mL) and cooled to 0 °C. $NaBH_4$ (1.61 g, 42.53 mmol) was added to the reaction mixture

and stirred for 30 min. The resulting mixture was extracted with EtOAc (3 x 60 mL). The combined organic layer was washed with saturated brine, dried over sodium sulfate, and evaporated to dryness. The residue was chromatographed on silica gel with PE/EtOAc (1/2 v/v) as an eluent to afford compound **7** as yellow oil (4.17 g ,82.3%).

Compound 8: Compound **8** was synthesized according to a reported procedure.[6] Compound **7** (3.00 g, 20.96 mmol) was dissolved in CH_2Cl_2 (30 mL) and cooled to 0 °C. Phosphorus tribromide (2.36 mL, 25.15 mmol) in CH_2Cl_2 (10 mL) was added slowly at 0 °C, and the resulting mixture was stirred at room temperature for 1 h. After the reaction was completed, the mixture was poured into ice/water (200 mL), then extracted with CH_2Cl_2 (200 mL). The combined organic layer was washed with saturated sodium bicarbonate solution and saturated brine, dried over sodium sulfate, and evaporated to dryness. The residue was chromatographed on silica gel with PE/ EtOAc (3/1 v/v) as an eluent to afford compound **8** as a red solid (3.43 g, 79.4%).

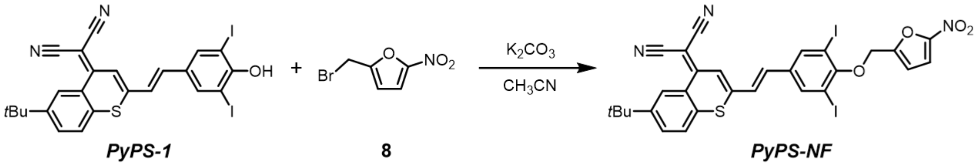

PyPS-NF: To a stirred solution of **PyPS-1** (300 mg, 0.47 mmol) in CH_3CN (10 mL) was added compound **8** (194 mg, 0.94 mmol) and K_2CO_3 (130 mg, 0.94 mmol), and the resulting mixture was stirred at 85 °C for 3 h. After filtration, the filtrate was evaporated to dryness. The residue was chromatographed on silica gel with PE/CH_2Cl_2 (1/3 v/v) as an eluent to afford **PyPS-NF** as a red solid (260 mg, 72.7%). ^{1}H NMR (400 MHz, $CDCl_3$, Figure S54) δ (ppm) 8.93 (d, J = 1.9 Hz, 1H), 7.99 (s, 2H), 7.72 (dd, J = 8.5, 1.9 Hz, 1H), 7.63 (d, J = 8.5 Hz, 1H), 7.59 (s, 1H), 7.35 (d, J = 3.6 Hz, 1H), 7.06 (s, 2H), 6.81 (d, J = 3.6 Hz, 1H), 5.11 (s, 2H), 1.42 (s, 9H). ^{13}C NMR (100 MHz, $CDCl_3$, Figure S55) δ (ppm) 157.62, 156.11, 152.53, 152.47, 145.65, 138.81, 135.55, 132.18, 131.31, 130.34, 127.47, 127.16, 125.25, 125.09, 122.69, 116.99, 115.79, 113.61, 112.20, 91.39, 77.23, 66.27, 35.72, 31.08. HRMS (ESI, Figure S70): m/z $[M+Na]^+$ calcd. for $C_{29}H_{21}I_2N_3NaO_4S^+$ 783.9234; found 783.9238.

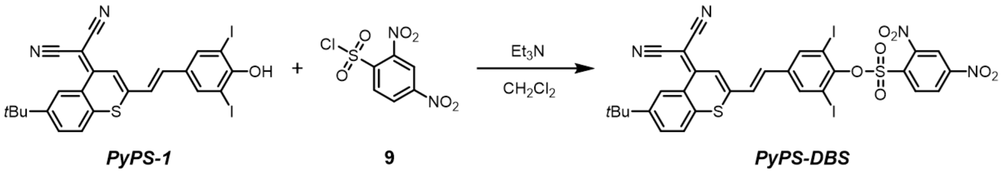

PyPS-DBS: To a stirred solution of **PyPS-1** (250 mg, 0.39 mmol) in dry CH_2Cl_2 (5 mL) was added Et_3N (0.78 mL). The mixture was then cooled to 0 °C and a solution of compound **9** (125 mg, 0.48 mmol) in dry CH_2Cl_2 (3 mL) was added dropwise. After being stirred at 0 °C for 30 min, the mixture was then stirred at room temperature for 3 h. After the completion of the reaction, the mixture was condensed under reduced pressure. The residue was purified with column chromatography (silica gel, CH_2Cl_2/MeOH, 100:1 v/v), and a red solid was obtained (231 mg, 68.3%). ^{1}H NMR (400 MHz, $CDCl_3$, Figure S56) δ (ppm) 8.93 (s, 1H), 8.78 (s, 1H), 8.62 (d, J = 9.1 Hz, 1H), 8.46 (d, J = 9.1 Hz, 1H), 8.02 (s, 2H), 7.72 (d, J = 8.5 Hz, 1H), 7.68 – 7.53 (m, 2H), 7.11 (d, J = 16.1 Hz, 1H), 7.03 (d, J = 16.1 Hz, 1H)., 1.41 (s, 9H).^{13}C NMR (100 MHz, $CDCl_3$, Figure S57) δ (ppm) 156.00, 152.67, 152.25, 145.06, 141.94, 139.32, 137.36, 133.78, 133.65,

131.30, 131.17, 131.03, 130.45, 129.01, 127.19, 126.88, 125.26, 125.04, 123.26, 120.80, 116.84, 115.66, 91.10, 77.23, 35.73, 31.07

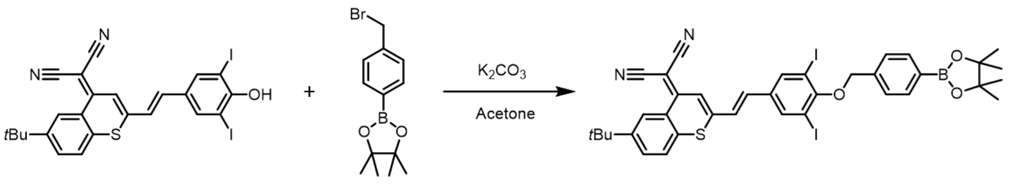

PyPS-1 **10** ***PyPS-PB***

PyPS-PB: To a solution of **PyPS-1** (318 mg, 0.50 mmol) and K_2CO_3 (207 mg,1.50 mmol) in dry acetone (10 mL), compound **10** (297 mg, 1.00 mmol) was added. The resulting mixture was stirred at 65 °C for 4 h. After filtration, the filtrate was evaporated to dryness. The residue was chromatographed on silica gel with PE/ CH_2Cl_2 (1/2 v/v) as an eluent, affording **PyPS-PB** as a red solid (306 mg, 72%). ^{1}H NMR (400 MHz, $CDCl_3$, Figure S58) δ (ppm) 8.93 (s, 1H), 7.99 (s, 2H), 7.88 (d, J = 7.3 Hz, 2H), 7.71 (d, J = 8.6 Hz, 1H), 7.64 (d, J = 7.3 Hz, 2H), 7.61 (d, J = 8.6 Hz, 1H), 7.58 (s, 1H), 7.05 (s, 2H), 5.06 (s, 2H), 1.41 (s, 9H), 1.36 (s, 12H).^{13}C NMR (100 MHz, $CDCl_3$, Figure S59) δ (ppm) 158.61, 156.07, 152.44, 145.89, 138.81, 138.74, 134.95, 134.86, 132.57, 131.36, 130.27, 127.50, 127.15, 126.89, 125.20, 125.09, 122.44, 117.05, 115.82, 91.72, 83.87, 74.58, 69.25, 35.70, 31.08, 24.90.

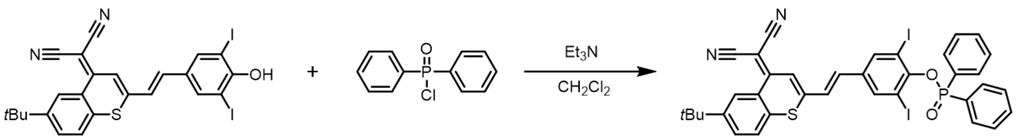

PyPS-1 **11** ***PyPS-SAR***

PyPS-SAR: To a stirred solution of **PyPS-1** (250 mg, 0.39 mmol) in dry CH_2Cl_2 (5 mL) was added Et_3N (0.78 mL). The mixture was then cooled to 0°C, and a solution of compound **11** (118 mg, 0.50 mmol) in dry CH_2Cl_2 (3 mL) was added dropwise. After being stirred at 0 °C for 30 min, the mixture was then stirred at room temperature for 1 h. After the completion of the reaction, the mixture was condensed under reduced pressure. The residue was purified with column chromatography (silica gel, CH_2Cl_2/MeOH, 100/1 v/v), and a red solid was obtained (248 mg, 76.1%).^{1}H NMR (400 MHz, $CDCl_3$, Figure S60) δ (ppm) 8.92 (s, 1H), 7.98 (s, 2H), 7.94 (m, 4H), 7.70 (s, 1H), 7.6 (m, 4H), 7.51 (m, 4H), 7.01(s, 2H), 1.41 (s, 9H). ^{13}C NMR (100 MHz, $CDCl_3$, Figure S61) δ (ppm) 156.13, 153.61, 152.44, 145.83, 139.23, 132.68, 132.66, 132.37, 132.26, 132.15, 130.27, 128.53, 128.39, 127.24, 127.14, 125.22, 122.54, 117.04, 115.79, 90.60, 69.33, 35.69, 31.0

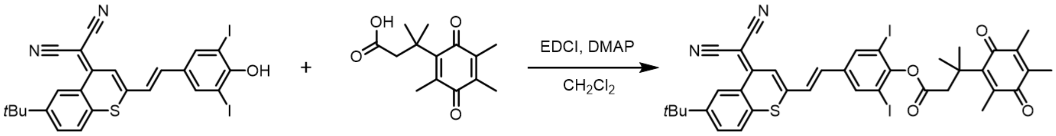

PyPS-1 **12** ***PyPS-BQ***

PyPS-BQ: To a mixture of **PyPS-1** (70 mg, 0.11 mmol) in dry CH_2Cl_2 were added EDCI (103 mg, 0.22 mmol) and 4-dimethyl aminopyridine (13 mg, 0.11 mmol) at 0 °C. After being stirred at 0 °C for 30 min, a solution of compound **12** (43 mg, 0.17 mmol) in dry CH_2Cl_2 (3 mL) was added dropwise. After the reaction solution was stirred at room temperature for 1 h, the mixture was condensed under reduced pressure. The residue was purified with column chromatography (silica

gel, PE/CH_2Cl_2 = 1/2 v/v), and a red solid was obtained (78 mg, 81.9%). ^{1}H NMR (400 MHz, $CDCl_3$, Figure S62) δ (ppm) 8.92 (s, 1H), 7.93 (s, 2H), 7.71 (d, J = 8.5 Hz, 1H), 7.62 (d, J = 8.5 Hz, 1H), 7.57 (s, 1H), 7.03 (s, 2H), 3.47 (s, 2H), 2.18 (s, 3H), 1.97 (s, 3H), 1.92 (s, 3H), 1.57 (s, 6H), 1.41 (s, 9H). ^{13}C NMR (100 MHz, $CDCl_3$, Figure S63) δ (ppm) 190.77, 187.36, 169.55, 156.06, 152.49, 151.99, 145.56, 142.84, 138.81, 138.64, 138.10, 136.20, 132.17, 131.28, 130.33, 127.66, 127.14, 125.21, 125.05, 122.76, 116.97, 115.76, 91.13, 69.52, 47.97, 37.89, 35.70, 31.07, 28.85, 14.43, 12.98, 12.16.

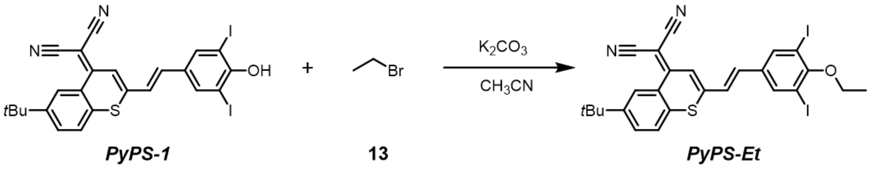

PyPS-Et: To a stirred solution of **PyPS-1** (270 mg, 0.42 mmol) was added compound **13** (69 mg, 0.64mmol) and K_2CO_3 (176 mg, 1.26 mmol) in CH_3CN, the mixture was stirred at 80 °C for 3 h. After the completion of the reaction, the mixture was condensed under reduced pressure. The residue was purified with column chromatography. ^{1}H NMR (400 MHz, $CDCl_3$, Figure S64) δ (ppm) 8.93 (s, 1H), 7.96 (s, 2H), 7.71 (d, J = 8.5, 1H), 7.62 (d, J = 8.5 Hz, 1H), 7.57 (s, 1H), 7.03 (m, 2H), 4.08 (q, J = 7.0 Hz, 2H), 1.55 (t, J = 6.3 Hz, 3H), 1.41 (s, 9H). ^{13}C NMR (100 MHz, $CDCl_3$, Figure S65) δ (ppm) 159.40, 156.18, 152.43, 146.00, 138.63, 134.52, 132.70, 131.39, 130.27, 127.13, 126.71, 125.23, 125.12, 122.39, 115.87, 91.65, 77.23, 69.71, 69.17, 35.70, 31.07, 15.52.

3. Studies of PyPSs and PyPS-CG in solution

3.1 Molar Absorption Coefficient (ε)

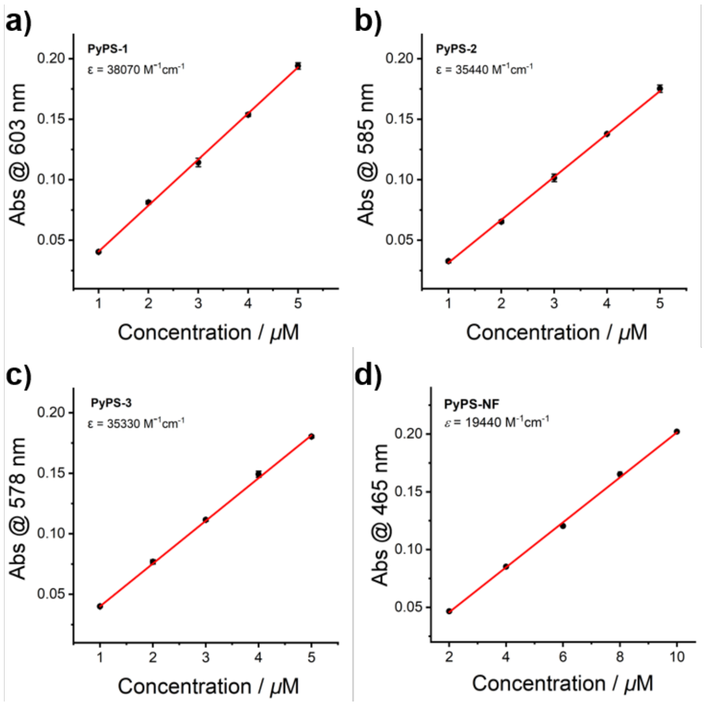

Figure S1. Calibration curves of absorbance versus concentration for (a) **PyPS-1**, (b) **PyPS-2**, (c) **PyPS-3**, and (d) **PyPS-NF** in PBS (pH 7.4). ε: molar absorption coefficient.

Taking the measurement of **PyPS-1** as an example. UV-vis absorption spectra of the aqueous solutions of **PyPS-1** in PBS (pH 7.4; 5% v/v DMF, 0.3% F127) with varying concentrations (1, 2, 3, 4 and 5 μM) were recorded. Measurements were carried out in 1 cm quartz cuvettes with a total sample volume of 3 mL. Molar absorption coefficient was obtained by fitting the curve of the absorbance at the maximum absorption wavelength and the concentration of **PyPS-1** (Figure S1a). The results represent the average of three independent experiments.

Molar absorption coefficients of other dyes (**PyPS-2**, **PyPS-3**, and **PyPS-NF**) were measured according to the same method (Figure S1b-d).

3.2 Fluorescence Quantum Yield (Φ_{fl})

According to a previous report,[7] Φ_{fl} of three **PyPS** molecules were measured using Rhodamine B (RhB) as a reference with Φ_{fl} of 0.97 in ethanol. The fluorescence quantum yield was calculated by using the following formula:

$$\Phi_X = \Phi_{ST}\left(\frac{Grad_X}{Grad_{ST}}\right)\left(\frac{\eta_X^2}{\eta_{ST}^2}\right) \qquad (1)$$

where Φ_X and Φ_{ST} are the fluorescence quantum yields of the sample and the standard (RhB), respectively. $Grad_X$ and $Grad_{ST}$ are the gradients from the plots of the integrated fluorescence intensity versus absorbance of the sample and RhB, respectively. The absorbance of the sample or RhB at the excitation wavelength was less than 0.1. η_X and η_{ST} are the refractive indices of the solvents used for the sample and RhB, respectively. Measurements were carried out in 1 cm quartz cuvettes with a total sample volume of 3 mL.

3.3 pK_a Determination

The pK_a values of three **PyPS** molecules were determined using the Henderson-Hasselbalch equation[8]:

$$\log[(A_{max} - A)/(A - A_{min})] = pK_a - pH \qquad (2)$$

where A_{max} and A_{min} represent maximum absorbance and minimal absorbance at the measured wavelength, respectively, A represents the observed absorbance. The pK_a values were then calculated based on the plots of $\log[(A_{max}-A)/(A-A_{min})]$ vs. pH.

3.4. Investigation of the Sensitivities of PyPS-CG Molecules

*Stability of **PyPS-NF*** UV-vis absorption spectrophotometer was utilized to evaluate the stability of **PyPS-NF**. The UV-vis absorption spectrum of **PyPS-NF** (5.0 μM) in PBS buffer solution was recorded over a period of 60 min.

*NTR-triggered **PyPS-NF** to* **PyPS-1** *conversion.* NTR (5 μg/mL) and NADH (0.5 mM) were added to the solution of **PyPS-NF** (5.0 μM) in PBS (pH 7.4), and incubated for varying time intervals (0-60 min) at 37 °C. UV-vis absorption spectrophotometer and HPLC were adopted to monitor NTR/NADH-induced **PyPS-NF** to **PyPS-1** conversion. HPLC was performed by using an isocratic elution program with a solvent system of methanol (5 mM ammonium formate) at a flow rate of 1.0 mL/min with detection wavelength at 365 nm.

*H_2O_2-triggered **PyPS-PB** to* **PyPS-1** *conversion.* H_2O_2 (100 μM) was added to the solution of **PyPS-PB** (5.0 μM) in PBS (pH 7.4) and incubated for 2 h at 37 °C. UV-vis absorption spectrophotometer was adopted to monitor H_2O_2-induced

conversion.

*$O_2^{\bullet-}$-triggered **PyPS-SAR** to **PyPS-1** conversion.* $O_2^{\bullet-}$ (100 μM) was added to the solution of **PyPS-SAR** (5.0 μM) in PBS (pH 7.4) and incubated for 30 min at 37 °C. UV-vis absorption spectrophotometer was adopted to monitor $O_2^{\bullet-}$-induced conversion. Potassium superoxide (KO_2) was used as superoxide anion radical ($O_2^{\bullet-}$) source.[9]

*GSH-triggered **PyPS-DBS** to **PyPS-1** conversion.* Glutathione (GSH, 2mM) was added to the solution of **PyPS-DBS** (5.0 μM) in citric acid/Na_2HPO_4 buffer solution (pH 7.4) and incubated for 2 h at 37 °C. UV-visible absorption spectrophotometer was adopted to monitor GSH-induced conversion.

*NQO1-triggered **PyPS-BQ** to **PyPS-1** conversion.* $Na_2S_2O_4$ (30 μM) as a NQO1 mimic[10] was add to the solution of **PyPS-BQ** (5.0 μM) in citric acid/Na_2HPO_4 buffer solution (pH 9) and incubated for 30 min at 37 °C. UV-visible absorption spectrophotometer was adopted to monitor $Na_2S_2O_4$-induced conversion.

4. Determination of the Photosensitized ROSs

4.1 ROS Detection with QDPBF

The total photogenerated ROSs by **PyPS** molecules or MB was measured using QDPBF as a probe. Typically, the stock aqueous solution of QDPBF was added to the PBS solution of photosensitizer to give a final aqueous test solution (2.5 μM). For the QDPBF content, it was fixed at a concentration where the absorbance at 415 nm is around 1.0. The obtained solutions were then placed in a cuvette and subjected to light irradiation (Xe lamp, 490-700 nm, 5 mW/cm^2) for 1 min. The change in the absorbance of QDPBF (ΔAbs) at 415 nm was recorded at 10-s intervals to evaluate the photosensitivity in producing ROS. $\Delta Abs = Abs_0 - Abs_t$; where Abs_0 and Abs_t are the absorbance of QDPBF at 415 nm before and after light irradiation, respectively.

4.2 Singlet Oxygen ($^1O_2$) Detection with ABDA

The $^1O_2$ generated by **PyPS** molecules or MB upon light irradiation was measured using ABDA as an indicator. Typically, the stock aqueous solution of ABDA was added to the PBS solution of photosensitizer to give a final aqueous test solution (2.5 μM). For the ABDA content, it was fixed at a concentration where the absorbance at 380 nm is around 1.0. The obtained solutions were then placed in a cuvette and subjected to light irradiation (Xe lamp, 490-700 nm, 5 mW/cm^2) for 10 min. The decrease in absorbance of ABDA (ΔAbs) at 380 nm was recorded at 2-min intervals to evaluate the photosensitivity in producing $^1O_2$. $\Delta Abs = Abs_0 - Abs_t$; where Abs_0 and Abs_t are the absorbance of ABDA at 380 nm before and after light irradiation, respectively.

4.3 Superoxide Anion Radical ($O_2^{\bullet-}$) Detection with DHE

The photogenerated $O_2^{\bullet-}$ by **PyPS** molecules was measured using DHE as a fluorescence probe with the aid of DNA-*ct*. Typically, the stock DMF solution of DHE and aqueous solution of DNA-*ct* were sequentially added to the PBS solution of **PyPSs** to give a final aqueous test solution (conc. 2.5 μM photosensitizer, 10 μM DHE, 40 μg/mL DNA-*ct*). The obtained solutions were then placed in a cuvette and subjected to light irradiation (Xe lamp, 490-700 nm, 5 mW/cm^2) for 10 min. The fluorescence emission spectra were recorded at 2-min intervals. The fluorescence intensity enhancement of DHE at 580 nm, *i.e.*, $(F_t - F_{blank})/F_0$, was calculated to evaluate the photosensitivity in producing $O_2^{\bullet-}$, where F_0 and F_t

represent the emission intensities of DHE at 580 nm in the presence of **PyPSs** before and after light irradiation, respectively, while F_{blank} represents the emission intensity of DHE at 580 nm without **PyPSs** after light irradiation.

4.4 Identification of the Photogenerated ROS via EPR Spectrometry

Electron paramagnetic resonance (EPR) spectra were recorded on a Bruker EMXplus 6/1 EPR spectrometer. DMPO was used as a special radical trapping agent for $O_2^{\bullet -}$. Typically, 100 mM DMPO was mixed with 100 μM photosensitizer in DMF, followed by light irradiation for 3 min. EPR was then performed at a microwave frequency of 9.8 GHz and power of 20 mW with a modulation amplitude and frequency of 1.0 G and 100 kHz, respectively, time constant 0.01 ms, scan time 30 s, receiver gain 30 dB, and center field setting 3512 G. The light source in EPR spectrometry is a red LED lamp (570-660 nm, 20 mW/cm^2).

5. Computational Details

Density functional theory (DFT) and time-dependent density function theory (TD-DFT) methods were carried out on Gaussian 09 program (Revision D.01) under the solvent model of Solvation Model Based on Density (SMD) for H_2O. The ground state geometries were optimized using the DFT at the B3LYP/SMD/def2TZVP level. The excitation energies in the singlet (S_n) and triplet (T_n) states were calculated using TD-DFT at the B3LYP/SMD/def2TZVP level. Moreover, the Gibbs free energies of the $O_2^{\bullet -}$ generation process by **PyPS-1** was calculated at the B3LYP/SMD/def2TZVP level.

Docking Calculations: The binding affinity calculation **PyPS-NF** and NTR was performed on AutoDock software (4.2.6 version).[11] The NTR structure was obtained from PDB under code 4DN2, two chains and total 208 amino acid residues. The docking results and figures were obtained on AutoDock, Pymol, and LigPlot+.

6. *In Vitro* Studies

6.1 Cell Culture

Cell culture under normoxic conditions (21% O_2). The cells were cultured in DMEM or DME/F-12 (for 4T1 cells only) supplemented with 10% heatinactivated FBS, penicillin (100 U/mL), and strepotomycin (100 μg/mL) in a 37 °C incubator with 5% CO_2. Before experiment, the cells were pre-cultured until confluence was reached to about 75%.

Cell culture under hypoxic conditions (2% O_2). To simulate the hypoxic tumor environment, tumor cells were incubated under hypoxia. Briefly, after incubation under normoxic conditions (21% O_2), the cells were transferred to a hypoxia incubator chamber (#27310, Stemcell Technologies), and incubated for additional 12 h at 37 °C in a humidified atmosphere (2% O_2, 5% CO_2, 93% N_2).

6.2 Cell Imaging of PyPS Molecules

*Cell uptake of **PyPS-1**.* Typically, MCF-7 cells were plated onto 35 mm confocal dishes and incubated at 37 °C for 24 h. Freshly prepared **PyPS-1** solution (1.0 μM, 0.01% F127) in DMEM was added and the cells were incubated for different time intervals (0.5, 1, 2, and 4 h). Then, the cells were washed with PBS (10 mM, pH 7.4) for three times, and imaged immediately by CLSM. Ex/Em: 594/680-800 nm.

*NTR-triggered **PyPS-NF** to **PyPS-1** Conversion within Hypoxic Cells.* MCF-7 cells were incubated in hypoxia as described in Section 6.1, then subjected to following different treatments: group 1, incubated with **PyPS-NF** (1 μM) under

normoxia for 6 h; group2, incubated with **PyPS-NF** (1.0 μM) under hypoxia for 6 h. After washing with PBS (10 mM, pH 7.4) for three times, the cells of different groups were imaged immediately by CLSM. Notably, for experiments in hypoxia, cell culture and treatments were conducted in a hypoxia incubator chamber, and all the DMEM used were bubbled with mixed gas (2% O_2, 5% CO_2, and 93% N_2) for 5 min in advance. Ex/Em: 594/680-800 nm.

Colocalization study. Typically, Mito-Tracker-Green FM (MTG) and ER-Tracker Green (ERTG) were utilized to specifically label mitochondria and endoplasmic reticulum, respectively, in living cells. Typically, cancer cells (MCF-7, HeLa, or HepG2 cells) were incubated with **PyPS** molecules (2.0 μM) for 1 h and washed twice with PBS. Then, the freshly prepared fluorescent tracker (MTG: 200 nM; ER-Tracker Green: 1.0 μM) in DMEM was added. The cells were incubated for another 30 min, then washed with PBS and imaged on a Leica TCS SP8 microscope. PyPSs channel: Ex/Em: 594 nm/600–800 nm; tracker channel: Ex/Em:488 nm/500–550 nm. Pearson's correlation coefficient (PCC) was calculated from 5 independent images.

6.3 Cellular uptake pathways.

Cellular uptake pathways of ***PyPS-1*** were investigated via endocytosis inhibitor assays, including genistein (GEN, an inhibitor of caveolae-mediated endocytosis; 200 μM), methyl-β-cyclodextrin (Mβ-CD, an inhibitor of lipid raft-mediated endocytosis; 2 mM), chlorpromazine (CPZ, an inhibitor of clathrin-mediated endocytosis; 10 μM), 5-(*N*-ethyl-*N*-isopropyl)-amiloride (EIPA, an inhibitor of micropinocytosis; 100 μM), and cytochalasin B (CytB, an inhibitor of phagocytosis and/or micropinocytosis; 100 μM). Typically, MCF-7 cells were incubated with different inhibitors for 1 h and washed with PBS. Then, **PyPS-1** in DMEM was added. The cells were incubated for another 0.5 h, then washed with PBS and imaged on a Leica TCS SP8 microscope. Ex/Em: 594 nm/600–800 nm.

6.4 Cytosolic Calcium imaging.

MCF-7 cells were plated onto 35 mm confocal dishes and incubated at 37 °C for 24 h. After incubation with **PyPS-1** (1.0 μM) for 2 h, the cells were washed with PBS for three times. Fluo-3 AM (5.0 μM) was added and stained for another 30 min, then washed with PBS. The cells were subjected to light irradiation (Xe lamp, 490-700 nm, 10 mW/cm^2) for 10 min and imaged immediately by CLSM. Ex/Em: 488/500-550 nm.

6.5 Mitochondrial Membrane Potentials Assay

Mitochondrial membrane potential ($\Delta\psi_m$) was estimated by using JC-1, a cationic dye that accumulates in mitochondria, as a fluorescent probe. Typically, MCF-7 cells were incubated with **PyPS-1** (1.0 μM) for 2 h, the cells were washed twice with PBS and subjected to light irradiation (Xe lamp, 490-700 nm, 10 mW/cm^2) for 5 min. After incubation for 10 min, the freshly prepared JC-1 probe solution (5.0 μg/mL) in DMEM was added and stained for 20 min. The cells were washed with PBS and imaged on a Leica TCS SP8 microscope. Notably, for experiments in hypoxia, cell culture and treatments were conducted in a hypoxia incubator chamber, and all the DMEM used were bubbled with mixed gas (2% O_2, 5% CO_2, and 93% N_2) for 5 min in advance. JC-1/aggregate channel: Ex/Em 543/560-600 nm; JC-1/monomer channel: Ex/Em 488/500-530 nm.

6.6 Photogenerated ROS by PyPS-1 in Normoxic or Hypoxic Cells

Evaluation of the photogenerated ROS with DCF-DA. Dichlorofluorescein diacetate (DCF-DA) was chosen as a general oxidant-sensitive fluorescent dye to detect the photogenerated ROS, which rapidly oxidized DCF-DA into highly fluorescent dichlorofluorescein (DCF). Typically, MCF-7 cells were incubated with 1.0 μM photosensitizer for 2 h (**PyPS-1**) or 6 h (**PyPS-NF**), and the cells were washed thrice with PBS. Freshly prepared DCF-DA solution (10 μM) in DMEM was added and the cells were incubated for 20 min. After washing with PBS, the cells were subjected to light irradiation (Xe lamp, 490-700 nm, 10 mW/cm^2) for 10 min and imaged immediately by CLSM. Ex/Em: 488/500-550 nm.

Evaluation of the photogenerated $O_2^{\bullet-}$ with DHE. DHE was used as a specific fluorescent dye to detect the generated $O_2^{\bullet-}$. Typically, MCF-7 cells were incubated with 1.0 μM photosensitizer for 2 h (**PyPS-1**) or 6 h (**PyPS-NF**), the cells were washed thrice with PBS, freshly prepared DHE solution (10 μM) in DMEM was added and the cells were incubated for 20 min. The cells were washed with PBS, then subjected to light irradiation (Xe lamp, 490-700 nm, 10 mW/cm^2) for 10 min, and imaged immediately by CLSM. Ex/Em: 561/570-630 nm.

Notably, for experiments in hypoxia, cell culture and treatments were conducted in a hypoxia incubator chamber, and all the DMEM used were bubbled with mixed gas (2% O_2, 5% CO_2, and 93% N_2) for 5 min in advance.

6.7 Flow Cytometry Analysis of Intracellular ROS Level and Cell Death Pathways

After incubation with **PyPS-NF** (1.0 μM) for 6 h, MCF-7 cells were washed with PBS thrice. Freshly prepared DCF-DA or DHE solution (10 μM) in DMEM was added and the cells were incubated for additional 30 min. The cells were then washed with PBS, followed by light irradiation (Xe lamp, 490-700 nm, 10 mW/cm^2) or incubation in dark for 10 min and collected by trypsin. After washed with PBS thrice, cells were resuspended in PBS to form a single-cell suspension. The intracellular ROS level was analyzed using flow cytometry (BD LSRFortessa). Notably, for experiments in hypoxia, cell culture and treatments were conducted in a hypoxia incubator chamber, and all the DMEM used were bubbled with mixed gas (2% O_2, 5% CO_2, and 93% N_2) for 5 min in advance.

After incubation with **PyPS-1** (1.0 μM) for 2 h, MCF-7 cells were washed with PBS, followed by light irradiation (Xe lamp, 490-700 nm, 10 mW/cm^2) or incubation in dark for 10 min. After incubation for 1 h and collected by trypsin. The cell death pathway was analyzed using flow cytometry (BD LSRFortessa).

6.8 Extracellular ATP Levels and LDH Release

MCF-7 cells were seeded in 96-well plates and incubated under normoxia or hypoxia for 24 h. Then, the cells were incubated with photosensitizers for 2 h (**PyPS-1**) or 6 h (**PyPS-NF**). After washing with PBS, the cells were subjected to light irradiation (Xe lamp, 490-700 nm, 10 mW/cm^2) for 10 min. For ATP measurement, the light-treated cells were incubated for 1 h at 37 °C and then 2 h at 4 °C, and the conditioned medium was transferred to a 96-well white bottom plate for the measurement of extracellular ATP via luciferase ATP Assay Kit. For LDH measurement, the light-treated cells were incubated for 3 h at 37 °C, and the conditioned medium was transferred to a 96-well plate for the detection of LDH release via LDH Assay Kit.

6.9 Intracellular NTR Levels

MCF-7 cells were seeded in 96-well plates and incubated under normoxia or hypoxia for 24 h. The cells were collected

and lysed through repeated freezing and thawing in water, followed by centrifuged. The supernatants were collected for the measurement of intracellular NTR levels via ELISA assay.

6.10 Western Blotting

MCF-7 cells were seeded in 35 mm dishes and incubated with different photosensitizers (**PyPS-1**: 2 h; **PyPS-NF**: 6 h) and subjected to light irradiation under normoxic or hypoxic conditions. After 3 h incubation, the cells were washed with cold PBS and treated with the ice-cold RIPA Lysis Buffer containing 100 μg/mL phenylmethanesulfonyl fluoride (PMSF) and 1% phosphatase inhibitor. Cell lysates were collected and kept on ice for 30 min. After centrifuging to remove insoluble matter, the supernatant was mixed with loading buffer and heated in a boiling water bath for 5 min. Protein samples were aliquoted and separated off by SDS-polyacrylamide gel electrophoresis. They were then transferred to polyvinylidene difluoride (PVDF) membranes. These PVDF membranes were dip in 5 % skim milk for 1h and incubated with the primary antibody overnight at 4 °C and further the secondary antibody for 1 h. The protein bands were detected using a ECL luminescence kit and imaged using a fully automatic multi-function imaging system.

6.11 Immunofluorescence Staining

MCF-7 cells were seeded in 35 mm dishes and incubated with different photosensitizers (**PyPS-1**: 2 h; **PyPS-NF**: 6 h), followed by light irradiation under normoxic or hypoxic conditions. After 3 h incubation, the cells were washed with sterile PBS and fixed by 4% paraformaldehyde for 15 min. The fixative was then washed off with PBS and replaced with a 0.1% triton X-100 for 15 min to permeabilize the cells (except when staining membrane proteins like CRT). The cells were then blocked with 2% BSA for 30 min and incubated with the primary antibody overnight at 4 °C and further Alexa Fluor 488 Goat Anti-Rabbit IgG for 1 h. After that, the cells were incubated with Hoechst 33342 for 15 min. After being washed with PBS, an anti-fluorescence quencher was used in the last step. Fluorescence images were acquired using CLSM. Ex/Em: 488/500-550 nm.

6.12 RNA-Seq Analysis

MCF-7 cells were seeded in 60 mm dishes and incubated with **PyPS-NF** (1 μM) for 6 h, followed by light irradiation. After 2 h incubation, the cells were washed with cold PBS and collected for RNA-seq analysis (Illumina Novaseq 6000, San Diego).

6.13 Cell Viability Assay

Live/Dead staining under normoxia and hypoxia. MCF-7 Cells were incubated with different photosensitizers for 2 h (**PyPS-1**) or 6 h (**PyPS-NF**) under normoxia or hypoxia, then subjected to light irradiation (Xe lamp, 490-700 nm, 10 mW/cm^2) for 10 min and incubated for additional 2 h. After washing with PBS, the cells were stained with Calcein AM (1.5 μM) and propidium iodide (PI, 6.0 μM). After staining for 30 min, the cells were washed with PBS and imaged immediately by an inverted fluorescence microscope.

MTT assay under normoxia and hypoxia. MCF-7 cells were seeded in 96-well plates and incubated under normoxia or hypoxia. Then, the cells were incubated with photosensitizers of varying concentrations for 2 h (MB, **PyPS-1, PyPS-2**,

and **PyPS-3**) or 6 h (**PyPS-NF**), washed with PBS and subjected to light irradiation (Xe lamp, 490-700 nm, 10 mW/cm^2) for 10 min. After 12 h incubation, freshly prepared MTT solution (20 μL, 5 mg/mL) in culture medium was added into each well. The cells were further incubated for 4 h at 37 °C. The MTT medium in each well was then carefully removed and replaced by DMSO (150 μL). The plate was gently agitated to dissolve all the precipitates formed. The absorbance at 490 nm was monitored by the microplate reader (SpectraMax i3x, MD). Cell viability was expressed by the ratio of absorbance of the cells incubated with photosensitizers to that of the cells incubated with culture medium only.

Notably, for experiments in hypoxia, cell culture and treatments were conducted in a hypoxia incubator chamber, and all the DMEM used were bubbled with mixed gas (2% O_2, 5% CO_2, and 93% N_2) for 5 min in advance.

7. *In Vivo* Studies

7.1 *In Vivo* Fluorescent Imaging

In vivo fluorescence imaging of tumor-bearing mice was conducted on NightOWL II LB983 In Vivo Imaging System (Berthold Technologies). The excitation wavelength was set at 630 nm and the fluorescence was collected within the wavelength range of 685-715 nm.

Intratumoral Administration: After the tumor size reached appropriately 300 mm^3, these mice were randomized into the following three groups (n = 3): (i) injection of **PyPS-NF** (0.5 mg/kg) into tumors; (ii) injection of **PyPS-NF** (0.5 mg/kg) into dicumarin (DIC)-pretreated tumors, and (iii) injection of **PyPS-NF** (0.5 mg/kg) into healthy hindlimb muscles. For group ii, 50 μL DIC in PBS (0.2 mM) was injected into the tumors 1 h prior to the **PyPS-NF** injection. Fluorescence images were captured at 0, 0.5, 1, 2, 4, and 8 h post-injection, respectively.

Intravenous Administration: After the tumor size reached appropriately 300 mm^3, **PyPS-NF** (5 mg/kg) was injected intravenously and fluorescence images were captured at 0, 2, 4, 8, 12, 24, and 48 h post-injection, respectively. After 48 h post-injection, the mice were sacrificed, and tumor tissue and main organs (heart, liver, spleen, lung, and kidneys) were collected for *ex vivo* fluorescence imaging.

7.2 *In Vivo* Biodistribution Analysis

4T1 tumor-bearing BALB/c mice (n = 5) were intravenously injected with 200 μL **PyPS-CG** (**PyPS-NF** or **PyPS-Et**). The mice were sacrificed at 24 h post-injection and dissected to collect the main organs (including the heart, liver, spleen, lung, and kidney) and tumor tissues. These tissues were weighed and homogenized in 1 mL of ice-cold saline. The obtained tissue homogenates were extracted with CH_2Cl_2. After removing the organic solvent, the residues were dissolved in methanol (500 μL), and analyzed by HPLC to determine the levels of PyPS-CG in both caged and activated forms in different tissues, which were presented as % ID g^{-1}.

7.3 *In Vivo* Antitumor Estimation

PyPS-NF-mediated photoimmunotherapy was investigated in mice inoculated with 4T1 cancer cells in the right flank as the primary tumors. These tumor-bearing mice were randomized into four groups (n = 5): (i) only PBS (200 μL), (ii) PBS + light (633 nm, 0.1 W/cm^2, 10 min), (iii) only **PyPS-NF**, and (iv) **PyPS-NF** + light. **PyPS-NF** (5 mg/kg, 200 μL)

was intravenously injected twice on day -1 and day 1, and then irradiated with a 633 nm laser (0.1 W/cm^2, 10 min) after 24 h of each injection. The tumor size and body weight were recorded every other day. Tumor volume was calculated according to the formula: volume = length × width^2 / 2. After 14 days of observation, the mice were sacrificed. Major organs (heart, liver, spleen, lung, kidney) and tumors were collected for hematoxylin and eosin (H&E), immunohistochemistry (IHC) staining, and immune cell staining analyses.

To further assess the immunotherapeutic outcome mediated by **PyPS-NF**, a second *in vivo* experiment was carried out. After primary tumors were treated with **PyPS-NF**-mediated PDT at 3 days, 4T1 cells (~1 × 10^6) suspended in 100 µL of PBS were subcutaneously injected into the left flank of each mouse. At day 14, the mice were sacrificed and tumors were harvested and weighted.

The data herein were expressed as means ± standard deviation (SD). Statistical significance was determined using two-way analysis of variance and $P < 0.05$ was considered to be statistical significance (*** indicate $P < 0.001$).

7.4 Immune Analysis

To systematically study antitumor immune response activation *in vivo*, a panel of immune cells were analyzed by flow cytometry after staining with antibodies. Lymph gland and tumor tissues were dissected from mice (n = 3 mice per group) and cut into small pieces. The single-cell suspensions were prepared via mechanical grinding in ice-cold PBS. After filtration through a 70 µm nylon cell strainer, red blood cells were removed using red blood cell lysis buffer. For DCs analysis, the single-cell suspensions were incubated with PE anti-mouse CD11c, APC anti-mouse CD80, and FITC anti-mouse CD86. For T cells analysis, the single-cell suspensions were incubated with PE anti-mouse CD3, APC anti-mouse CD8a, and FITC anti-mouse CD4. After that, the cells were evaluated by flow cytometry.

7.5 Serum Cytokines Levels

After primary tumors were treated with **PyPS-NF**-mediated PDT at 14 days, blood was harvested from the eye socket and serum was isolated. The levels of IL-1β, IFN-γ, and TNF-α in serum were determined using IL-1β ELISA kits (Boster EK0394), IFN-γ ELISA kits (Boster EK0375), and TNF-α ELISA kits (Boster EK0527), respectively.

8 Supplementary Figures

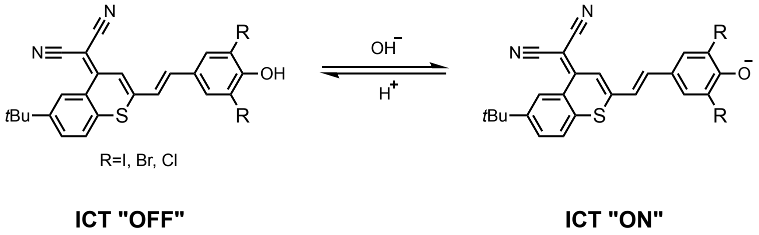

Scheme S1. Schematic illustration of the pH-responsiveness of the synthesized **PyPS** molecules.

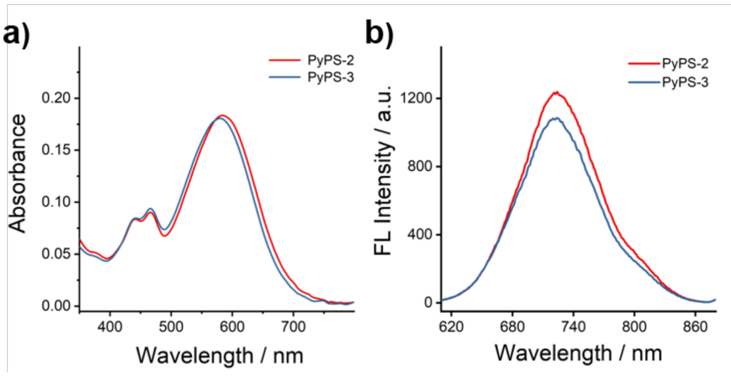

Figure S2. (a) UV-vis absorption and (b) fluorescence emission spectra recorded for 5.0 μM aqueous solutions of **PyPS-2** and **PyPS-3** at pH 7.4. Ex/Em slit: 5/5 nm.

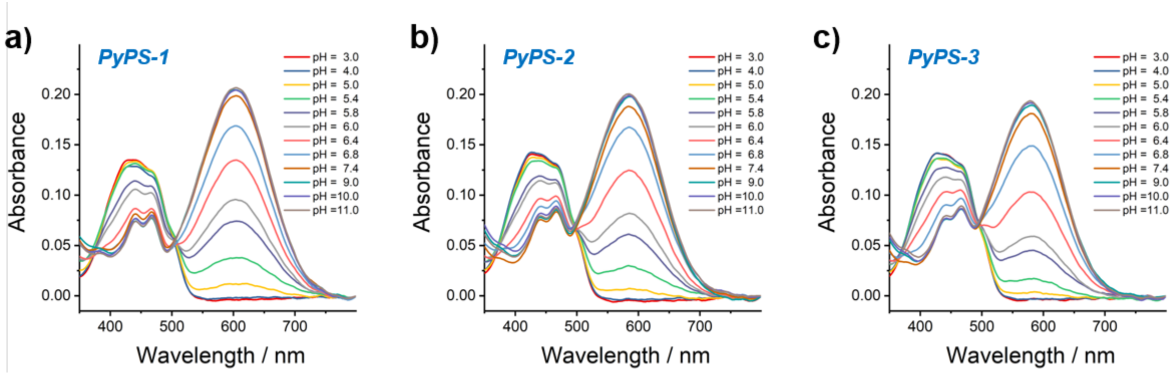

Figure S3. pH-Dependent UV−vis absorption spectra recorded for 5.0 μM aqueous solutions of (a) **PyPS-1**, (b) **PyPS-2**, and (c) **PyPS-3**.

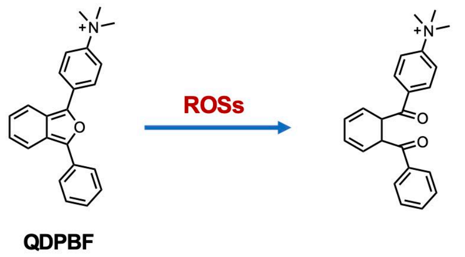

Scheme S2. Schematic illustration of QDPBF for the total ROS detection.

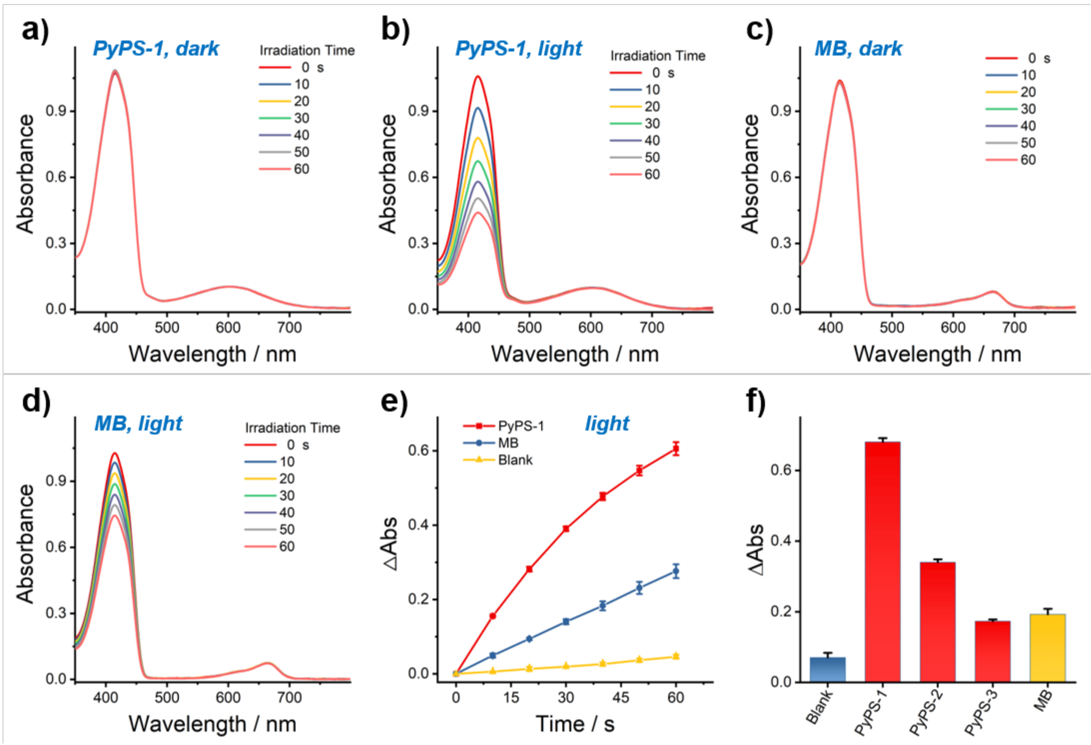

Figure S4. Time-dependent UV-vis absorption spectra of QDPBF within 2.5 μM aqueous solution of (a,b) **PyPS-1** or (c,d) MB under different light conditions. (e) Time-dependent QDPBF consumption in the absence or presence of different samples as indicated upon light irradiation. (f) QDPBF consumption of MB and all three **PyPS** molecules after light irradiation for 1 min. Light source: Xe lamp (490-700 nm, 5 mW/cm^2). Error bars represent means ± SD (n = 3).

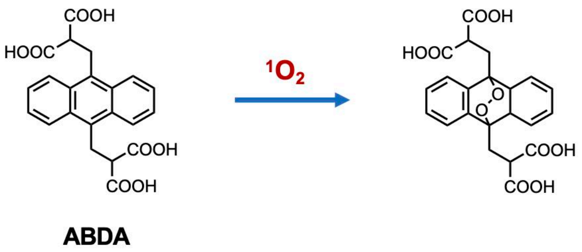

Scheme S3. Schematic illustration of ABDA for $^1O_2$ detection.

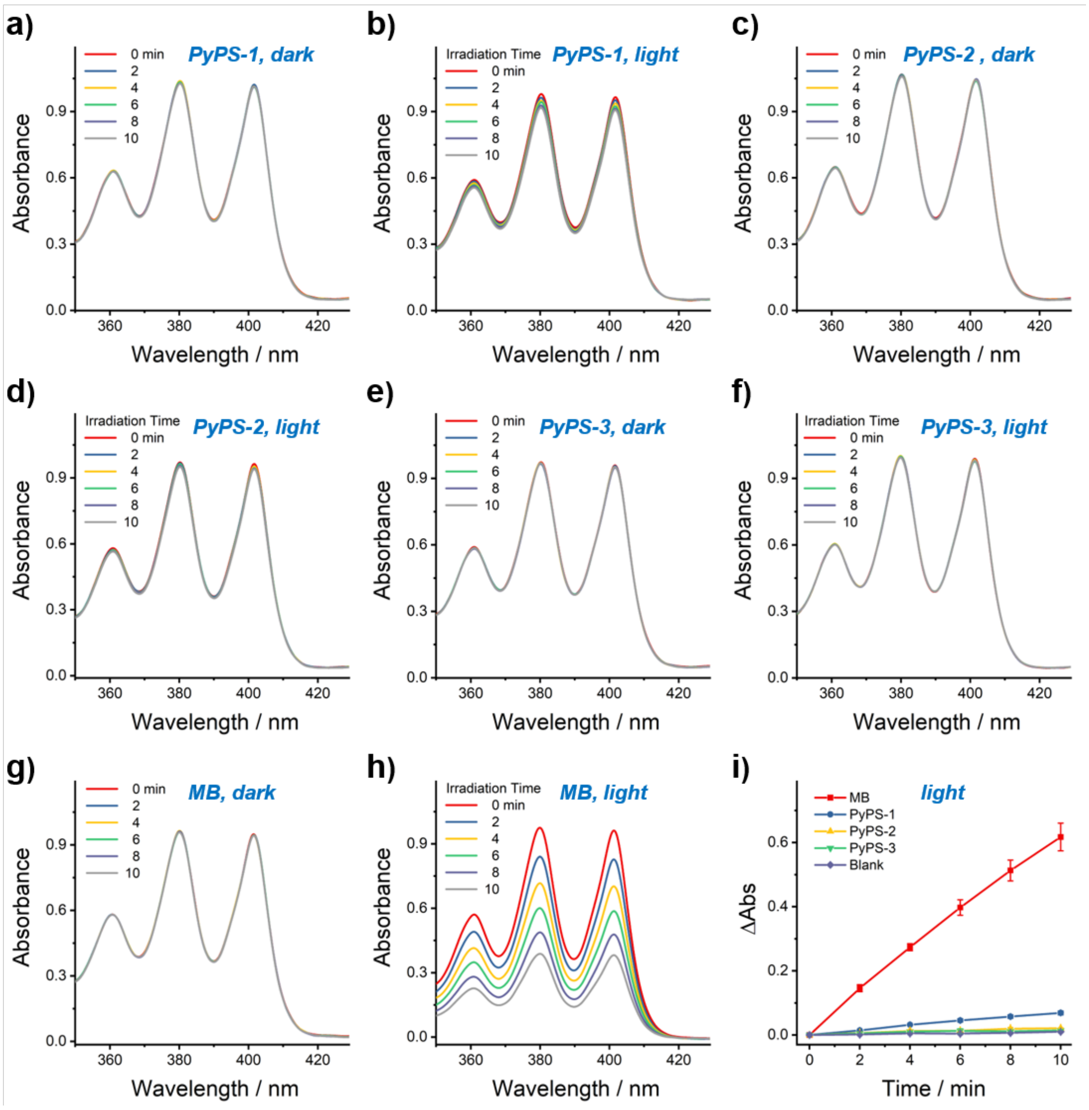

Figure S5. Time-dependent UV-vis absorption spectra of QDPBF within 2.5 μM aqueous solution of (a,b) **PyPS-1**, (c,d) **PyPS-2**, (e,f) **PyPS-3**, or (g,h) MB under different light conditions. (i) Time-dependent QDPBF consumption in the absence or presence of different samples as indicated upon light irradiation. Light source: Xe lamp (490-700 nm, 5 mW/cm^2). Error bars represent means ± SD (n = 3).

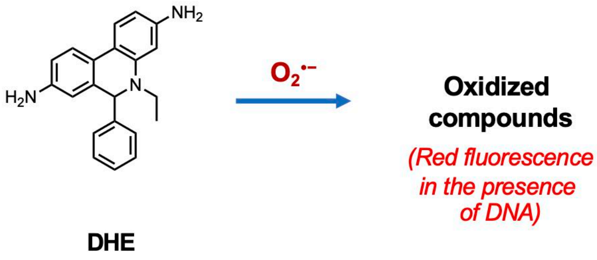

Scheme S4. Schematic illustration of DHE for $O_2^{\bullet-}$ detection.

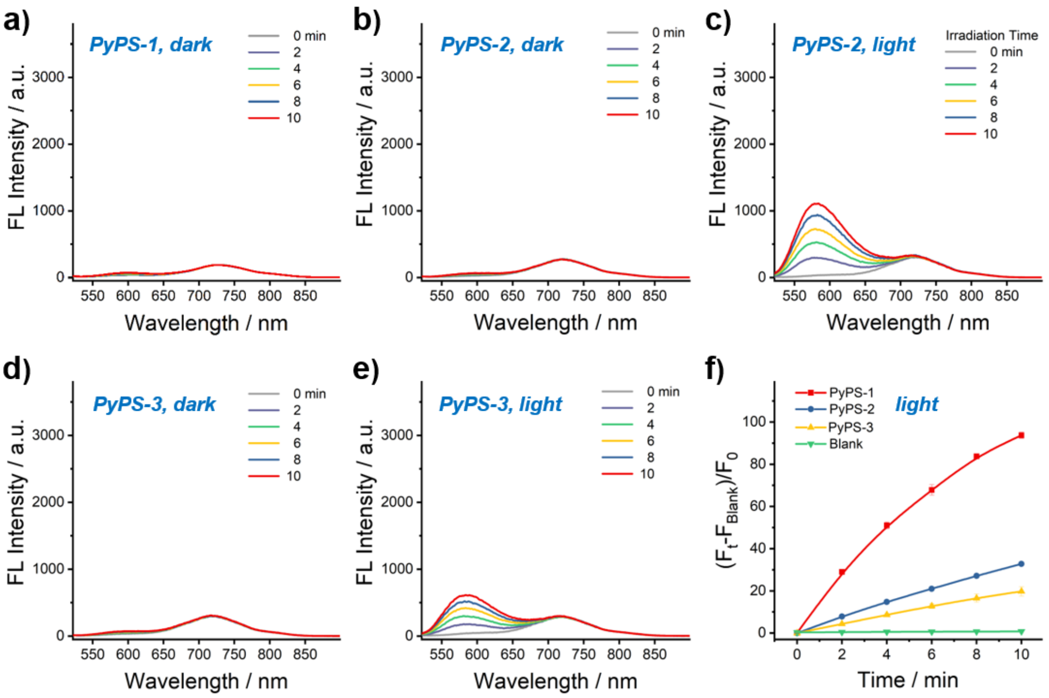

Figure S6. Time-dependent fluorescence emission spectra of DHE (containing 40 μg/mL DNA-*ct*) within 2.5 μM aqueous solution of (a) **PyPS-1**, (b,c) **PyPS-2**, or (d,e) **PyPS-3** under different light conditions. (f) Time-dependent fluorescence emission enhancement of DHE in the absence or presence of different samples as indicated upon light irradiation. Light source: Xe lamp (490-700 nm, 5 mW/cm^2). Error bars represent means ± SD (n = 3).

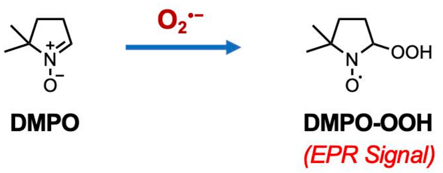

Scheme S5. Schematic illustration of DMPO for $O_2^{\bullet -}$ and $^{\bullet}OH$ detection.

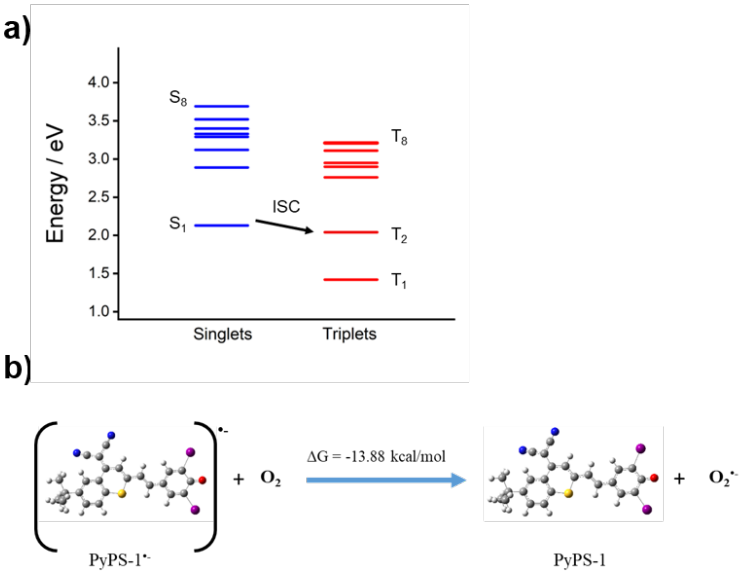

Figure S7. (a) TD-DFT simulation of energy level distributions of excited singlet and triplet states for PyPS-1 based on B3LYP/SMD/def2TZVP level. (b) Gibbs energy profiles for the $O_2^{\bullet -}$ generation through PyPS-1 upon light irradiation.

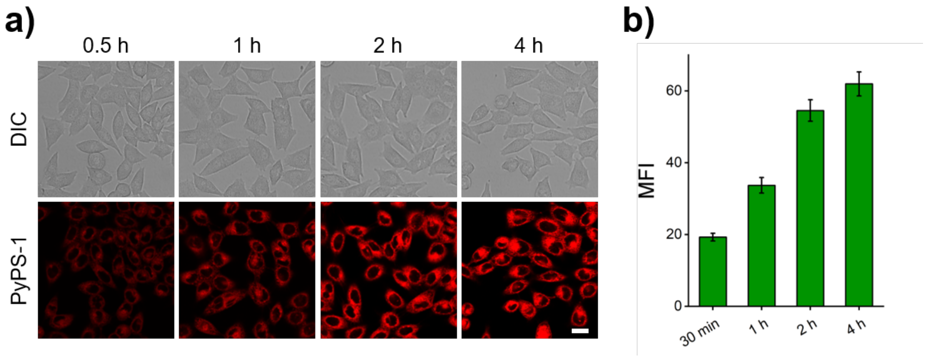

Figure S8. Confocal fluorescence imaging of the cellular uptake of **PyPS-1**. Scale bar: 20 μm.

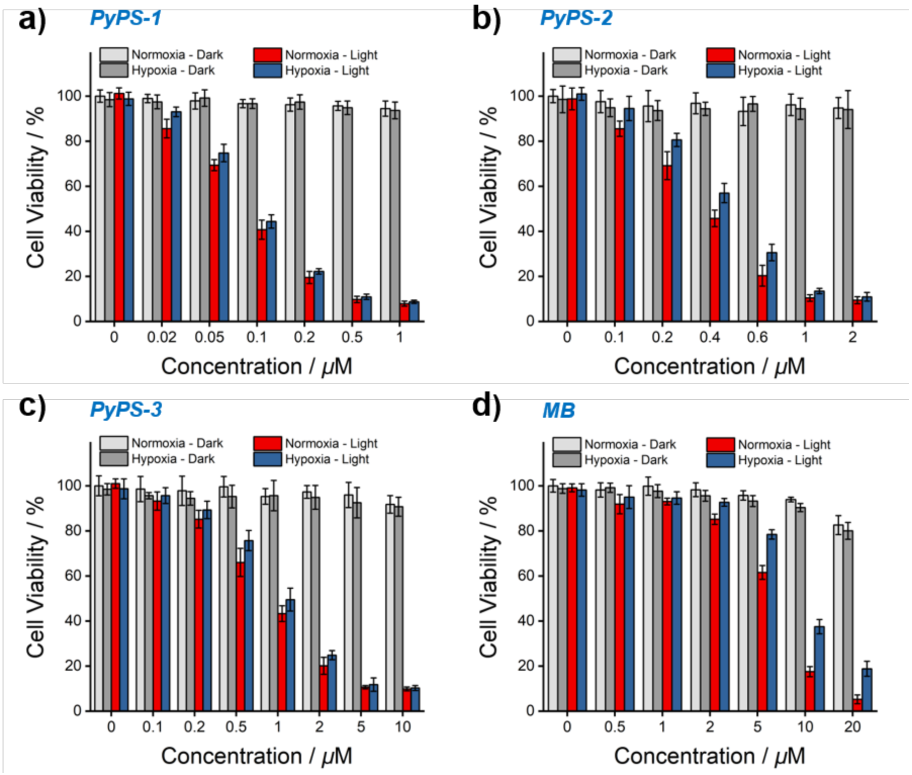

Figure S9. Cell viability of MCF-7 cells incubated with (a) **PyPS-1**, (b) **PyPS-2**, (c) **PyPS-3**, and (d) **MB** in dark or upon light irradiation for 10 min under normoxia and hypoxia determined by MTT assay (n = 4). Light source: Xe lamp (490-700 nm, 10 mW/cm^2).

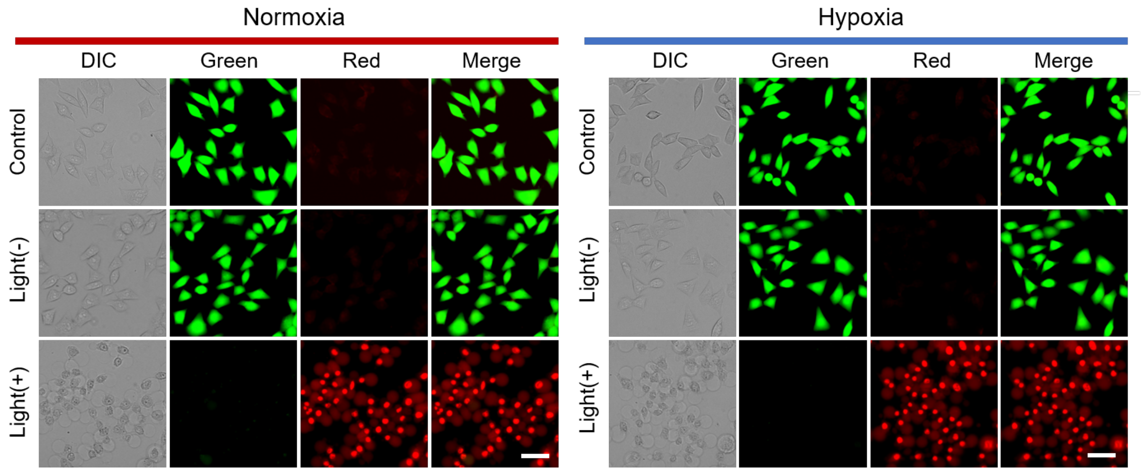

Figure S10. Live/dead staining images of MCF-7 cells. The cells were incubated with **PyPS-1** under normoxic or hypoxic conditions, and then either subjected to light irradiation or incubated in dark for 10 min. After incubation for 2 h, the cells were stained with calcein AM and PI. Concentration: 1.0 µM **PyPS-1**. Scale bar: 50 µm. Light source: Xe lamp (490-700 nm, 10 mW/cm^2).

Table S1. IC_{50} values of different PyPSs under normoxic and hypoxic conditions.

Sample	IC_{50} / µM (normoxia)	IC_{50} / µM (hypoxia)
PyPS-1	0.083	0.106
PyPS-2	0.325	0.426
PyPS-3	0.811	1.098
MB	4.811	8.473

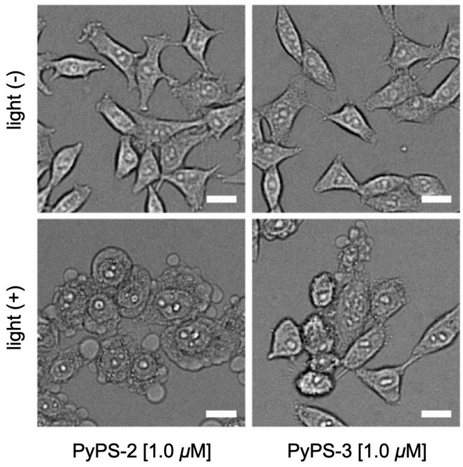

Figure S11. Representative phase contrast images of MCF-7 cells incubated with **PyPS-2** (1 µM) or **PyPS-3** (1 µM) in dark or upon light irradiation. Scale bar: 20 µm.

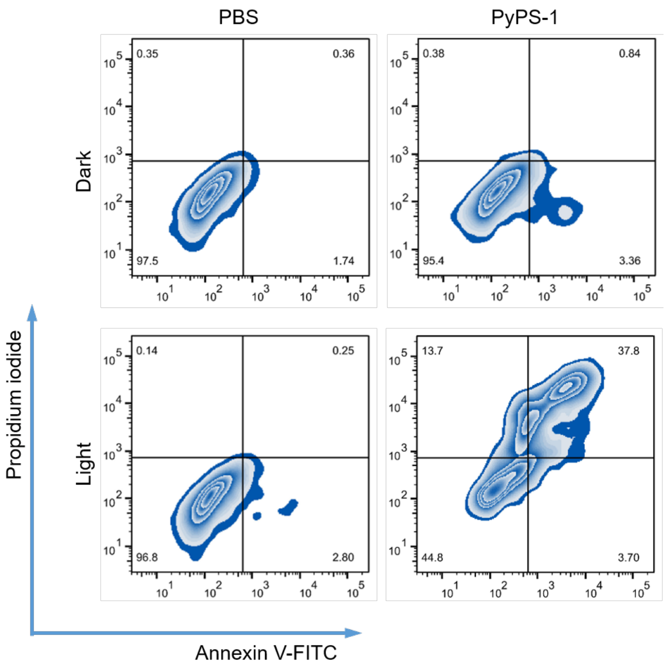

Figure S12. Flow cytometry of propidium iodide (PI) and annexin V-FITC-stained MCF-7 cells after different treatments.

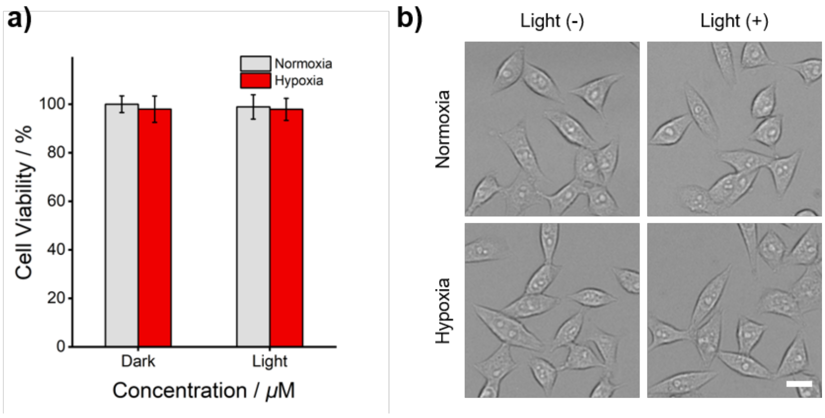

Figure S13. (a) Cell viability of MCF-7 cells (n = 4) and (b) representative phase contrast images of MCF-7 cells in dark or upon light irradiation for 10 min under normoxia and hypoxia. Light source: Xe lamp (490-700 nm, 10 mW/cm^2, 10 min). Scale bar: 20 μm.

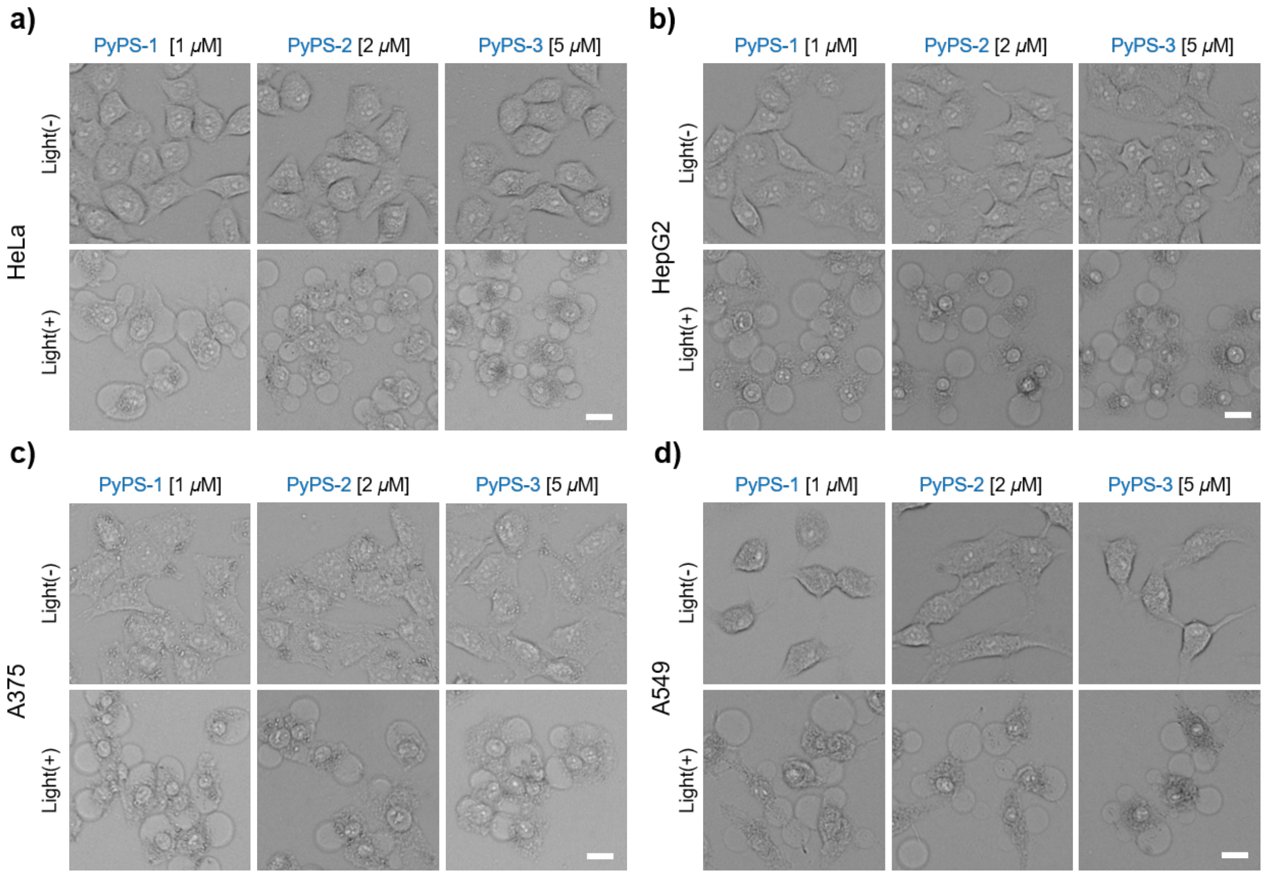

Figure S14. Representative phase contrast images of different cell lines incubated with **PyPS-1**, **PyPS-2**, or **PyPS-3** in dark or upon light irradiation. (a) HeLa cells, (b) HepG2 cells, (c) A375 cells, and (d) A549 cells. Scale bar: 20 μm. Light source: Xe lamp (490-700 nm, 10 mW/cm^2, 10 min).

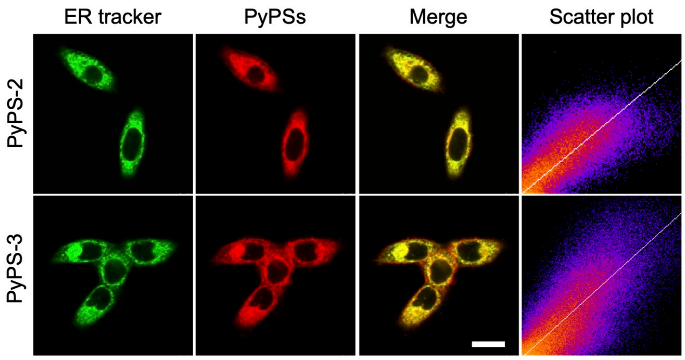

Figure S15. Colocalization studies of **PyPS-2** and **PyPS-3** toward ER in MCF-7 cells. Scale bar: 20 μm. Pearson's correlation coefficients for **PyPS-2** and **PyPS-3** are 0.92 and 0.88, respectively.

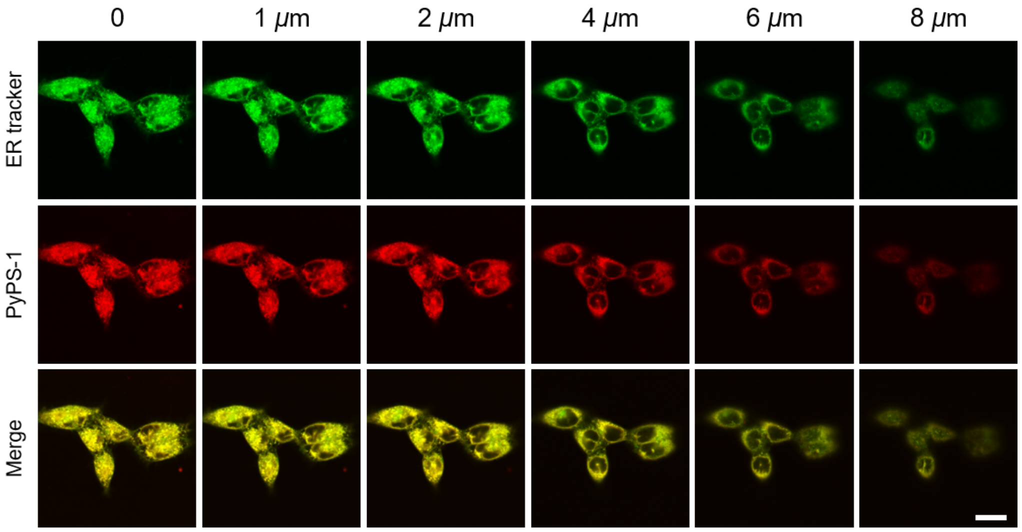

Figure S16. Colocalization imaging of PyPS-1 with a commercial specific ER probe in different z-axis sections. Scale bar: 20 μm.

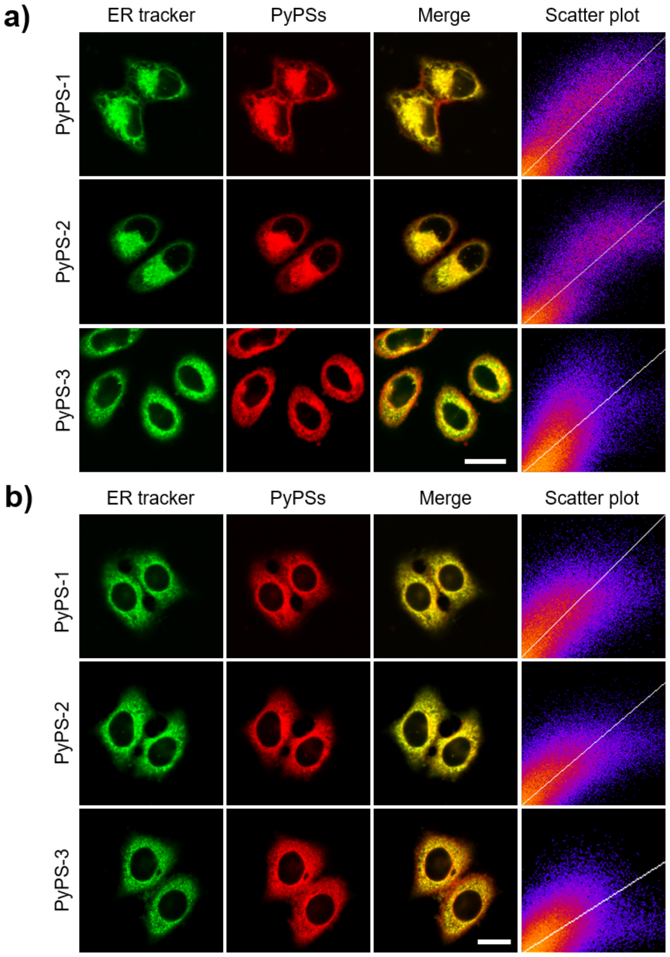

Figure S17. (a) Colocalization studies of three **PyPS** molecules toward ER in HeLa cells. Pearson's correlation coefficients for **PyPS-1**, **PyPS-2**, and **PyPS-3** are 0.95, 0.92, and 0.85, respectively. (b) Colocalization studies of three **PyPS** molecules toward ER in HepG2 cells. Pearson's correlation coefficients for **PyPS-1**, **PyPS-2**, and **PyPS-3** are 0.92, 0.90, and 0.86, respectively. Scale bar: 20 μm.

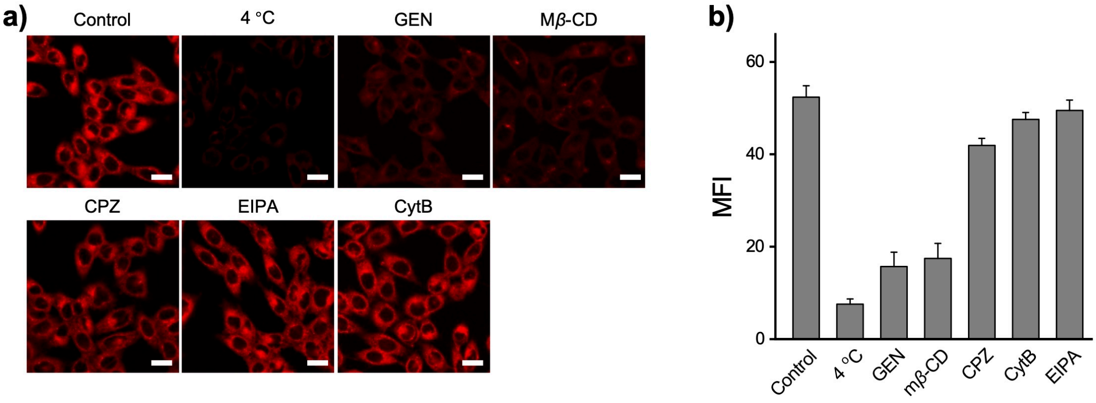

Figure S18. (a) CLSM images and (b) MFI of MCF-7 cells incubated with **PyPS-1** (5 μM) at 4 °C or in the presence of different cell uptake inhibitors at 37 °C. GEN (an inhibitor of caveolae-mediated endocytosis), M*β*-CD (an inhibitor of lipid raft-mediated endocytosis), CPZ (an inhibitor of clathrin-mediated endocytosis), EIPA (an inhibitor of macropinocytosis), and CytB (an inhibitor of phagocytosis and/or macropinocytosis). Scale bar: 20 μm.

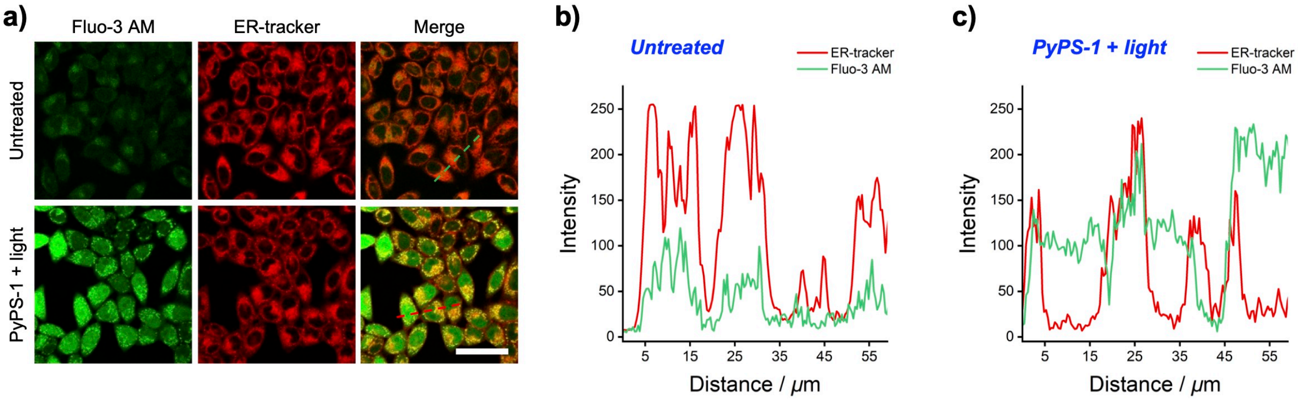

Figure S19. CLSM images of MCF-7 cells co-stained with Fluo-3 AM and ER-tracker. Scale bar: 50 μm. Intensity profiles along the (b) green line of untreated group and (c) red line of **PyPS-1**/light group.

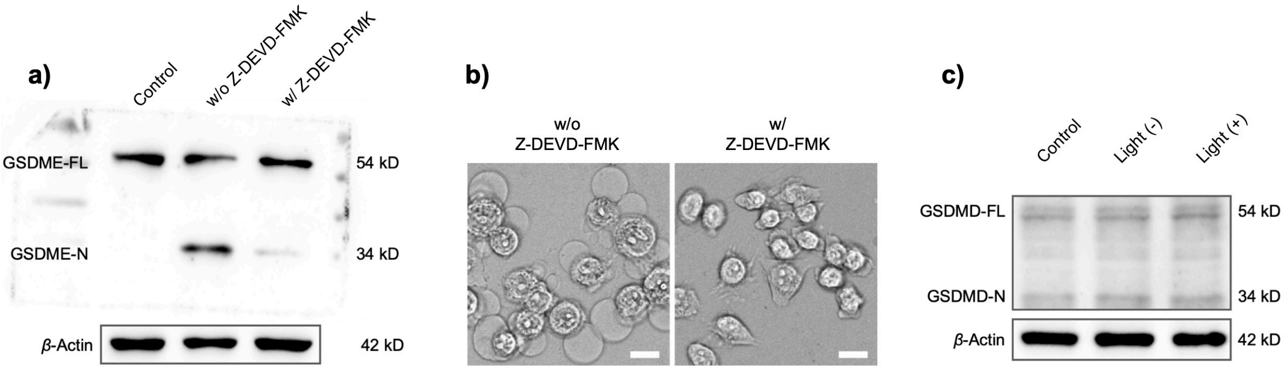

Figure S20. (a) Representative phase contrast images of MCF-7 cells and (b) western blotting of the cleavage of GSDME in the cells after treatment of **PyPS-1**/light. Scale bar: 20 μm. Note that the cells were pretreated with or without Z-DEVD-FMK (a caspase-3 inhibitor). (c) Western blotting of GSDMD in MCF-7 cells after treatment of **PyPS-1**/light.

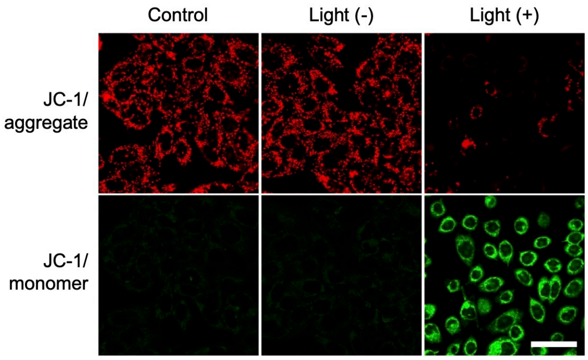

Figure S21. Evaluation of the mitochondrial damage of MCF-7 cells treated with **PyPS-1** (1.0 μM) under light irradiation under hypoxic conditions by using JC-1 as the fluorescent indicator. JC-1/Mono: Ex/Em 488/500-530 nm; JC-1/Agg: Ex/Em 543/560-600 nm. Scale bar: 50 μm. Light source: Xe lamp (490-700 nm, 10 mW/cm^2, 5 min).

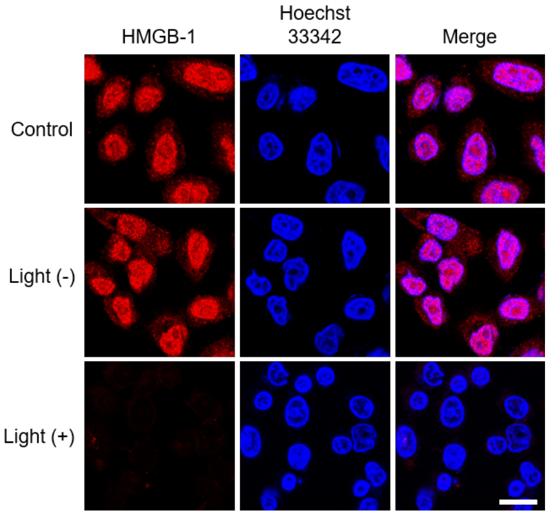

Figure S22. Investigation of HMGB-1 release in MCF-7 cells after **PyPS-1**-mediated PDT under hypoxic conditions. Scale bar: 20 μm. Light source: Xe lamp (490-700 nm, 10 mW/cm^2, 10 min).

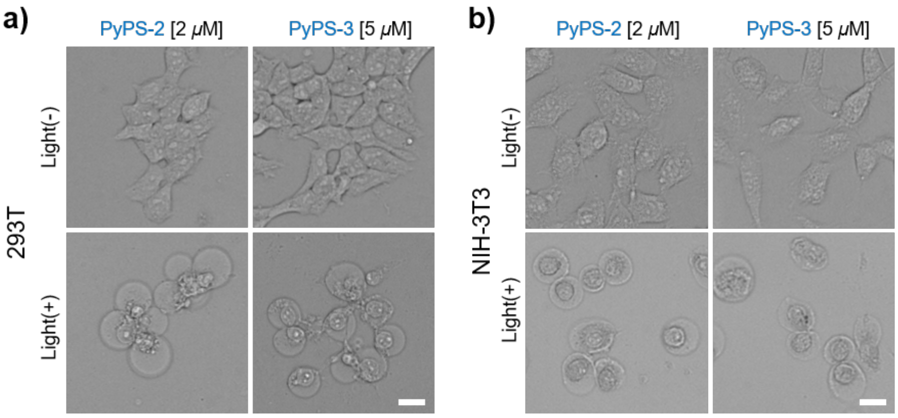

Figure S23. Representative phase contrast images of different healthy cell lines incubated with **PyPS-2** or **PyPS-3** in dark or upon light irradiation. (a) 293T cells, (b) NIH-3T3 cells. Scale bar: 20 μm. Light source: Xe lamp (490-700 nm, 10 mW/cm^2, 10 min).

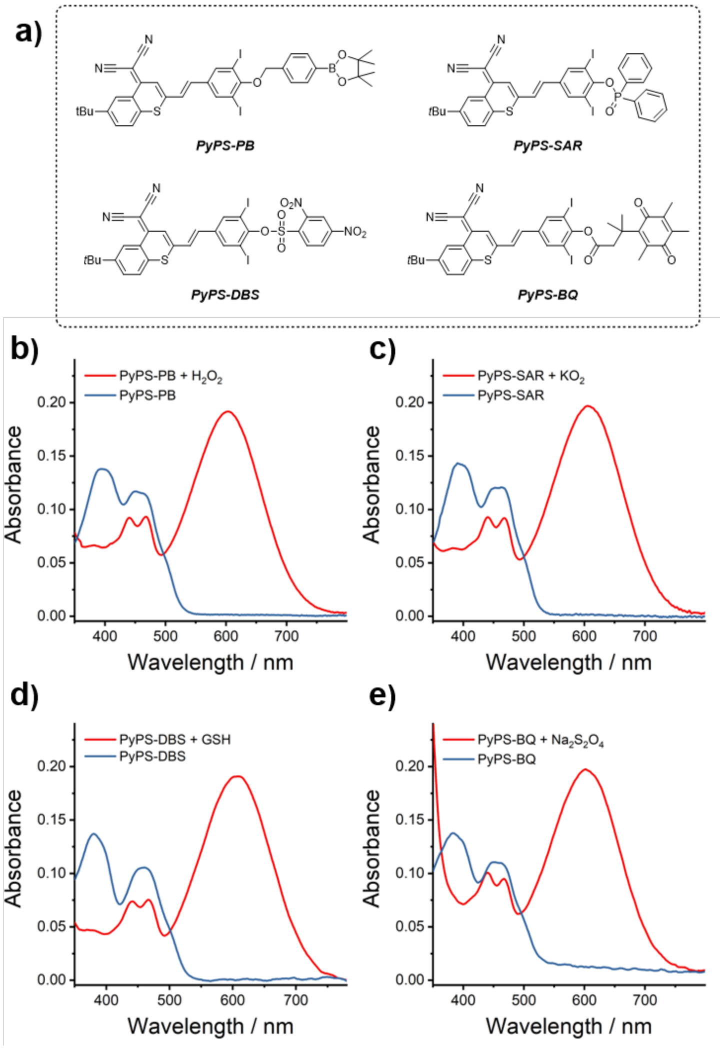

Figure S24. (a) Chemical structures of hydrogen peroxide-activatable **PyPS-PB**, superoxide-activatable **PyPS-SAR**, GSH-activatable **PyPS-DBS**, and NQO1-activatable **PyPS-BQ**. (b-e) UV-vis absorption spectra of 5.0 µM activatable **PyPS** molecules before and after treatment with relevant stimulus in aqueous media. (b) **PyPS-PB** + hydrogen peroxide, (c) **PyPS-SAR** + KO_2 (as $O_2^{\bullet-}$ source), (d) **PyPS-DBS** + GSH, (e) **PyPS-BQ** + $Na_2S_2O_4$ (as a NQO1 mimic).

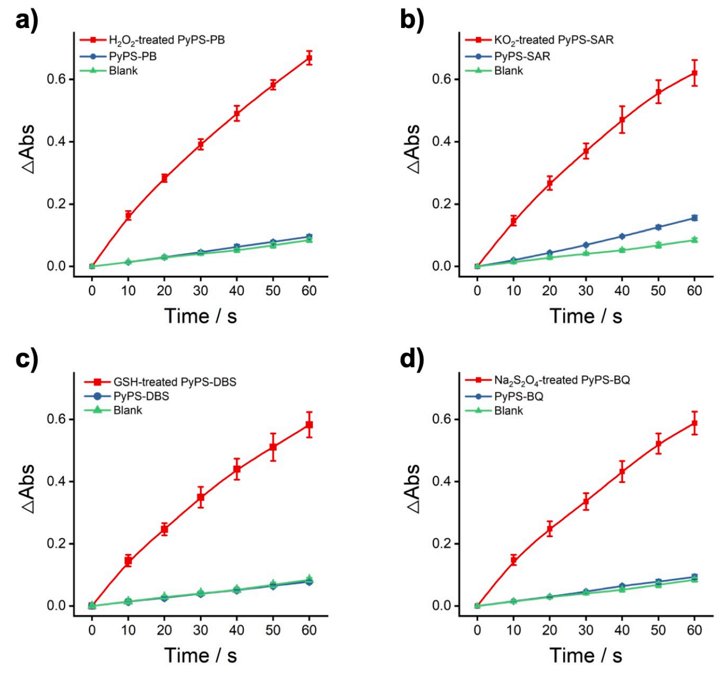

Figure S25. Time-dependent QDPBF consumption in the presence of caged or activated **PyPS-CG** molecules as indicated upon light irradiation. Error bars represent means ± SD (n = 3). Light: Xe lamp (490-700 nm, 5 mW/cm^2).

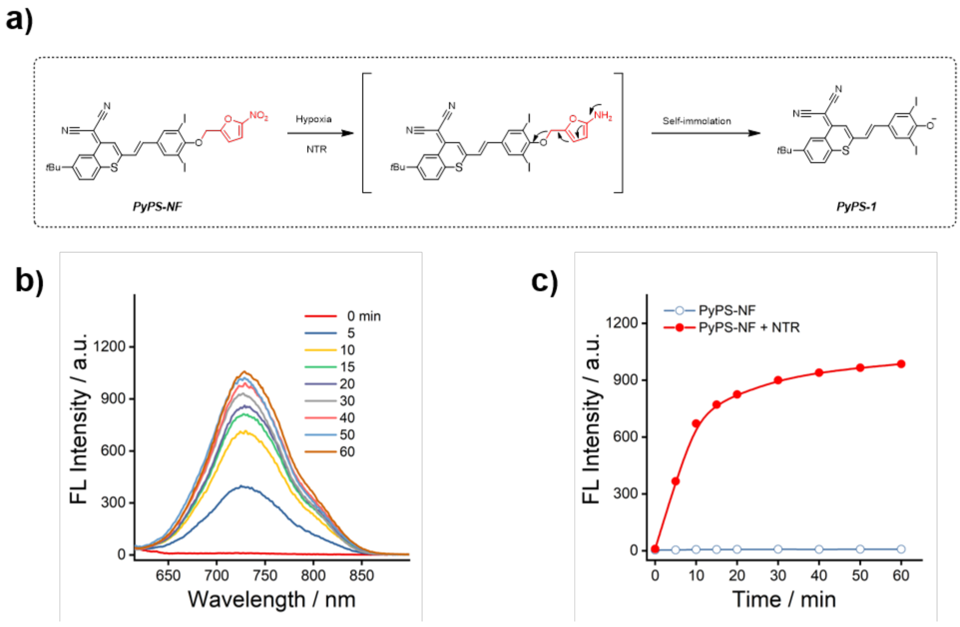

Figure S26. (a) NTR induced conversion of PyPS-NF into PyPS-1 under hypoxic condition. Time-dependent fluorescence emission spectra (b) and emission intensities (c) recorded for the aqueous **PyPS-NF** (5.0 µM) solution containing 0.5 mM NADH upon incubating with NTR (5 µg/mL) at 37 °C

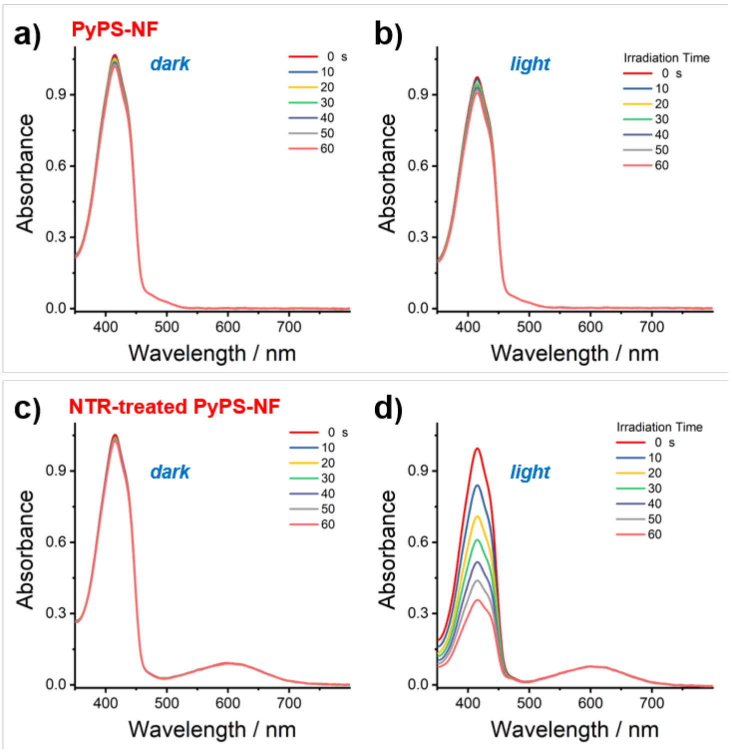

Figure S27. Time-dependent UV-vis absorption spectra of QDPBF in the presence of (a,b) **PyPS-NF** or (c,d) NTR-treated **PyPS-NF** in dark or upon light irradiation. Light sources: Xe lamp (490-700 nm, 5 mW/cm^2). Error bars represent mean ± SD (n = 3).

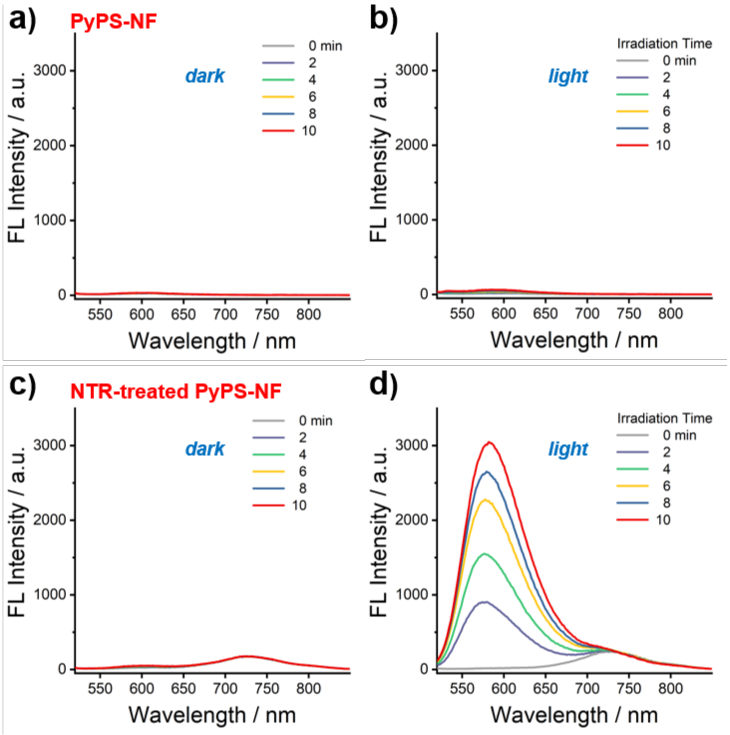

Figure S28. Time-dependent fluorescence emission spectra of DHE in the presence of ((a,b) **PyPS-NF** or (c,d) NTR-treated **PyPS-NF** in dark or upon light irradiation. Light sources: Xe lamp (490-700 nm, 5 mW/cm^2). Error bars represent mean ± SD (n = 3).

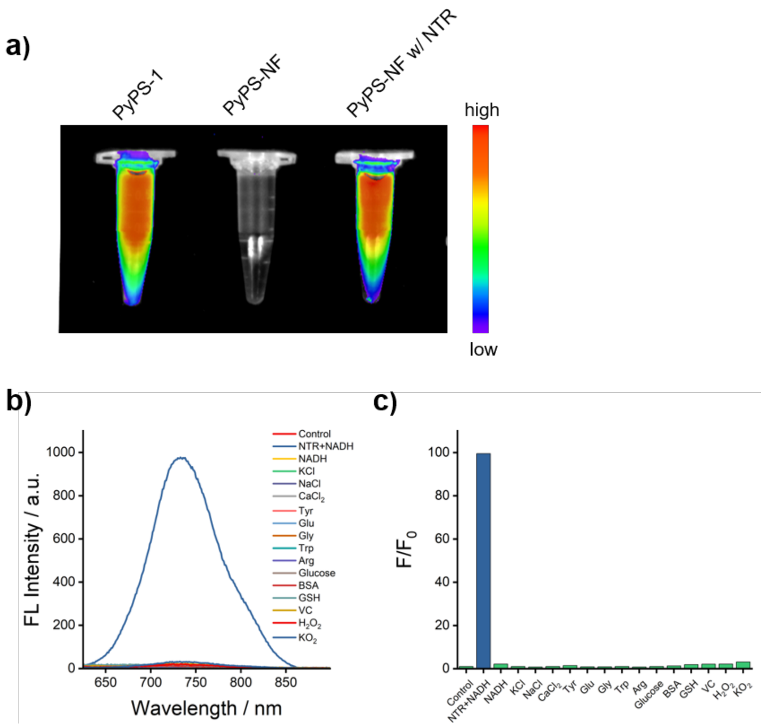

Figure S29. (a) Fluorescence imaging of aqueous solutions of **PyPS-1**, **PyPS-NF**, and NTR-treated **PyPS-NF** by using *in vivo* imaging system. (b) Fluorescence emission spectra and (c) the intensity enhancement at 730 nm (F/F_0) of **PyPS-NF** in the presence of different kinds of species, control (**PyPS-NF** only), and with NTR (5 µg/mL) + NADH (0.5 mM), NADH (1 mM), KCl (50 mM), NaCl (50 mM), $CaCl_2$ (50 mM), tyrosine (Tyr, 1 mM), glutamic acid (Glu, 1 mM), glycine (Gly, 1 mM), tryptophan, arginine (Arg, 1 mM), glucose (10 mM), BSA (10 mg/mL), GSH (5 mM), VC (1 mM), H_2O_2 (1 mM), and KO_2 (1 mM).

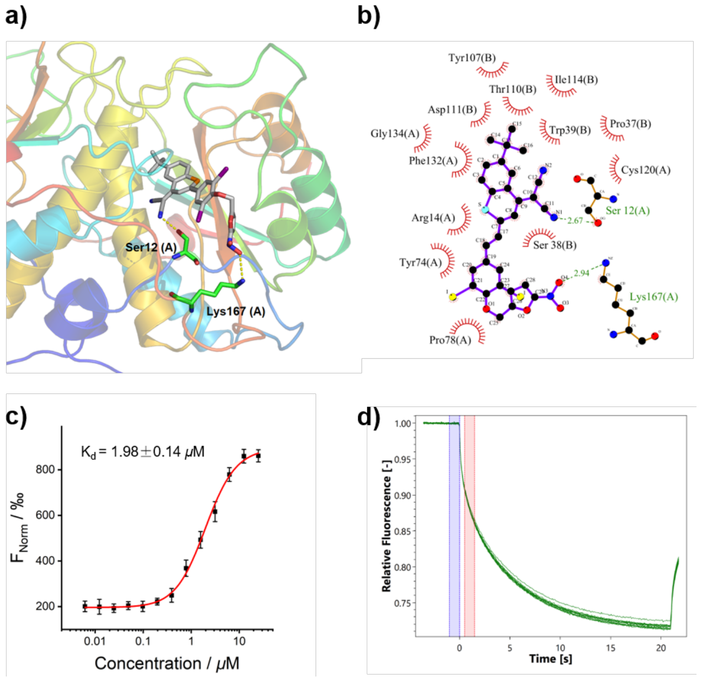

Figure S30. Details of the binding of **PyPS-NF** to NTR, showing the (a) hydrogen bonding (dotted lines) and (b) hydrophobic interactions (red curves in b) between **PyPS-NF** and the residues in the binding pocket of NTR. (c) Binding affinity between **PyPS-NF** and NTR measured by MST. (d) MST traces of binding tests.

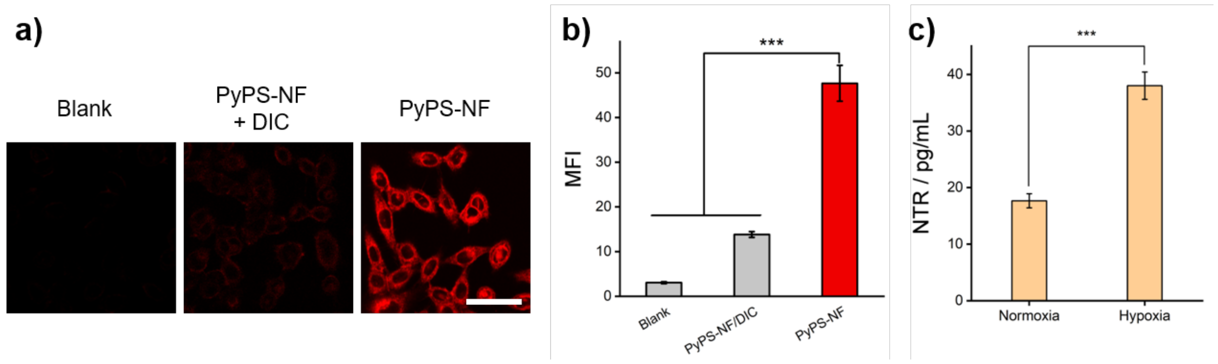

Figure S31. (a) Confocal fluorescence imaging of MCF-7 cells after different treatments under hypoxic conditions: untreated (left), incubation with **PyPS-NF** and DIC (middle), or incubation with **PyPS-NF** (right). (b) MFI quantified from the CLSM images (n = 3). Scale bar: 50 μm. (c) NTR levels in both normoxic and hypoxic MCF-7 cells determined by ELISA assay (n = 4). ***p < 0.001 (Student's t test).

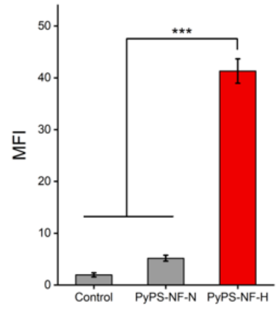

Figure S32. Confocal fluorescence imaging of PyPS-NF in response to hypoxia and mean fluorescence intensities (MFI) quantified from the CLSM images (n = 3).

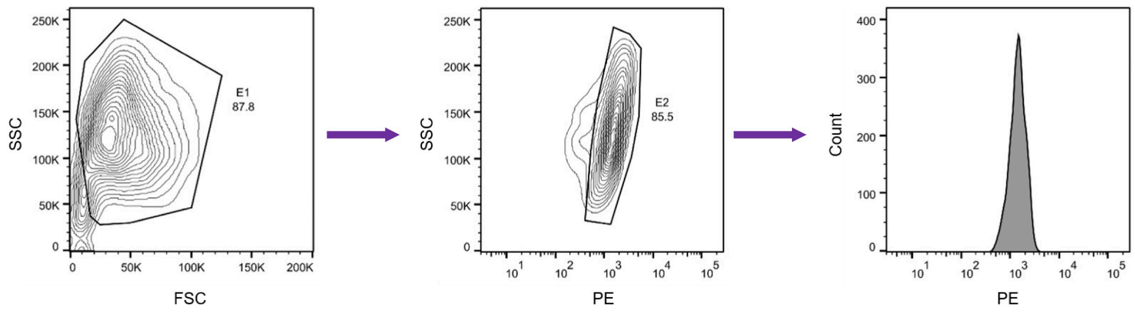

Figure S33. Gating strategy for flow cytometry analysis of the ROS levels in MCF-7 cells.

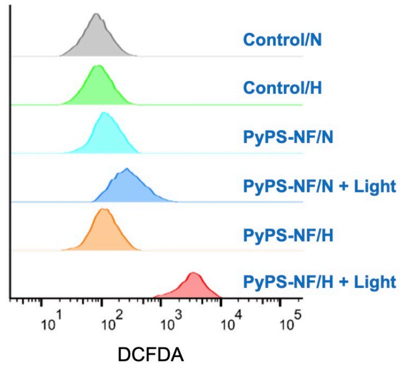

Figure S34. Flow cytometry analysis of the ROS levels in MCF-7 cells after different treatments using DCF-DA as ROS fluorescence probe. N and H represent normoxia and hypoxia, respectively.

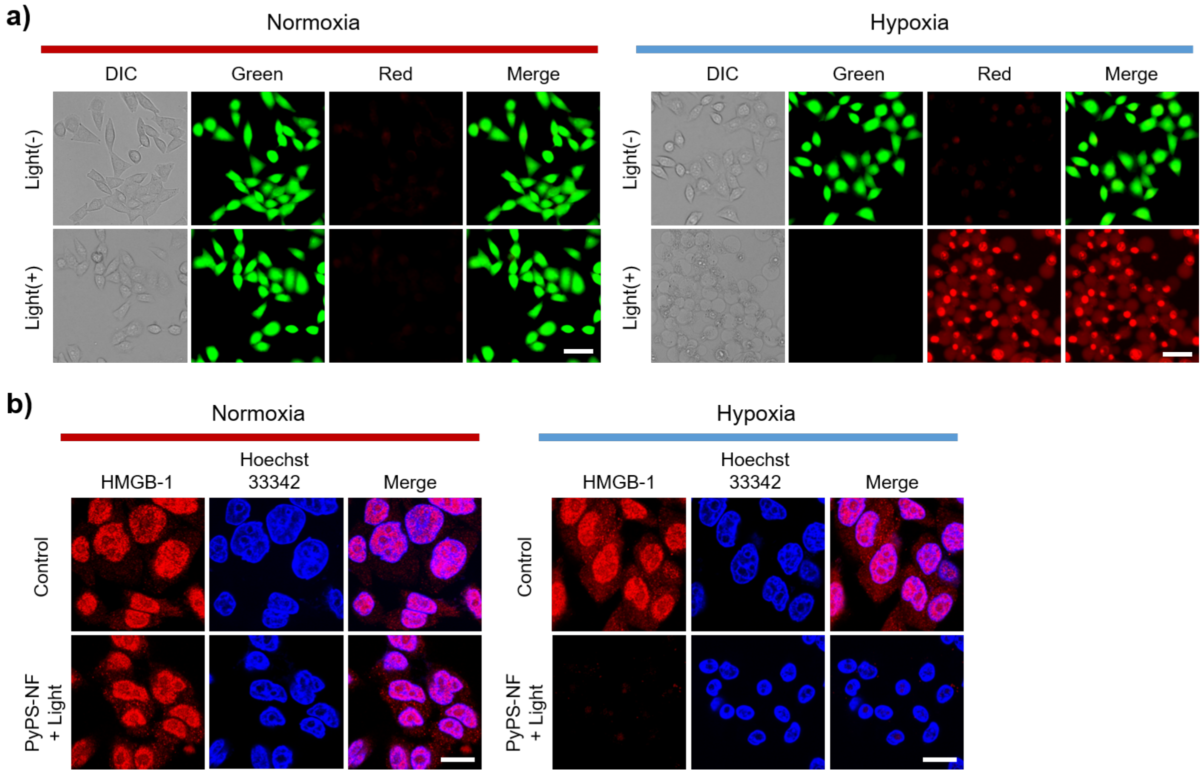

Figure S35. (a) Live/dead staining images of MCF-7 cells. The cells were incubated with **PyPS-NF** under normoxic or hypoxic conditions, and then either subjected to light irradiation or incubated in dark for 10 min. After incubation for 2 h, the cells were stained with calcein AM and PI. Concentration: 1.0 μM **PyPS-NF**. Scale bar: 50 μm. (b) Investigation of HMGB-1 release in MCF-7 cells. The cells were incubated with **PyPS-NF** under normoxic or hypoxic conditions, and then either subjected to light irradiation or incubated in dark for 10 min. Scale bar: 20 μm. Light source: Xe lamp (490-700 nm, 10 mW/cm^2).

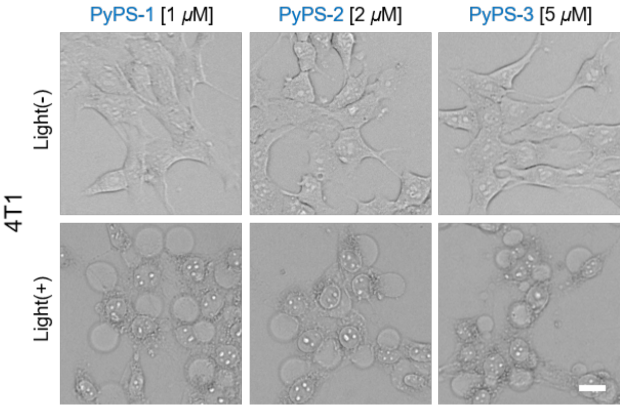

Figure S36. Representative phase contrast images of 4T1 cells incubated with **PyPS-1**, **PyPS-2**, or **PyPS-3** in dark or upon light irradiation. Scale bar: 20 μm. Light source: Xe lamp (490-700 nm, 10 mW/cm^2, 10 min).

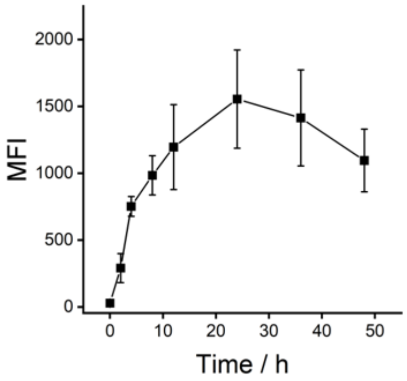

Figure S37. Time-dependent fluorescence intensity in the tumor regions after intravenous injection of **PyPS-NF**.

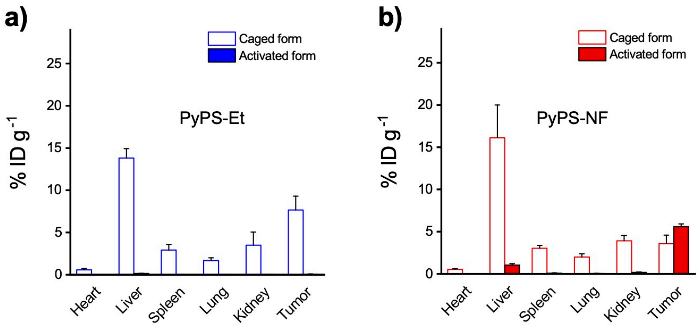

Figure S38 *In vivo* biodistribution of (a) caged or activated form of **PyPS-Et** and (b) caged or activated form of **PyPS-NF** in different main organs and tumor tissues.

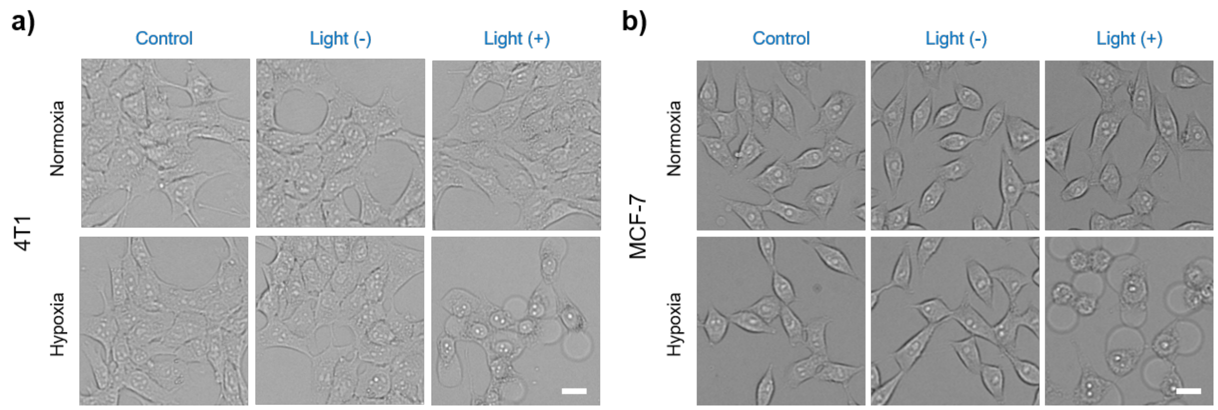

Figure S39. Representative phase contrast images of different cell lines incubated with **PyPS-NF** after different treatments. (a) 4T1 cells, (b) MCF-7 cells. Scale bar: 20 µm. Light source: 633 nm laser (10 mW/cm^2, 10 min).

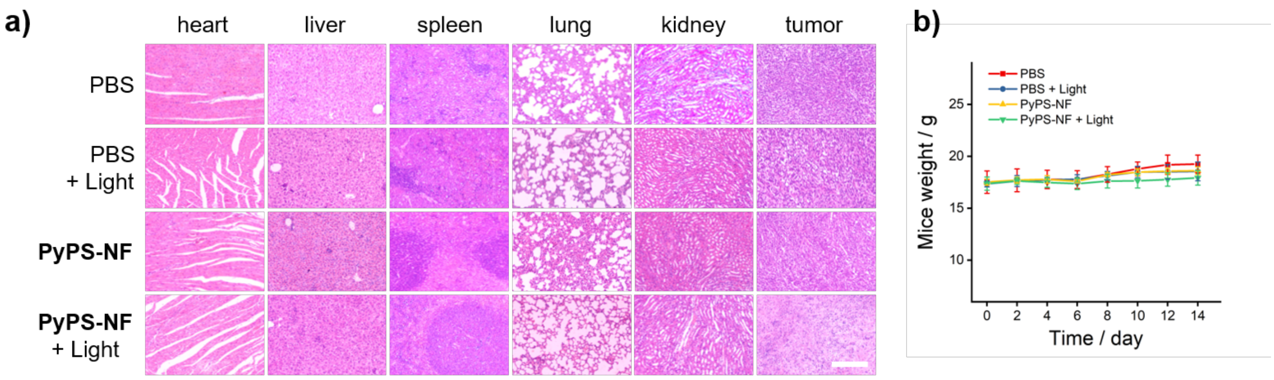

Figure S40. H&E staining images of main organs (heart, liver, spleen, lung, and kidney) and tumors collected after 2 weeks of observation. Scale bar: 200 µm.

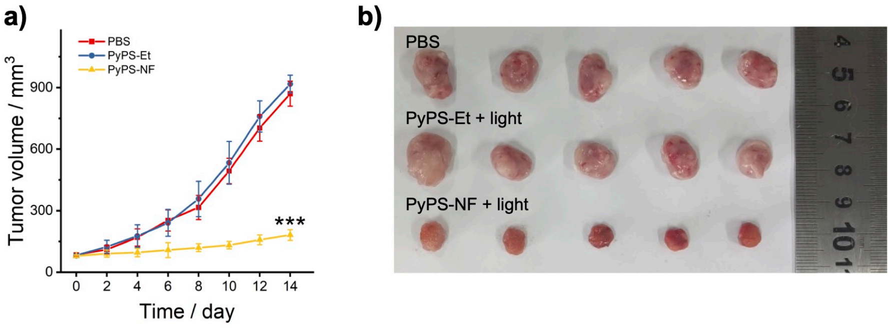

Figure S41. (a) Size of the xenografted tumors in different treatment groups (n = 5). (b) Photographs recorded for the tumors collected after 2 weeks of observation. ***p < 0.001 (one way ANOVA).

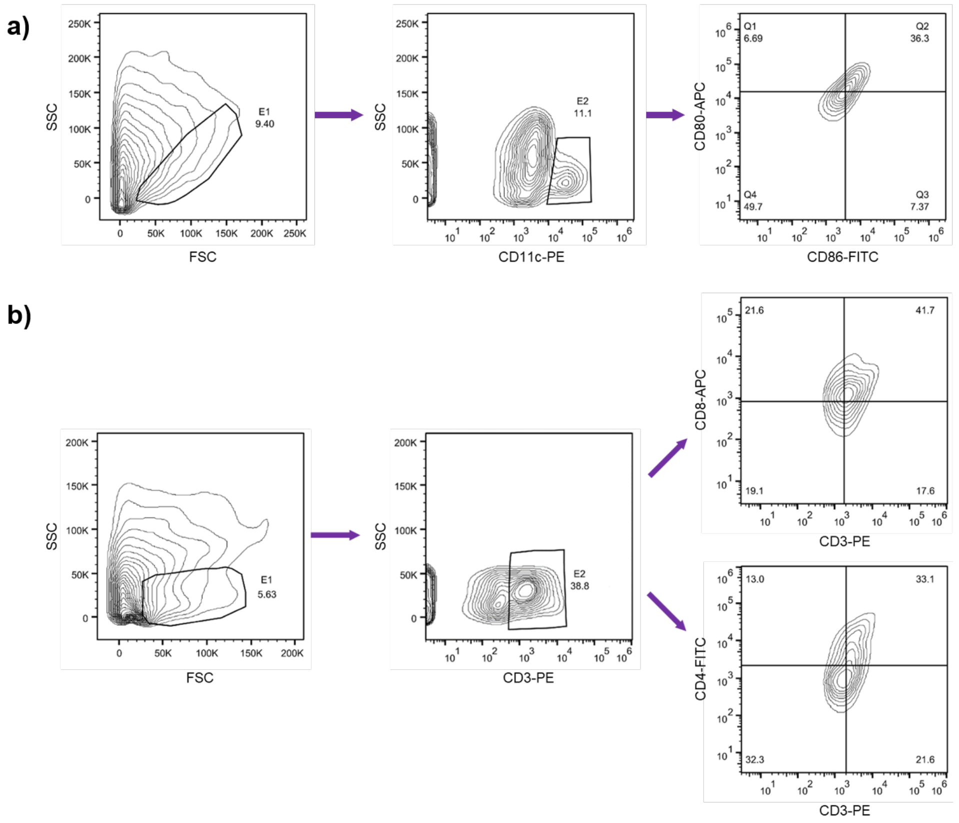

Figure S42. Gating strategy for flow cytometry analysis of (a) maturated DCs and (b) T cells in the primary tumors.

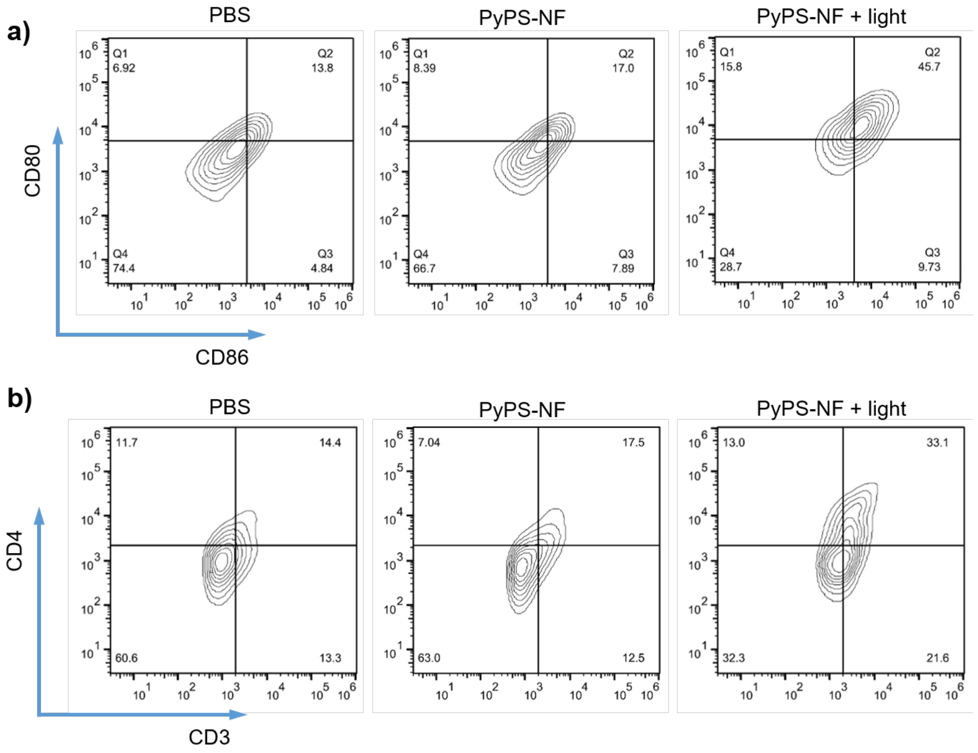

Figure S43. Flow cytometric analysis of (a) maturated dendritic cells (DCs) ($CD80^+CD86^+CD11c^+$) in the lymph nodes of 4T1-tumor-bearing mice and (b) helper T cells ($CD3^+CD4^+$) in the primary tumors of 4T1-tumor-bearing mice after different treatments.

9. NMR and HRMS Spectra

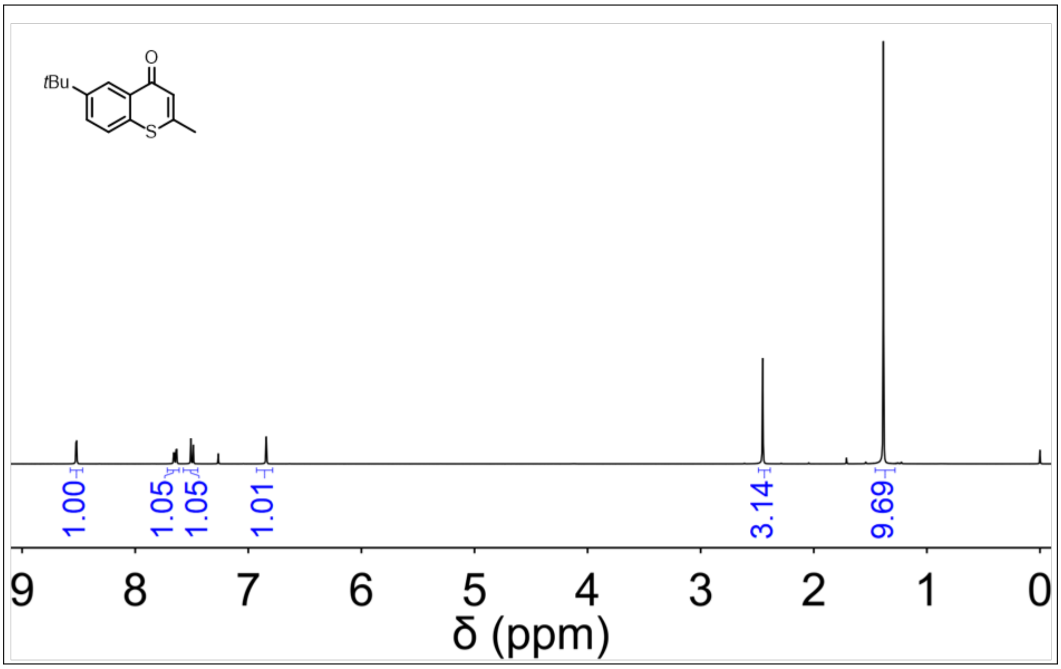

Figure S44. ^{1}H NMR spectrum (400 MHz) of compound **1** in $CDCl_3$ at 25 ºC.

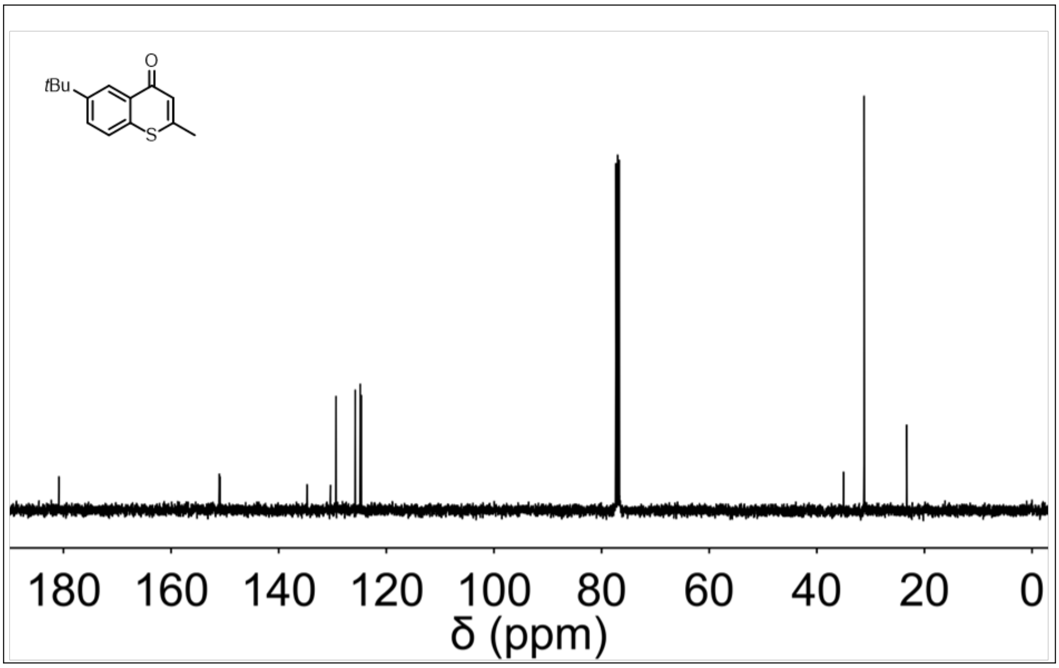

Figure S45. ^{13}C NMR spectrum (100 MHz) of compound **1** in $CDCl_3$ at 25 ºC.

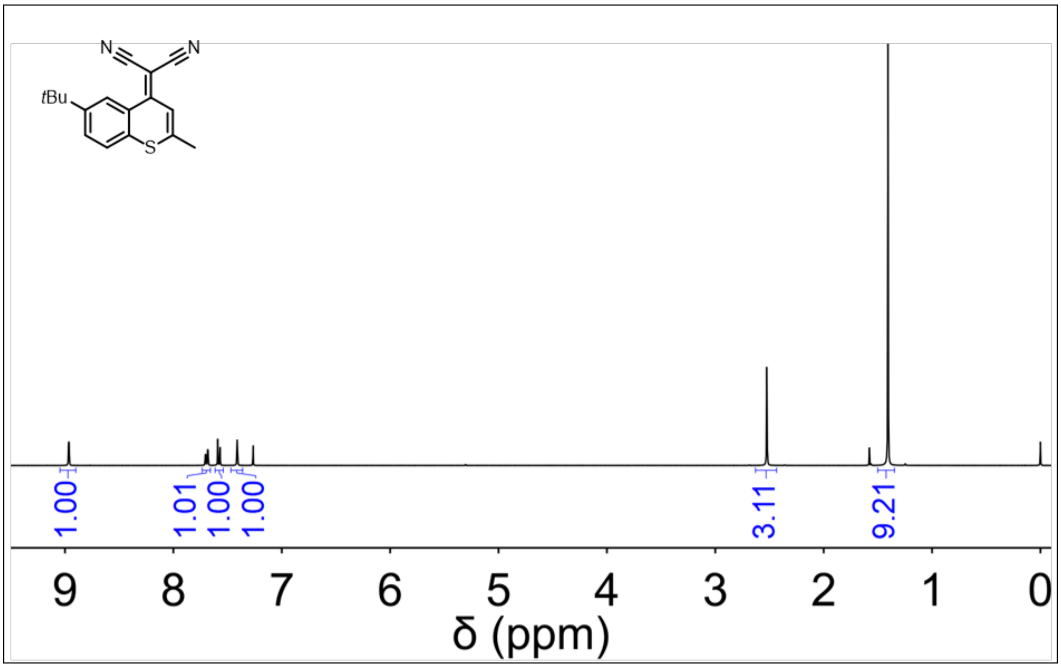

Figure S46. ^{1}H NMR spectrum (400 MHz) of compound **2** in $CDCl_3$ at 25 ºC.

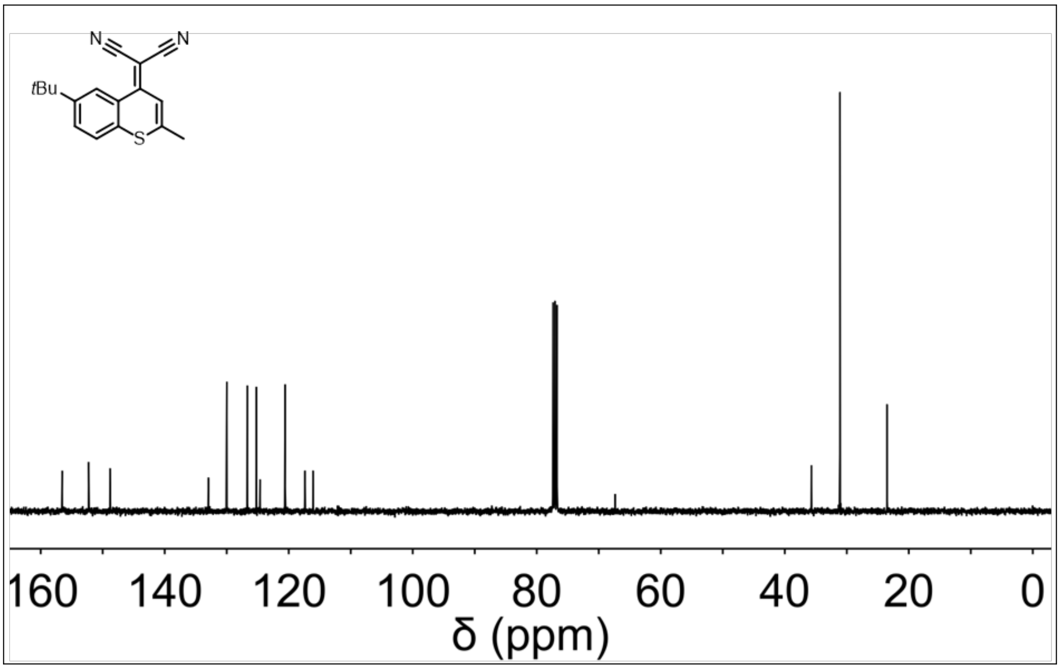

Figure S47. ^{13}C NMR spectrum (100 MHz) of compound **2** in $CDCl_3$ at 25 ºC.

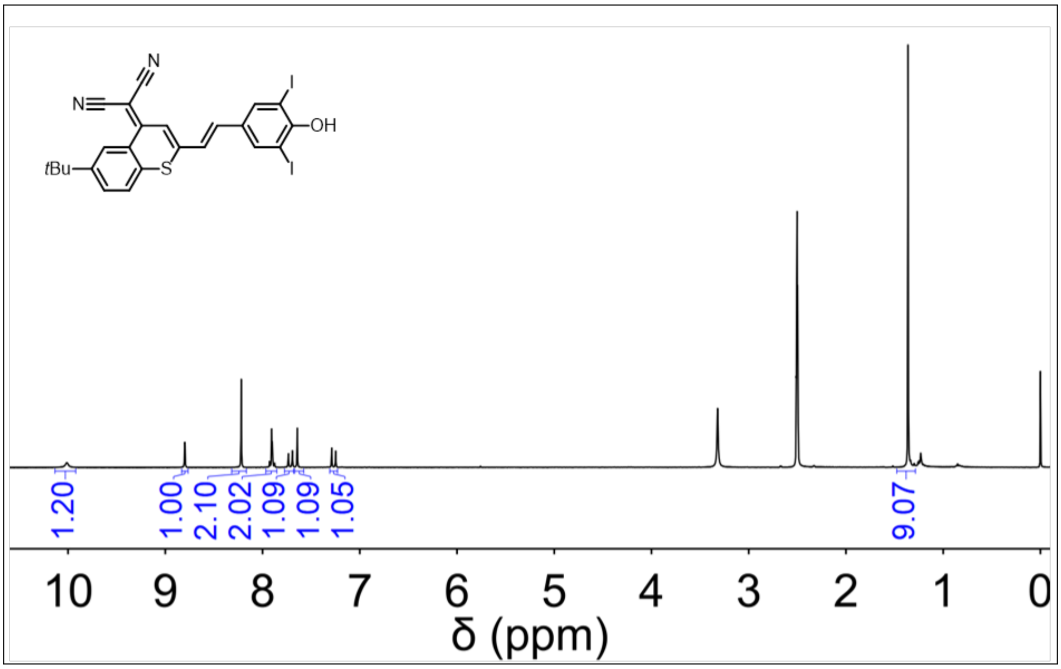

Figure S48. ^{1}H NMR spectrum (400 MHz) of **PyPS-1** in DMSO-d_6 at 25 ºC.

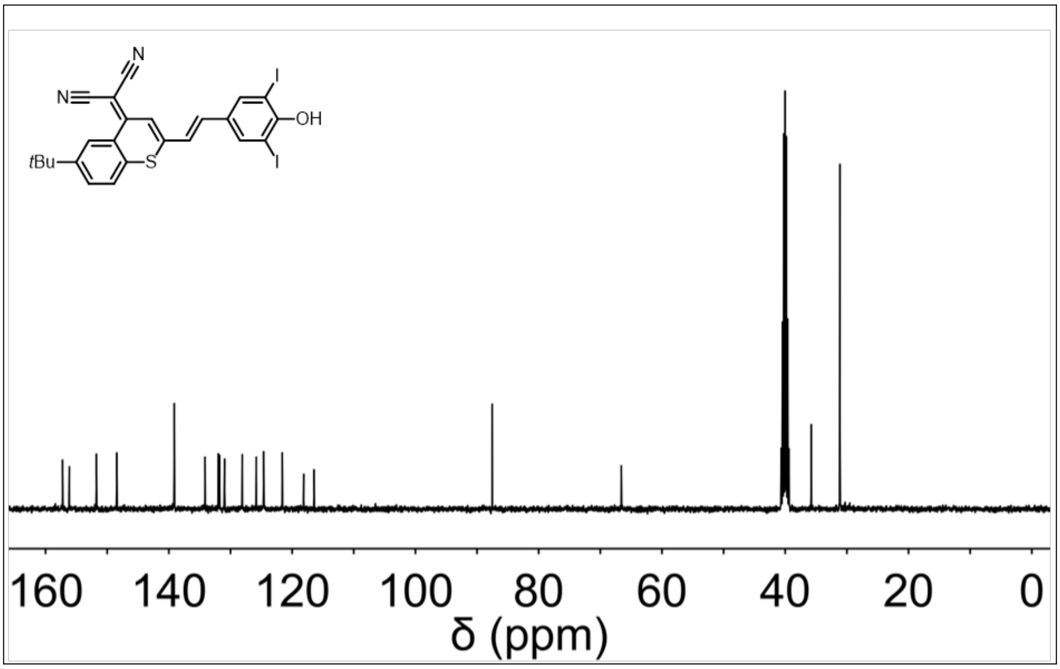

Figure S49. ^{13}C NMR spectrum (100 MHz) of **PyPS-1** in DMSO-d_6 at 25 ºC.

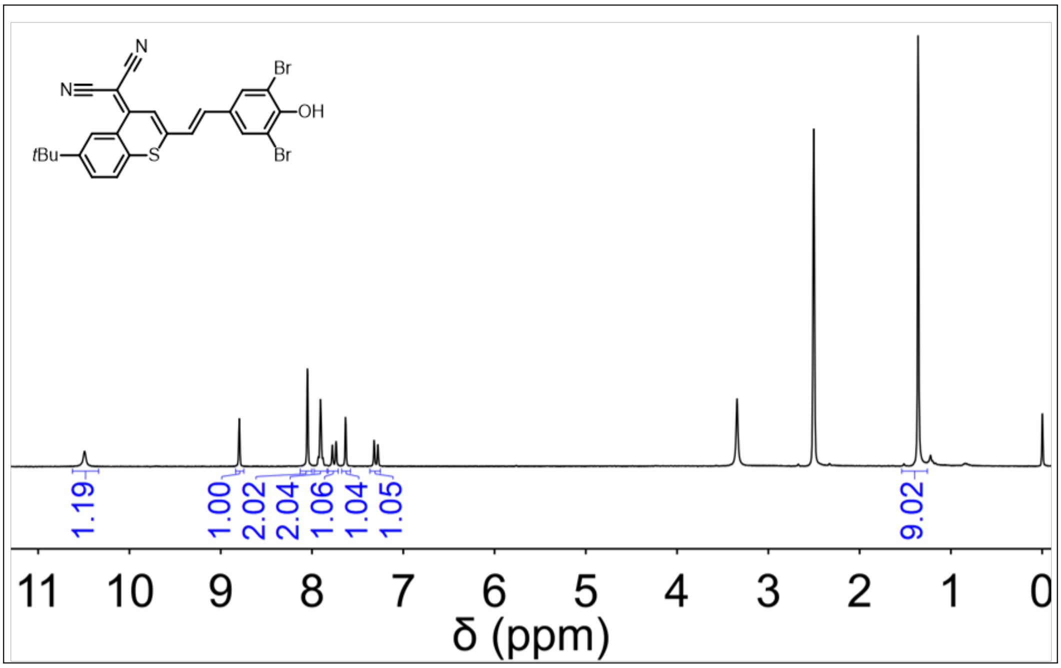

Figure S50. ^{1}H NMR spectrum (400 MHz) of **PyPS-2** in DMSO-d_6 at 25 ºC.

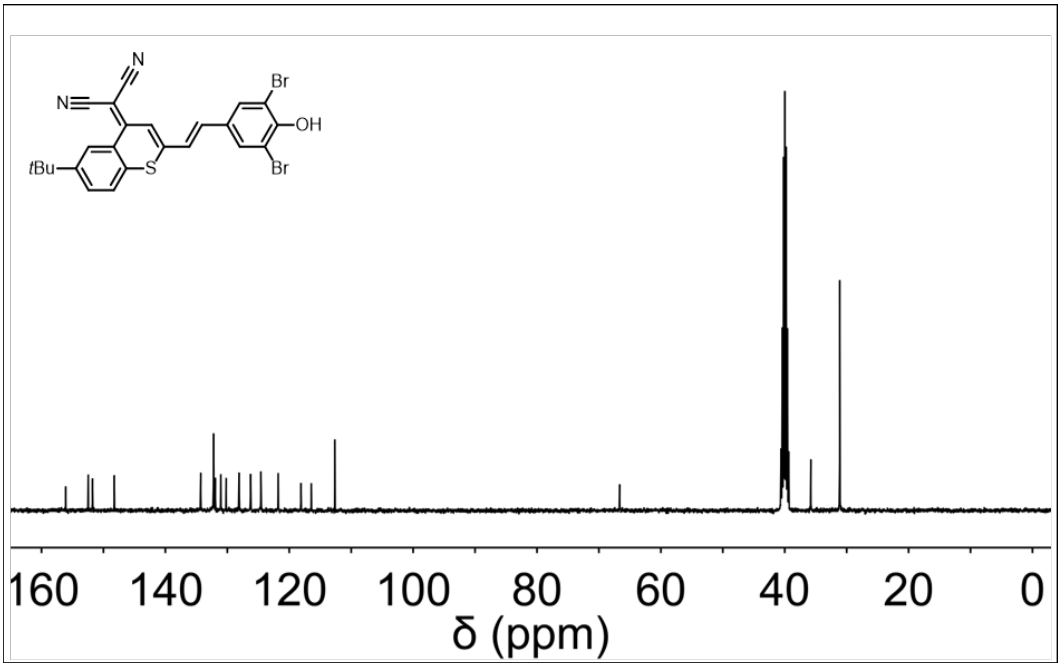

Figure S51. ^{13}C NMR spectrum (100 MHz) of **PyPS-2** in DMSO-d_6 at 25 ºC.

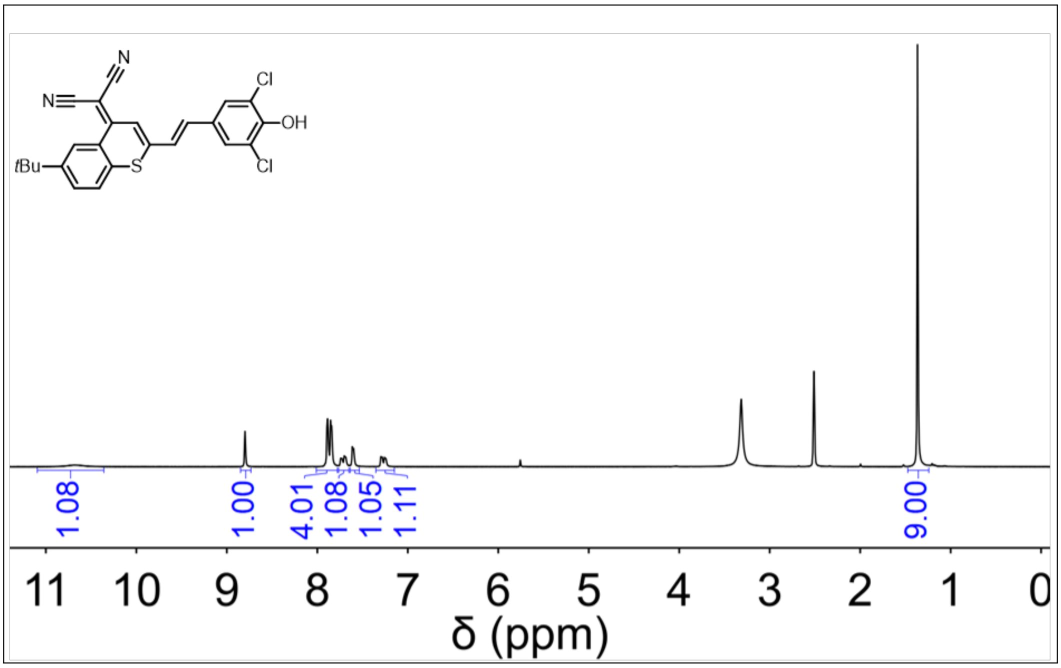

Figure S52. ^{1}H NMR spectrum (400 MHz) of **PyPS-3** in DMSO-d_6 at 25 ºC.

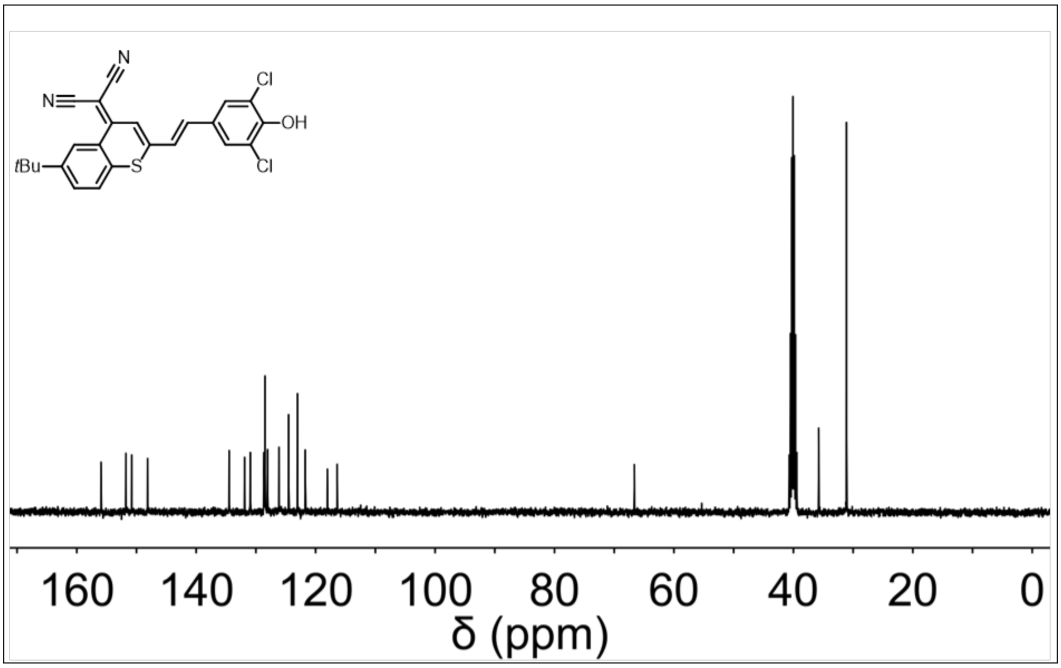

Figure S53. ^{13}C NMR spectrum (100 MHz) of **PyPS-3** in DMSO-d_6 at 25 ºC.

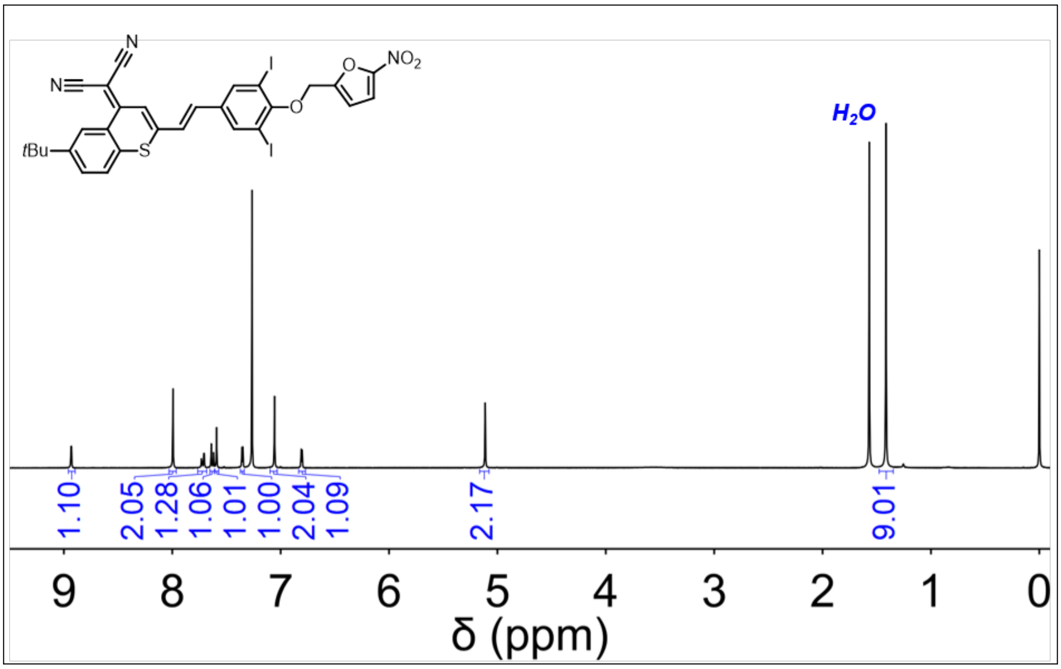

Figure S54. ^{1}H NMR spectrum (400 MHz) of **PyPS-NF** in $CDCl_3$ at 25 ºC.

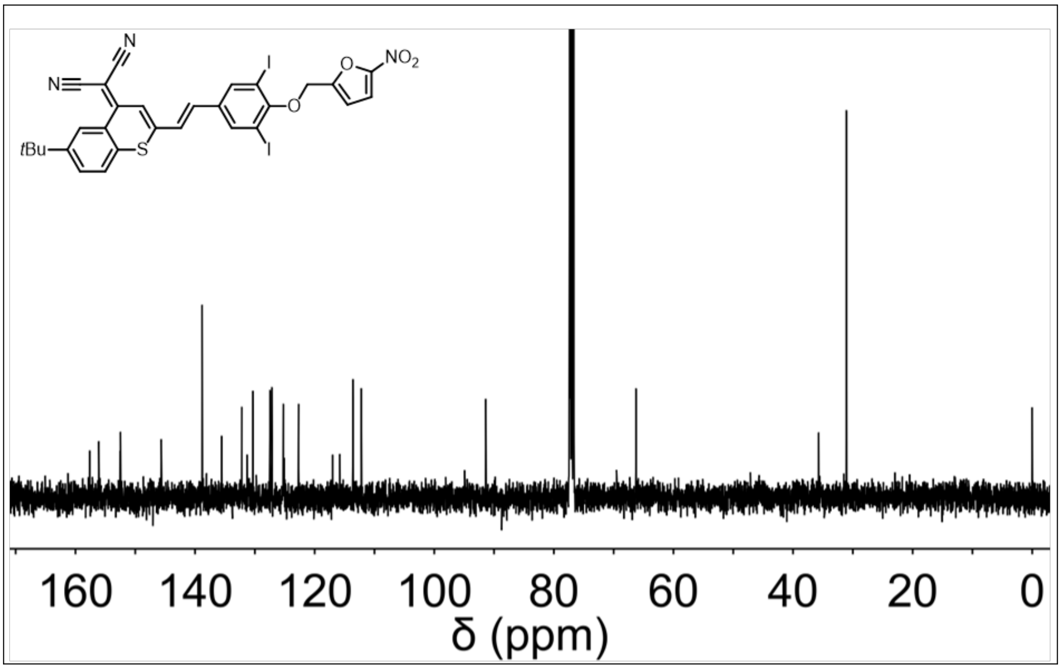

Figure S55. ^{13}C NMR spectrum (100 MHz) of **PyPS-NF** in $CDCl_3$ at 25 ºC.

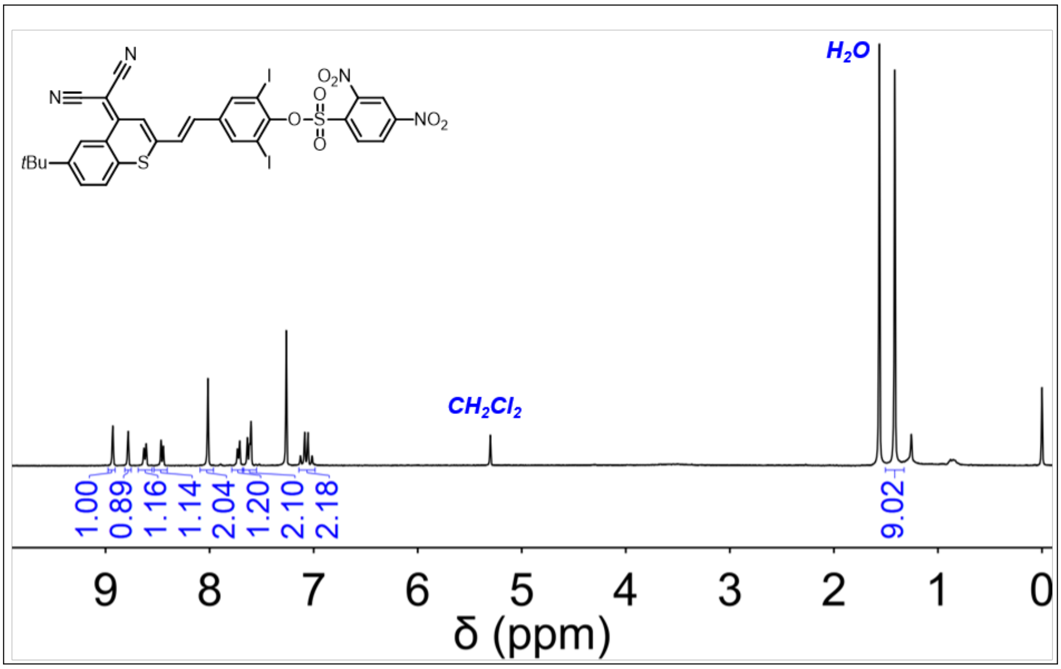

Figure S56. ^{1}H NMR spectrum (400 MHz) of **PyPS-DBS** in $CDCl_3$ at 25 ºC.

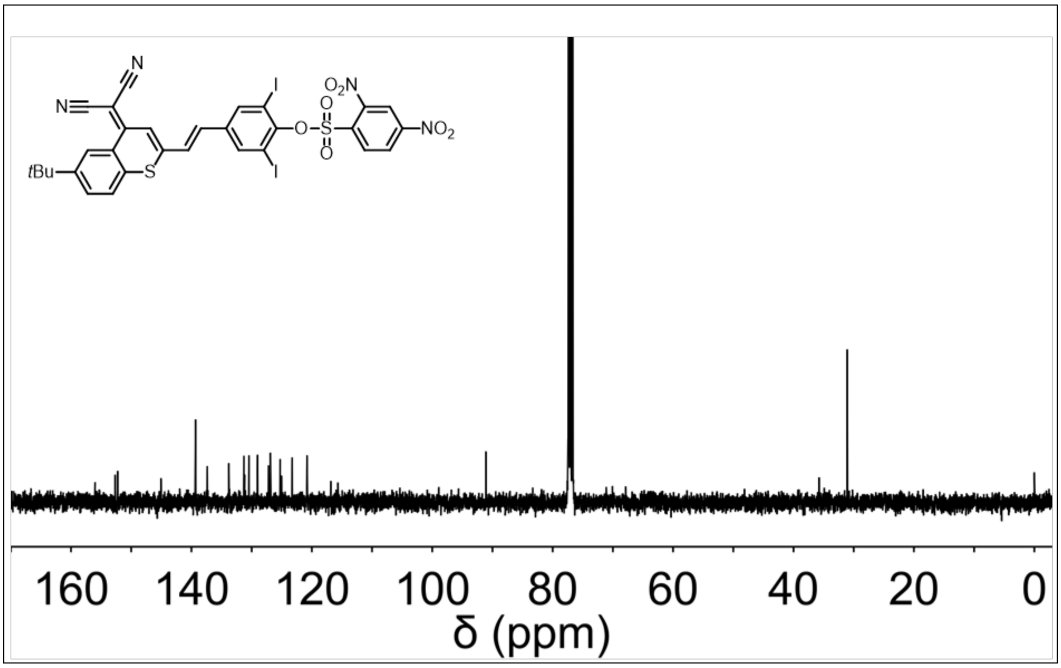

Figure S57. ^{13}C NMR spectrum (100 MHz) of **PyPS-DBS** in $CDCl_3$ at 25 ºC.

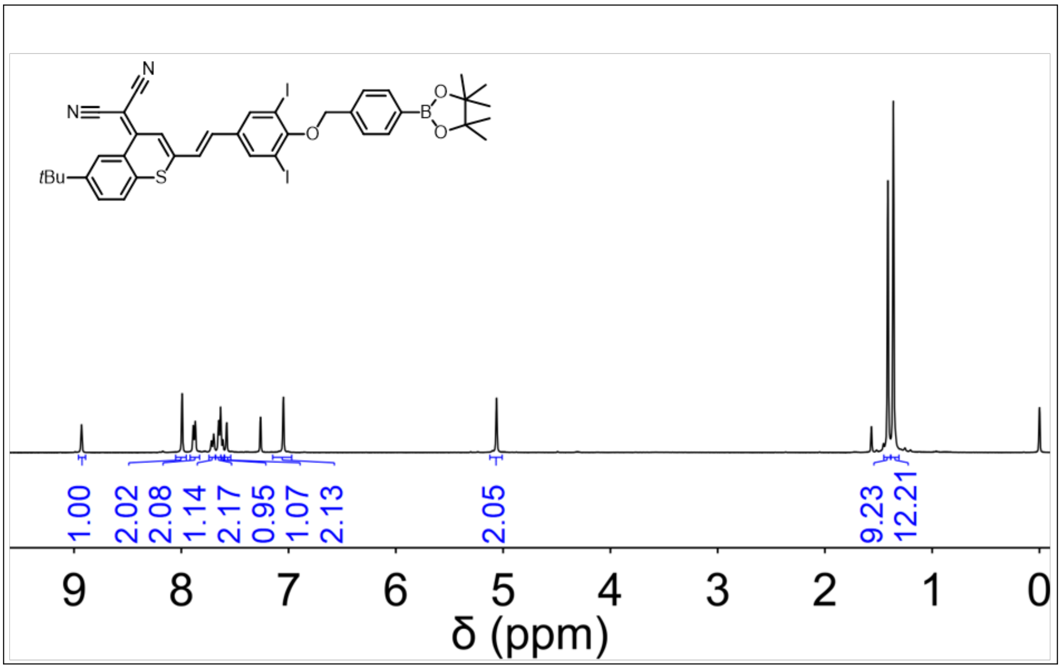

Figure S58. ^{1}H NMR spectrum (400 MHz) of **PyPS-PB** in $CDCl_3$ at 25 ºC.

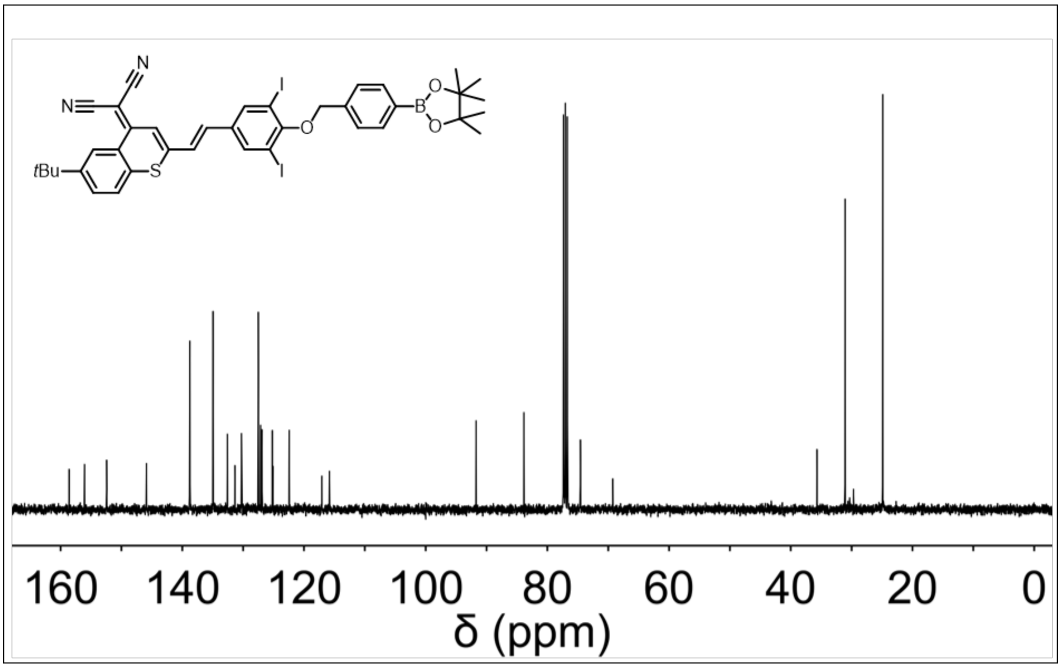

Figure S59. ^{13}C NMR spectrum (100 MHz) of **PyPS-PB** in $CDCl_3$ at 25 ºC.

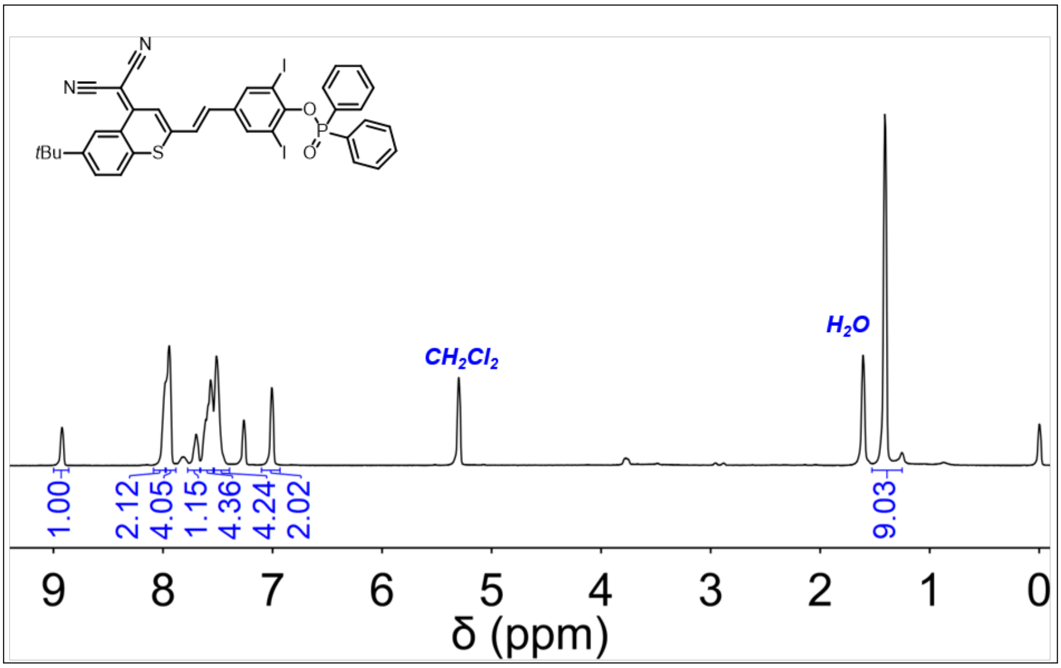

Figure S60. ^{1}H NMR spectrum (400 MHz) of **PyPS-SAR** in $CDCl_3$ at 25 ºC.

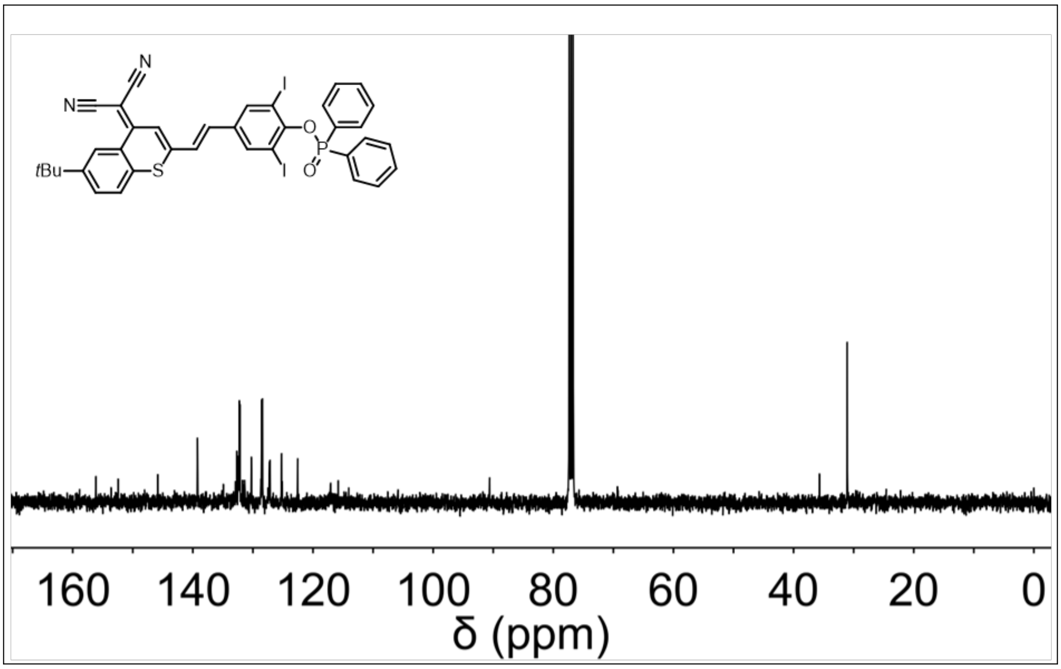

Figure S61. ^{13}C NMR spectrum (100 MHz) of **PyPS-SAR** in $CDCl_3$ at 25 ºC.

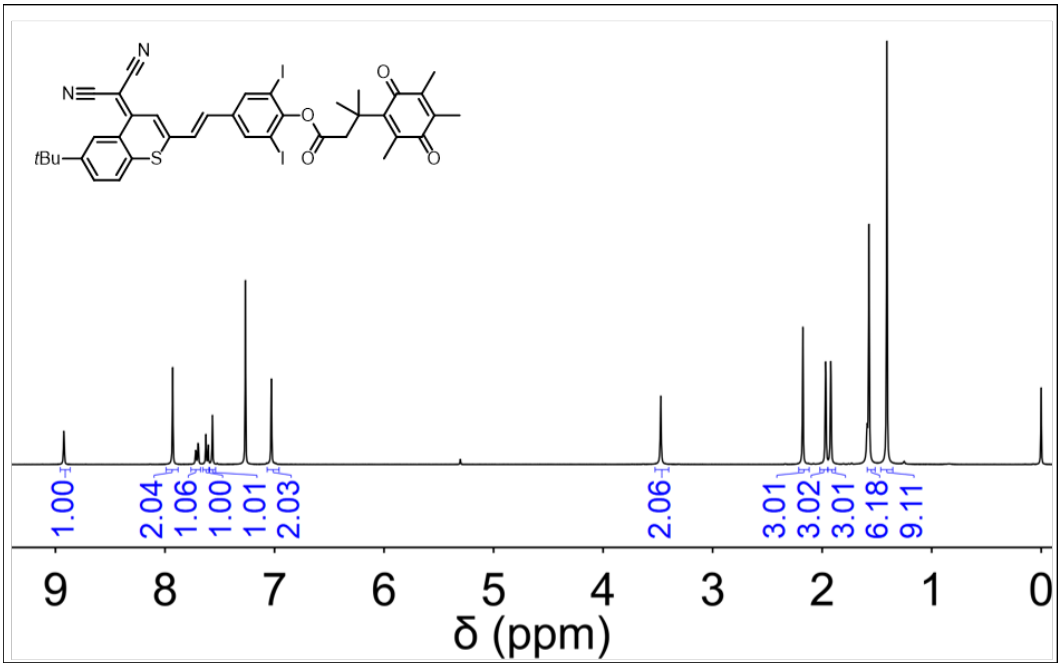

Figure S62. ^{1}H NMR spectrum (400 MHz) of **PyPS-BQ** in $CDCl_3$ at 25 ºC.

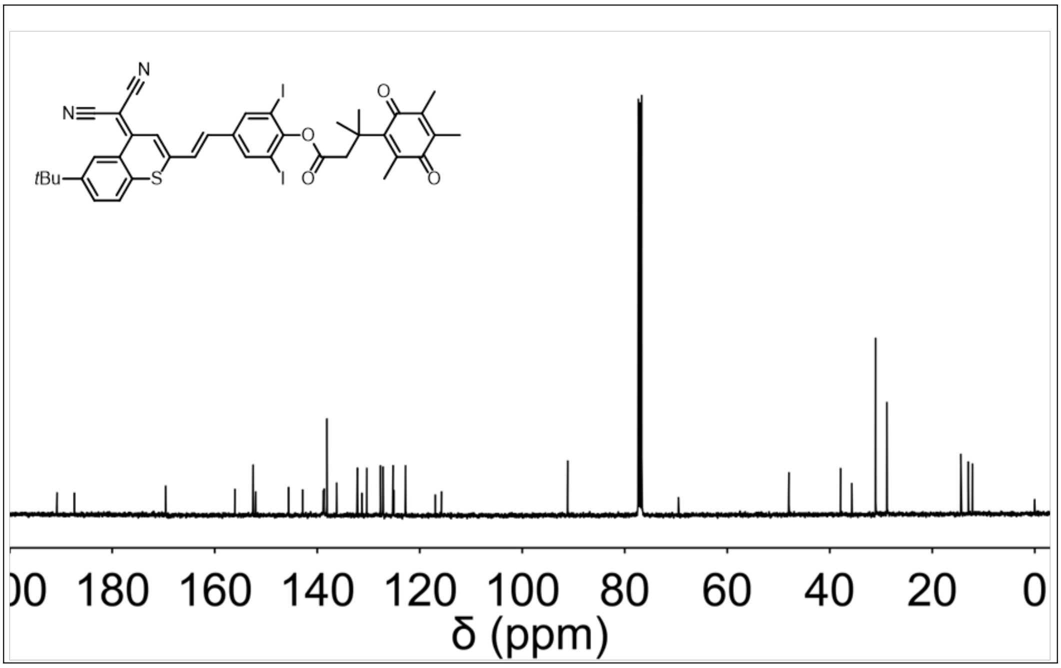

Figure S63. ^{13}C NMR spectrum (100 MHz) of **PyPS-BQ** in $CDCl_3$ at 25 ºC.

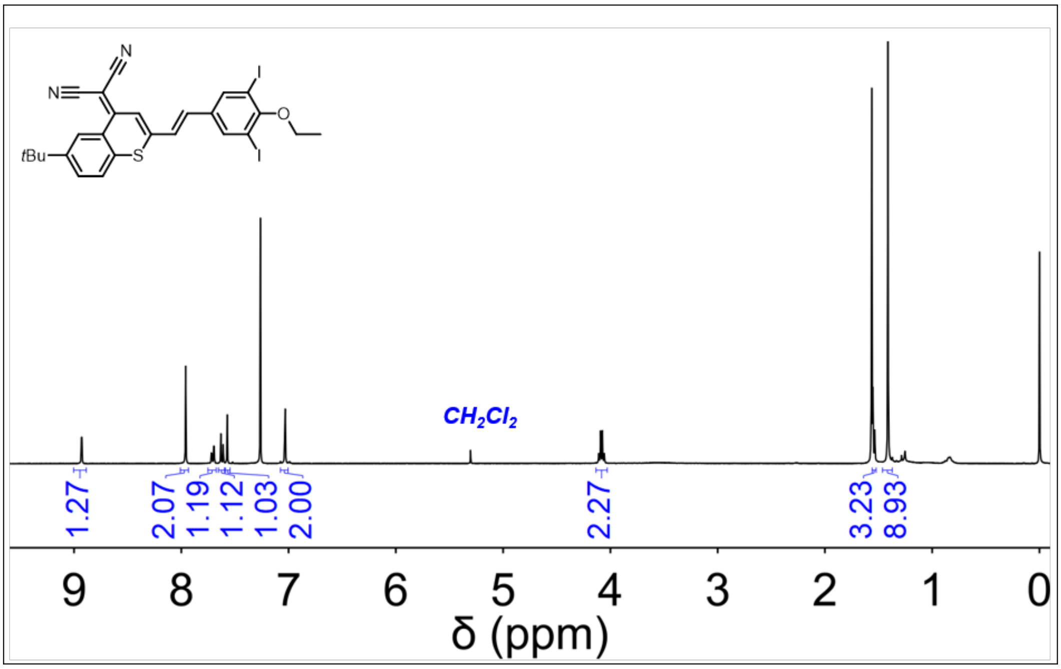

Figure S64. ^{1}H NMR spectrum (400 MHz) of **PyPS-Et** in $CDCl_3$ at 25 ºC.

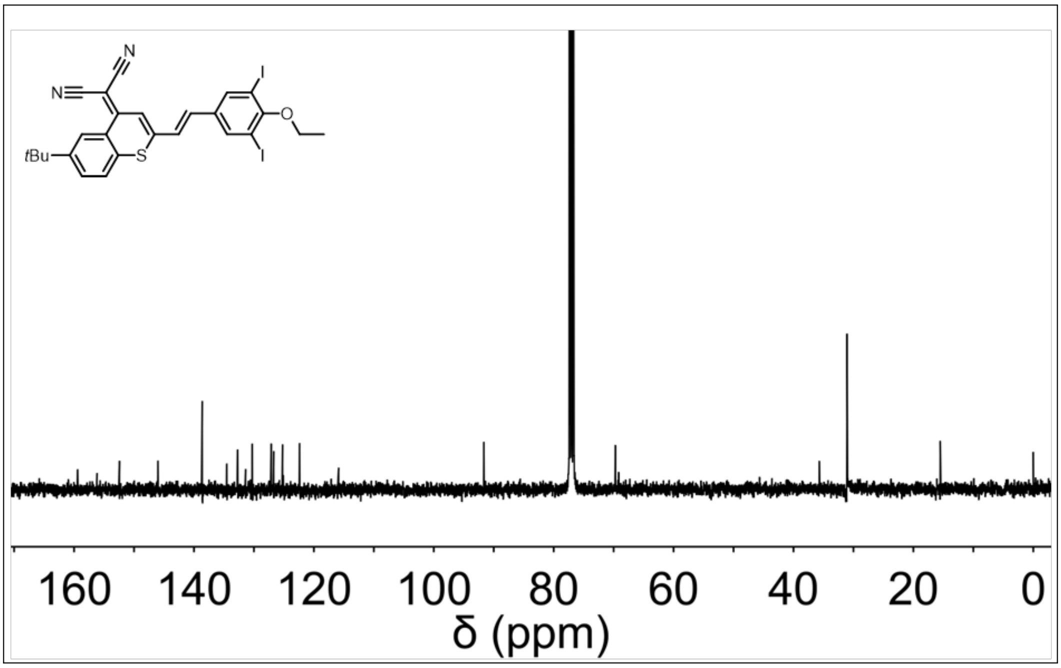

Figure S65. ^{13}C NMR spectrum (100 MHz) of **PyPS-Et** in $CDCl_3$ at 25 ºC.

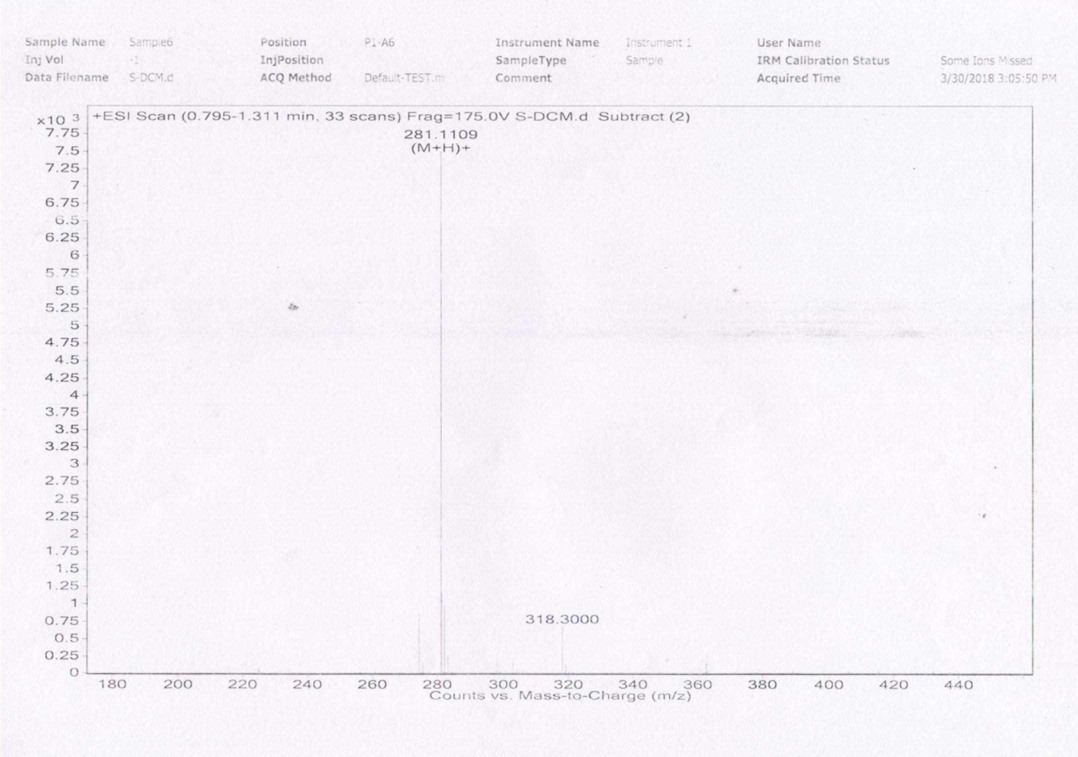

Figure S66. HRMS of compound **2**.

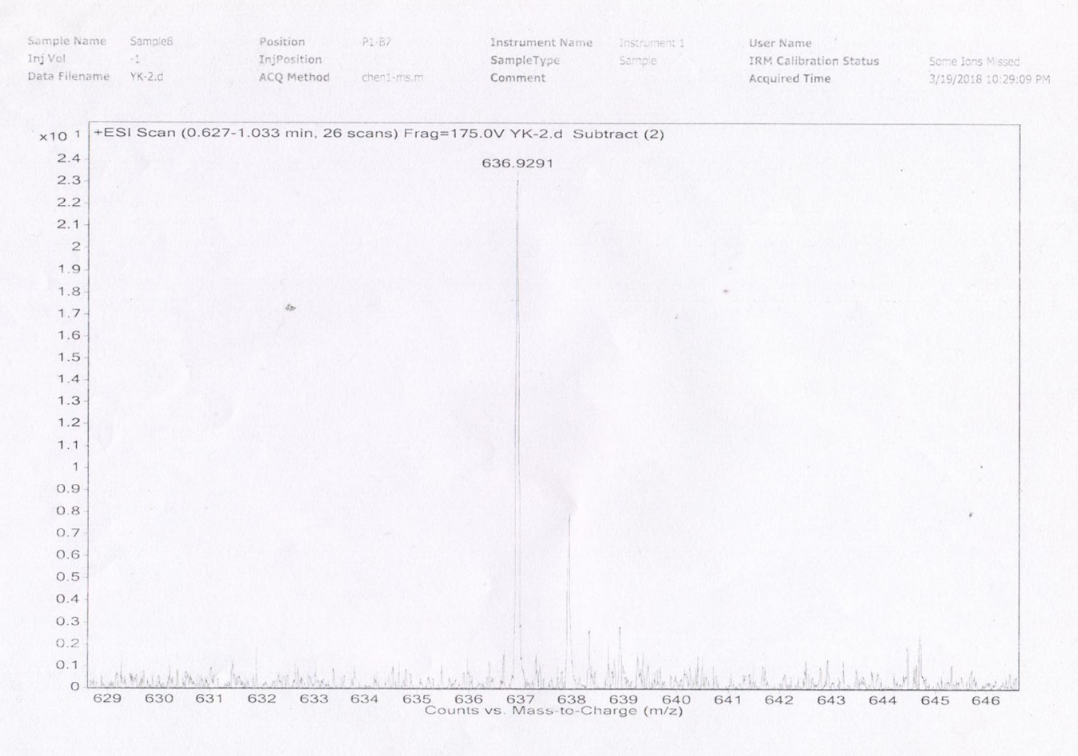

Figure S67. HRMS of **PyPS-1**.

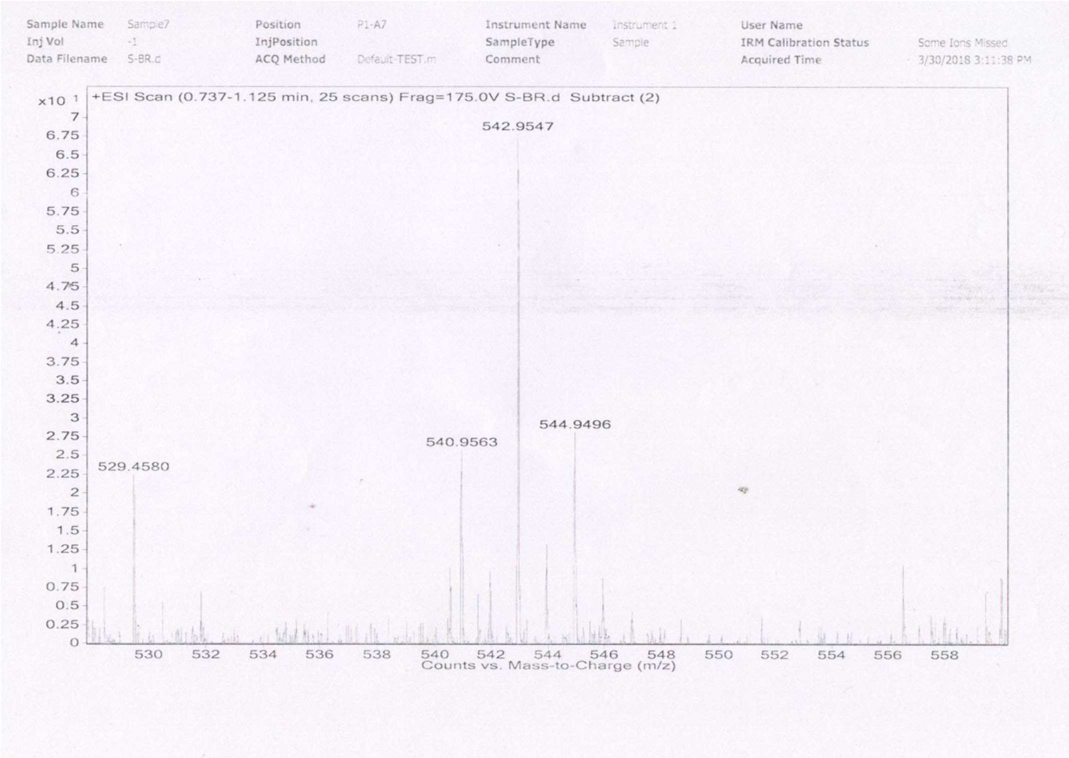

Figure S68. HRMS of **PyPS-2**.

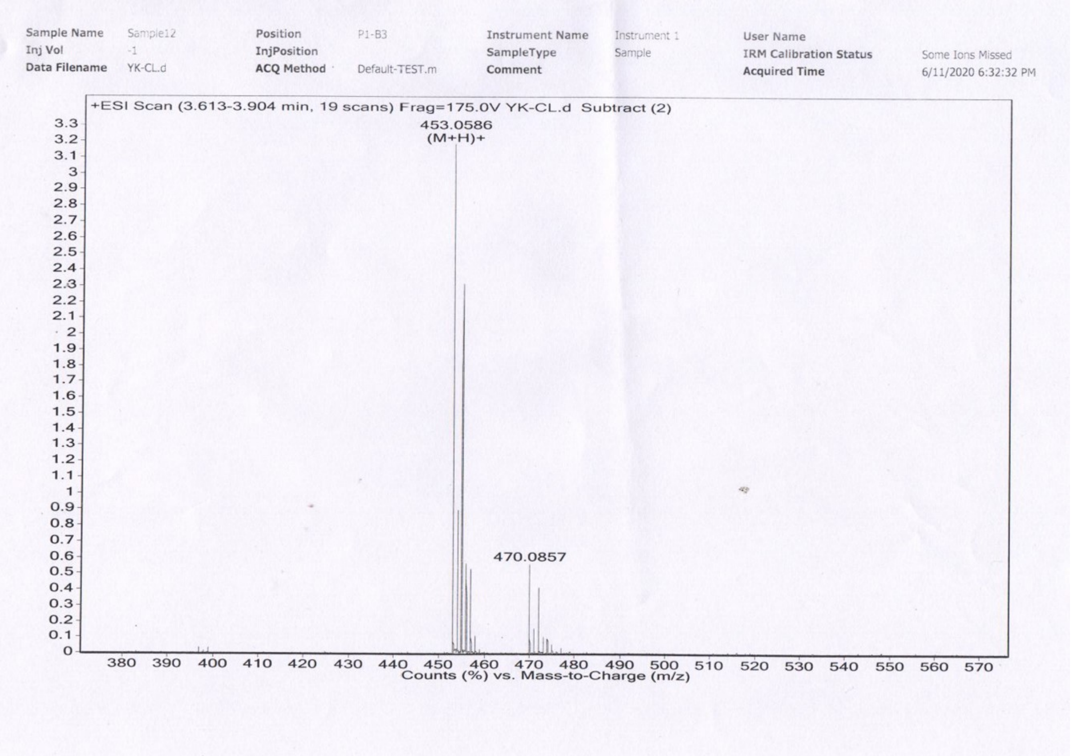

Figure S69. HRMS of **PyPS-3.**

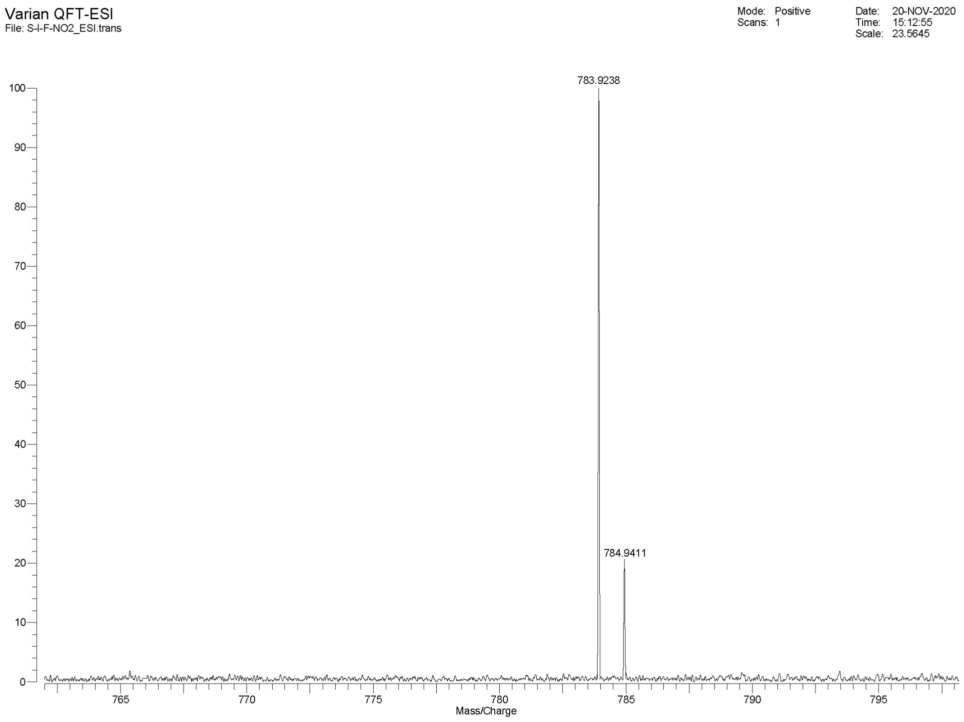

Figure S70. HRMS of **PyPS-NF**.

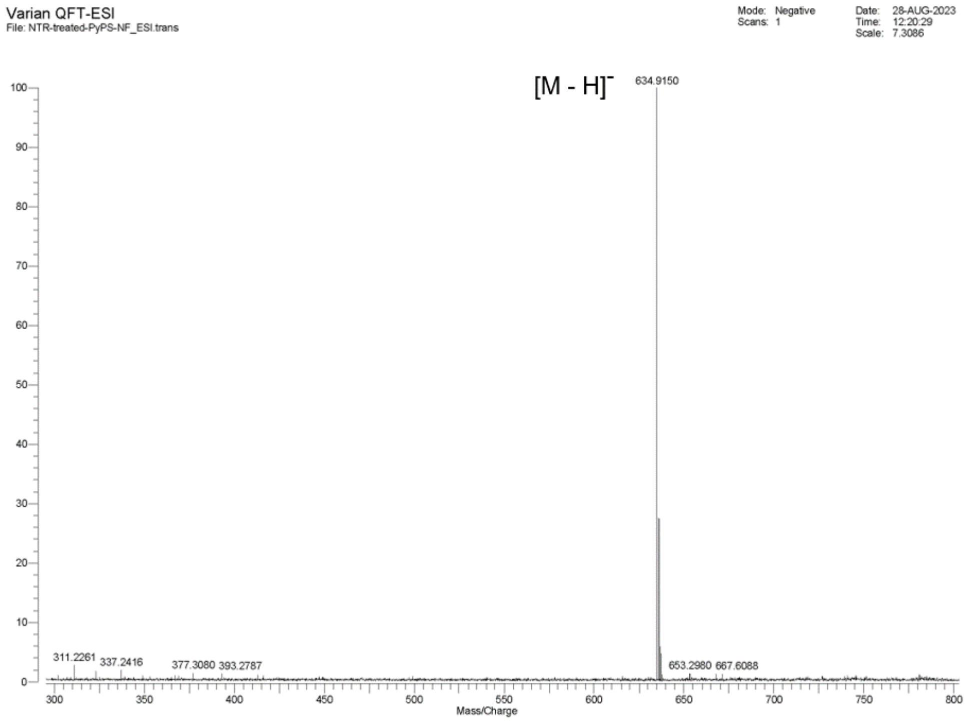

Figure S71. HRMS of resulting products in NTR response experiment.

10. References

(1) W. L. F. Armarego, D. D. Perrin, *Purification of Laboratory Chemicals*, Butterworth-Heinemann, **2009**.

(2) W. Zhai, Y. Zhang, M. Liu, H. Zhang, J. Zhang, C. Li, *Angew. Chem., Int. Ed.* **2019**, *58*, 16601–16609.

(3) H. Zhang, X. Xia, H. Zhao, G. -N. Zhang, D. -Y. Jiang, X. -Y. Xue, J. Zhang, *Dyes and Pigments* **2019**, *163*, 183–189.

(4) M. Su, S. Li, H. Zhang, J. Zhang, H. Chen, C. Li, *J. Am. Chem. Soc.* **2019**, *141*, 402–413.

(5) S. M. Taimoory, S. I. Sadraei, R. A. Fayoumi, S. Nasri, M. Revington, J. F. Trant, *J. Org. Chem.* **2018**, *83*, 4427–4440.

(6) S. Emami, N. Shahrokhirad, A. Foroumadi, M. A. Faramarzi, N. Samadi, N. Soltani-Ghofrani, *Med. Chem. Res.* **2013**, *22*, 5940–5947.

(7) H. Zheng, Q. Wang, Y. Long, H. Zhang, X. Huang, R. Zhu, *Chem. Commun.* **2011**, *47*, 10650–10652.

(8) J. Yang, M. Li, W. Zhu, *Res. Chem. Intermed.* **2018**, *44*, 3959–3969.

(9) A. R. Lippert, E. J. New, C. Chang, J. *J. Am. Chem. Soc.* **2011**, *133*, 10078.

(10) W. Sup. Shin, M. -G. Lee, P. Verwilst, J. H. Lee, S. -G. Chi, J. S. Kim, *Chem. Sci.* **2016**, *7*, 6050–6059

(11) G. M. Morris, R. Huey, W. Lindstrom, M. F. Sanner, R. K. Belew, D. S. Goodsell, A. J. Olson, *J. Comput. Chem.* **2009**, *30*, 2785–2791.
